# A novel three-component reaction between isocyanides, alcohols or thiols and elemental sulfur: a mild, catalyst-free approach towards *O*-thiocarbamates and dithiocarbamates

**DOI:** 10.3762/bjoc.15.155

**Published:** 2019-07-10

**Authors:** András György Németh, György Miklós Keserű, Péter Ábrányi-Balogh

**Affiliations:** 1 Hungarian Academy of Sciences, Research Centre for Natural Sciences, Institute of Organic Chemistry, Medicinal Chemistry Research Group, 1519 Budapest, POB 286, Hungary

**Keywords:** ditihiocarbamate, elemental sulfur, multicomponent reaction, one-pot, thiocarbamate

## Abstract

A new multicomponent reaction has been developed between isocyanides, sulfur and alcohols or thiols under mild reaction conditions to afford *O*-thiocarbamates and dithiocarbamates in moderate to good yields. The one-pot reaction cascade involves the formation of an isothiocyanate intermediate, thus a catalyst-free synthesis of isothiocyanates, as valuable building blocks from isocyanides and sulfur is proposed, as well. The synthetic procedure suits the demand of a modern organic chemist, as it tolerates a wide range of functional groups, it is atom economic and easily scalable.

## Introduction

*O*-Thiocarbamates belong to a class of important biologically active molecules, used mainly as fungicides [1–3] in agricultural and pharmaceutical fields. In particular, recently antitumor [4], anesthetic [5] and enzyme inhibitory effects were discovered, including HIV-1 reverse transcriptase inhibition activity [6–11]. Moreover, their utilization as highly regio- and stereoselective organocatalysts in specific types of chemical tranformations [12–17] was introduced, as well. More recently, *O*-thiocarbamates have been used as H_2_S donors in biological systems [18] and as intermediates in pharmaceutically significant organic syntheses [19–20]. The dithiocarbamate structural moiety can be found in biologically active molecules widely applied as fungicides, herbicides, pesticides [21–25] and in some cases as enzyme inhibitors [26] or antitumor agents [27]. These species are also used as valuable synthetic intermediates [28] and chemosensors for mercury and silver [29–30].

**Scheme 1:**
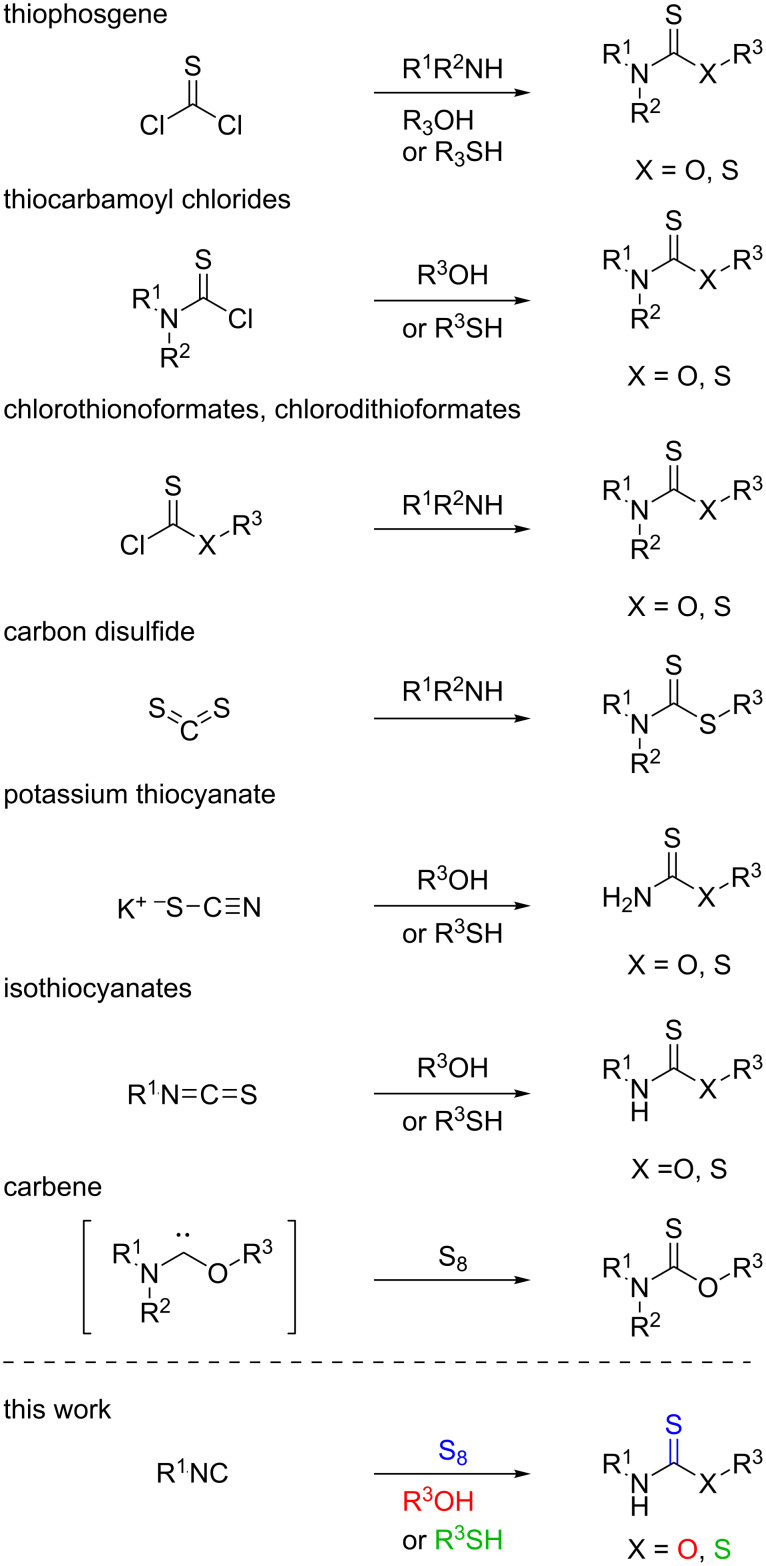
Synthetic routes to *O*-thiocarbamates and dithiocarbamates.

The general methods for the synthesis of *O*-thiocarbamates and dithiocarbamates traditionally rely on substitution reactions of the corresponding halogenated precursors, including thiophosgene [31–33], thiocarbamoyl chlorides [34–37], chlorothionoformates or chlorodithioformates [38–41] providing the appropriate thiocarbamate analogues in good yields (Scheme 1). However, these methods suffer from the formation of toxic, malodorous and/or extremely corrosive byproducts generated by the elimination of the halogen atoms. One should note that the application of these halogenated thiocarbonic acid derivatives might be dangerous and require thorough precaution. Considering dithiocarbamates, a number of methods are based on the reaction of amines and the readily available, but toxic and volatile carbon disulfide [42–45]. Greener methods for the synthesis of thiocarbamates and dithiocarbamates have been developed such as the addition of the amine component to potassium thiocyanate [46–47] or isothiocyanate [48–52] showing better atom economy. Nonetheless, only a few examples can be found in the literature starting from thiocyanates, and regarding the isothiocyanates the preparation of the reagent is required as an additional reaction step before.

The synthesis of isothiocyanates generally relies on the reaction between thiophosgene and amines, thus involves the use of a highly toxic reagent with narrow functional group compatibility [53–56]. Various thiocarbonyl transfer reagents have been developed in the last decades to overcome these drawbacks, such as thiocarbonyl-diimidazole or di-2-pyridyl thionocarbonate [57–58]. Decomposition of dithiocarbamate salts or thiocarbamates with various reagents offers a good alternative [59–62] as well, however, this approach first requires the synthesis of the appropriate precursor. Nitrile oxides react with thiourea to afford isothiocyanate and harmless urea [63–65], but one should note that the instability of the nitrile oxides leads to many byproducts, turning this approach less attractive. The synthesis of isothiocyanates starting from isonitriles involves sulfur-containing reagents such as thallium thiocarboxylates or thiols in the presence of radical initiators [66–68]. All the previously reported methods for the synthesis of *O*-thiocarbamates, dithiocarbamates and isothiocyanates start from toxic and/or unstable reagents, generate halogen waste or have narrow functional group tolerance. The bench-stable, environmentally benign, cheap and nontoxic elemental sulfur offers an alternative starting material to integrate sulfur into the product [69]. For a single molecule, Tan and co-workers showed isothiocyanate might be formed from an isocyanide by elemental sulfur in the presence of a base in low yield [70]. In certain cases, sulfur can be trapped by in situ generated carbenes to afford *O*-thiocarbamates [71–72]. Thioureas and *S*-thiocarbamates are also accessible through multicomponent reactions starting from isocyanides and sulfur [73–75]. The cumbersome synthesis of isothiocyanates from isocyanides and sulfur [76] can be enhanced using various catalysts such as selenium, molybdenum, copper, rhodium [77–82] or tellurium [83] providing the isothiocyanates in excellent yields. These approaches on the other hand suffer from the use of heavy metals, toxic chalcogens and/or long reaction times. More recently, a novel three-component method has been published starting from readily available amines and sodium bromodifluoracetate [84], but this synthetic route provides halogenated waste, as well. As a continuation of our interest in the development of multicomponent reactions [85–87] and reactions involving sulfur [88], herein, we describe a novel synthesis of *O*-thiocarbamates and dithiocarbamates via a three-component reaction of elemental sulfur, isocyanides and alcohols or thiols (Scheme 1). Moreover, during the investigation of the reaction mechanism, we have identified and improved a catalyst-free method for the preparation of isothiocyanates.

## Results and Discussion

The model reaction of 2,6-dimethylphenyl isocyanide (**1a**), elemental sulfur (S_8_) and methanol (**2a**) was employed to screen for the optimal reaction conditions (Table 1). The reactions were followed by TLC and HPLC–MS. Based on preliminary experiments in our laboratory, the reaction was performed in tetrahydrofuran (THF) at 40 °C for 1 h using a 1.5 equiv excess of sodium hydride as the base, S_8_ and the alcohol component (Table 1, entry 1) resulting in the desired thiocarbamate **3a** in 58% yield. During the purification procedure, the change of the stationary phase for the column chromatography from aluminium oxide to silica, resulted in an increased yield of 72% (Table 1, entry 2). After the optimization of the purification process, the excess of the reagents and the role of the base were studied. Increasing the molar excess of all reagents to 2 equiv provided **3a** in 91% yield (Table 1, entry 3), however, the yield was decreased by the use of a larger excess (Table 1, entry 4). Reducing the amount of the reagents was not helpful (Table 1, entry 5), and one can see that 2.5 equiv of sulfur and methanol did not increase the yields either (Table 1, entry 6). A longer reaction time (2 h), however, enhanced the product yield from 72% (Table 1, entry 2) to 84% (Table 1, entry 7). Thus we have combined this reaction time with the elevated molar excess of the reagents resulting in thiocarbamate **3a** in 94% yield (Table 1, entry 8). The advantageous effect of heating was supported by the decreased yield (72%) obtained when performing the reaction at ambient temperature (Table 1, entry 9). Next, the effect of different solvents was investigated, showing that acetonitrile (MeCN) and 2-methyltetrahydrofuran (MeTHF) proved to be suitable alternatives to THF (Table 1, entries 13 and 15) that might be advantageous considering the wide application of these solvents in industrial production [89–90]. On the contrary, the use of dioxane, methyl *tert*-butyl ether (MTBE), toluene or dichloromethane (DCM) was unfavorable, providing the thiocarbamate in 67%, 29%, 12% and 30% yields, respectively (Table 1, entries 10, 11, 12, and 14). Notably, in the lack of inert atmosphere, the yield decreased to 72%, and unidentified byproducts were detected that might be explained with the decomposition or side reactions of the isocyanide component under air. In the case of using other bases, such as caesium carbonate (Cs_2_CO_3_), diisopropylethylamine (DIPEA) or sodium ethoxide (NaOEt), only the isothiocyanate intermediate of the reaction was isolated (Table 1, entries 17, 18, and 20). However, using diazabicycloundecene (DBU) as the base allowed the formation of the desired product, but only in 39% yield (Table 1, entry 19). Without any basic additive, no reaction occurred (Table 1, entry 21) and the starting compounds were recovered.

**Table 1:** Optimization of the reaction conditions for the synthesis of *O*-thiocarbamates.

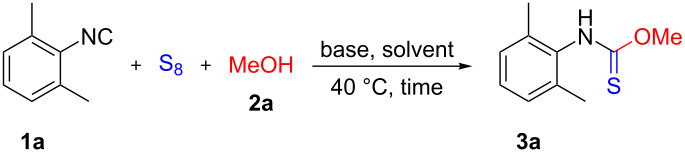					

Entry	Solvent	Base	Time [h]	Molar excess **2a**/S_8_/base	Yield [%]^a,b^

1	NaH	THF	1	1.5:1.5:1.5	58^c^
2	NaH	THF	1	1.5:1.5:1.5	72
3	NaH	THF	1	2:2:2	91
4	NaH	THF	1	2.5:2.5:2.5	80^d^
5	NaH	THF	1	1.5:1.5:2	61
6	NaH	THF	1	2.5:2.5:2	88
7	NaH	THF	2	1.5:1.5:1.5	84
**8**	**NaH**	**THF**	**2**	**2:2:2**	**94^d^**
9	NaH	THF	2	2:2:2	72^e^
10	NaH	dioxane	2	2:2:2	67
11	NaH	MTBE	2	2:2:2	29
12	NaH	toluene	2	2:2:2	12^d^
13	NaH	MeCN	2	2:2:2	92^d^
14	NaH	DCM	2	2:2:2	30
15	NaH	MeTHF	2	2:2:2	87^d^
16	NaH	THF	2	2:2:2	72^f^
17	Cs_2_CO_3_	THF	2	2:2:2	0 (26)^g^
18	DIPEA	THF	2	2:2:2	0 (30)^g^
19	DBU	THF	2	2:2:2	39
20	NaOEt	THF	2	2:2:2	0 (53)^g^
21	–	THF	2	2:2:2	n.r.

^a^Reaction conditions: **1a** (1 mmol), S_8_, **2a**, base, solvent (3 mL), time, under argon atmosphere at 40 °C. ^b^Isolated yields. ^c^Flash column chromatography performed on aluminium oxide as stationary phase. ^d^Average of two runs. ^e^Room temperature. ^f^Lack of inert atmosphere. ^g^Yield of isothiocyanate intermediate. n.r. = no reaction.

**Scheme 2:**
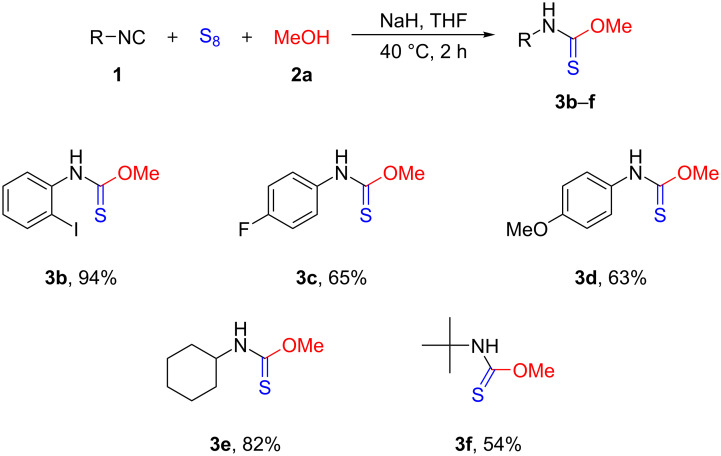
Substrate scope of isocyanides. ^a^Reaction conditions: **1** (1 mmol), S_8_ (2 mmol), **2a** (2mmol), NaH (2 mmol), THF (3 mL), 2 h, under argon atmosphere at 40 °C. Yields refer to isolated yields.

With the optimized reaction conditions in hand, the generality and substrate scope of the reaction using different isocyanides were investigated (Scheme 2). Considering the yield of various aromatic isonitriles, no significant difference was noticed between electron-withdrawing and electron-donating substituents (**3c** and **3d**, respectively). The *ortho*-iodo-substituted **3b** was obtained in an excellent yield (94%) demonstrating that no steric hindrance occurs during the reaction. Taking into account aliphatic derivatives, *O*-methyl cyclohexylcarbamothioate (**3e**) was formed in good yield (82%), however, *O*-methyl *tert*-butylcarbamothioate (**3f**), partly due to the volatile nature of the isocyanide and the thiocarbamate as well, was obtained only in 54% yield.

**Scheme 3:**
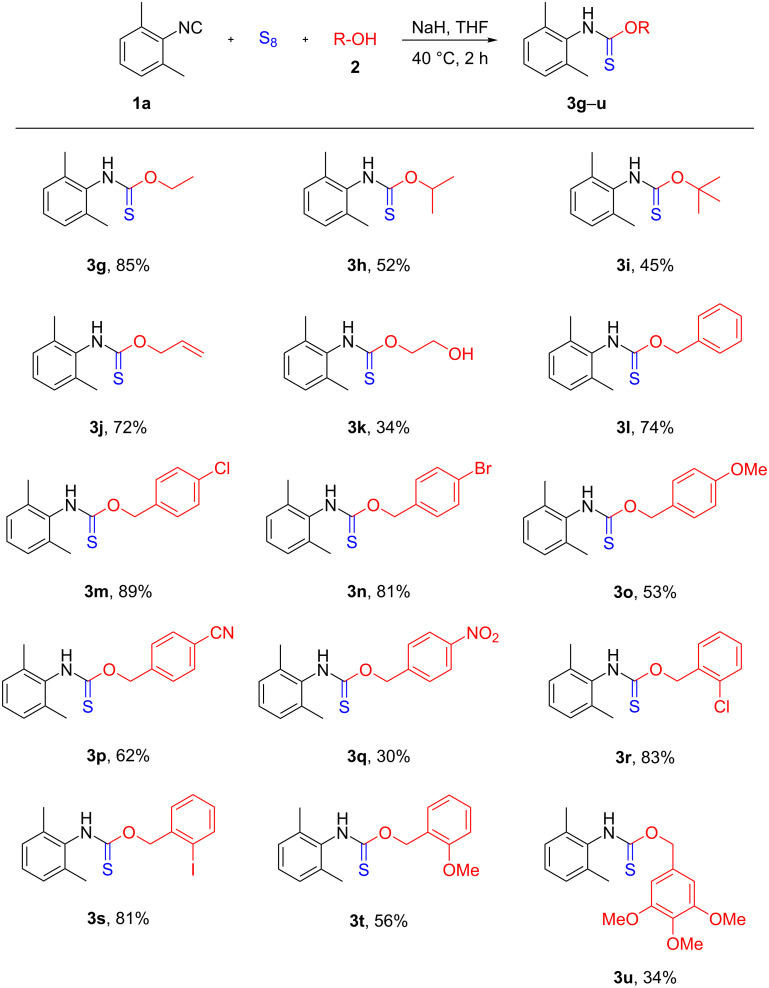
Substrate scope of alcohols. Reaction conditions: **1a** (1 mmol), S_8_ (2 mmol), **2** (2mmol), NaH (2 mmol), THF (3 mL), 2 h, under argon atmosphere at 40 °C. Yields refer to isolated yields.

In order to further explore the scope of the reaction, different alcohols were tested (Scheme 3). Regarding the compounds **3g**–**i** it can be noticed that the yield drops from the primary alcohol towards the tertiary one (85%, 52% and 45%, respectively) that might be attributed either to steric hindrance or the growing instability of the conjugate base of the secondary and tertiary alcohol, respectively. The present method provided the allylic derivative **3j** in 72% yield, however, applying ethylene glycol resulted in **3k** in 34% yield only. In the latter case no dimeric product but several unidentified side products were detected by TLC and HPLC–MS. Although the full conversion of the isocyanide to the isothiocyanate intermediate was observed by TLC and HPLC–MS, phenol proved to be unreactive under the standard reaction conditions. Consequently, we have turned our attention to different benzylic alcohols that could be utilized to further examine the functional group tolerance of the reaction. Notably, chlorine, bromine and iodine substituents were compatible with the transformation, providing **3m**, **3n**, **3r** and **3s** in 81–89% yield. The nitrile derivative **3p** was obtained successfully in 62% yield, showing the reactivity difference between the cyano and the isocyano groups. Interestingly, methoxy-substituted thiocarbamates **3o** and **3t** were obtained in lower 53% and 56% yield, respectively, that decreased further to 34% in the case of the trimethoxy-substituted product **3u**. As the methoxy group is inert under the standard reaction conditions, one might assume that the electron-donating ability reduces the stability of the in situ-generated anion, just as in the case of the secondary and tertiary alcohols. The nitro derivative **3q** was isolated in 30% yield along with multiple byproducts detected that may be due to possible reductive side-reactions caused by sulfur [91]. To the best of our knowledge, out of the 21 synthesized *O*-thiocarbamate derivatives (Scheme 2 and Scheme 3), 18 compounds are new, and only **3d**, **3e** and **3f** are known in the literature [92–94]. The new derivatives have been characterized by ^1^H NMR, ^13^C NMR, HRMS and melting point. However, the thiocarbamate **3c** happened to be unstable and started to decompose after work-up. Therefore, an HPLC–MS spectrum of the reaction mixture after completion of the reaction and an HRMS of the crude product are attached in Supporting Information File 1.

Several experiments were performed to discover the reaction conditions that enable the synthesis of *O*-aryl thiocarbamates. Initially, DBU was used in refluxing dioxane, as this base was shown to provide the appropriate aliphatic *O*-thiocarbamate. However, in this case only the isothiocyanate intermediate was obtained. Then, trimethylamine was applied in refluxing MeCN [95] or sodium hydroxide in dimethyl sulfoxide (DMSO) at 70 °C. In both cases only the isothiocyanate intermediate and phenol were observed by HPLC–MS but no formation of the desired product.

Then we turned to the synthesis of dithiocarbamate **5a** under the standard reaction conditions, but only the isothiocyanate intermediate was obtained (Table 2, entry 1). Thus a new optimization of the reaction conditions became necessary. Similarly to the previous methodology, the base, the solvent, the temperature and the molar excess of the reagents were changed using the model reaction of 2,6-dimethylphenyl isocyanide (**1a**), sulfur and benzyl mercaptan (**4a**, Table 2). Initially, NaOH was used in DMSO as shown in entry 2 (Table 2), providing the desired dithiocarbamate **5a** in only 22% yield [96]. In order to improve the yield, firstly the temperature was elevated to 70 °C and 100 °C to afford **5a** in 38% and 34% yield, respectively (Table 2, entries 3 and 4). Larger excesses of the reagents and the base cut back the yield to 23% (Table 2, entry 5). Using NaH instead of NaOH did not improve the yield of the reaction (Table 2, entry 6), nor did the use of Cs_2_CO_3_ (Table 2, entry 7). However, using *N*,*N*-dimethylformamide (DMF) or *N*,*N*-dimethylacetamide (DMA) as the solvent provided **5a** in 43% and 45% yield, respectively (Table 2, entries 8 and 9), while on the other hand, *N*-methyl-2-pyrrolidone (NMP) was disadvantageous for the reaction (Table 2, entry 10). It is well-known that at elevated temperatures sulfur may act as an oxidant, which in this case may have compromised the reaction [97–99]. Therefore, the molar excess of sulfur was decreased, providing a positive effect on the reaction affording **5a** in 59% yield.

**Table 2:** Optimization of the reaction conditions for the synthesis of dithiocarbamates.

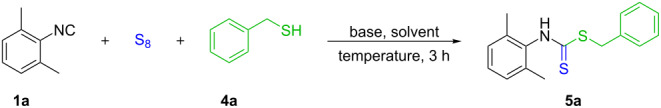					

Entry	Solvent	Base	Temp. [°C]	Molar excess **4a**/S_8_/base	Yield [%]^a,b^

1	NaH	THF	40	2:2:2	0^c^
2	NaOH	DMSO	40	2:2:2	22
3	NaOH	DMSO	70	2:2:2	38
4	NaOH	DMSO	100	2:2:2	34
5	NaOH	DMSO	70	3:3:3	23
6	NaH	DMSO	70	2:2:2	36
7	Cs_2_CO_3_	DMSO	70	2:2:2	14
8	NaOH	DMF	70	2:2:2	43
9	NaOH	DMA	70	2:2:2	45
10	NaOH	NMP	70	2:2:2	34
11	NaOH	DMA	70	2:1.2:2	59

^a^Reaction conditions: **1a** (1 mmol), S_8_, **4a**, base, solvent (3 mL), temperature, 3 h under argon atmosphere. ^b^Isolated yield, unless noted otherwise. ^c^Isothiocyanate intermediate detected by HPLC–MS.

**Scheme 4:**
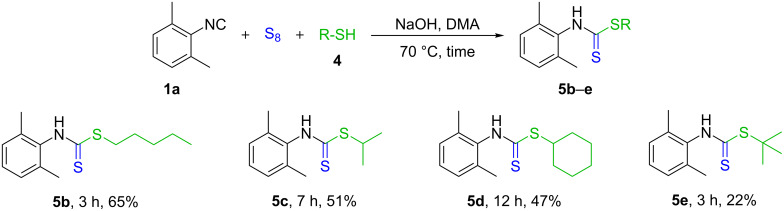
Substrate scope of thiols. Reaction conditions: **1a** (1 mmol), S_8_ (1.2 mmol), **4** (2 mmol), NaOH (2 mmol), DMAc (3 mL), time, under argon atmosphere at 70 °C. Yields refer to isolated yields.

With the optimized reaction conditions in hand, a number of dithiocarbamate derivatives were synthetized (Scheme 4). One might notice the same trend as in the case of thiocarbamates **3g–i**, in particular, the primary mercaptans gave the highest yields (**5a** and **5b**), while in the case of secondary (**5c** and **5d**) and tertiary thiols (**5e**) the products were isolated in lower yields. Although a full conversion of the isocyanide to the isothiocyanate intermediate was observed by TLC and HPLC–MS, thiophenol, likewise to phenol was unreactive under the standard reaction conditions. The generally lower yields, harsher reaction conditions and stronger negative effect of electron-donating groups might be explained with the softer nucleophilicity of the thiols compared to the alcohols [100]. All five dithiocarbamate derivatives synthesized are new and were characterized by ^1^H NMR, ^13^C NMR, HRMS and melting point.

**Scheme 5:**
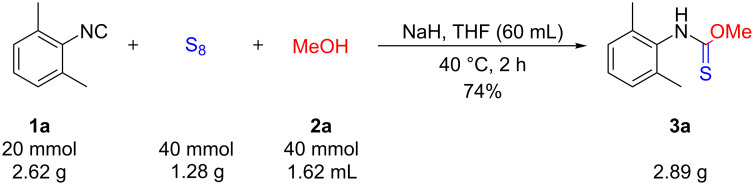
Scaled-up synthesis for **3a**.

After the successful application of various nucleophiles, it was our intention to investigate the scalability of the procedure. Thus, a twenty-fold scale-up of the reaction between the isocyanide **1a**, sulfur and methanol (**2a**) was performed (Scheme 5). In this case, the experimental conditions were necessarily slightly different, as in larger quantities the reaction between the alcohol and NaH needs to be kept under control. Therefore, the mixture of **1a**, methanol and THF was added dropwise to a mixture of NaH and sulfur in THF under ice-cooling. After the work-up, no chromatography was necessary and the crude product was purified by recrystallization from hexane/ethyl acetate. The three collected crops of crystals provided the thiocarbamate **3a** in a total of 74% yield.

**Scheme 6:**
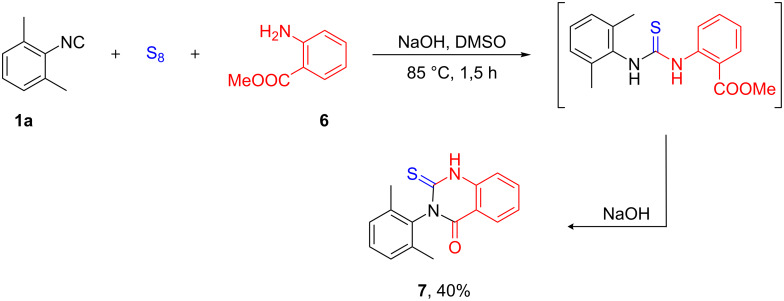
Multicomponent domino synthesis of quinazolinone **7**.

We have envisaged that our multicomponent reaction could be compatible with subsequent one-pot transformations. In order to demonstrate this capability, we performed the multicomponent domino annulation between isocyanide **1a**, sulfur and methyl anthranilate (**6**) in DMSO in the presence of NaOH at 85 °C that provided 3-(2,6-dimethylphenyl)-2-thioxo-2,3-dihydroquinazolin-4(1*H*)-one (**7**), a new quinazolinone derivative in 40% yield (Scheme 6). Notably, these heterocycles are known for their use as antitumor [101], anticonvulsant [102] or epidermal growth factor receptor tyrosine kinase inhibitory agents [103], JNK inhibitors [104] or 5-HT_3_ antagonists [105]. Earlier, a one-pot synthesis of an analogous compound was accomplished by Sayahi et al. starting from isothiocyanates in the presence of CuBr [106].

**Scheme 7:**
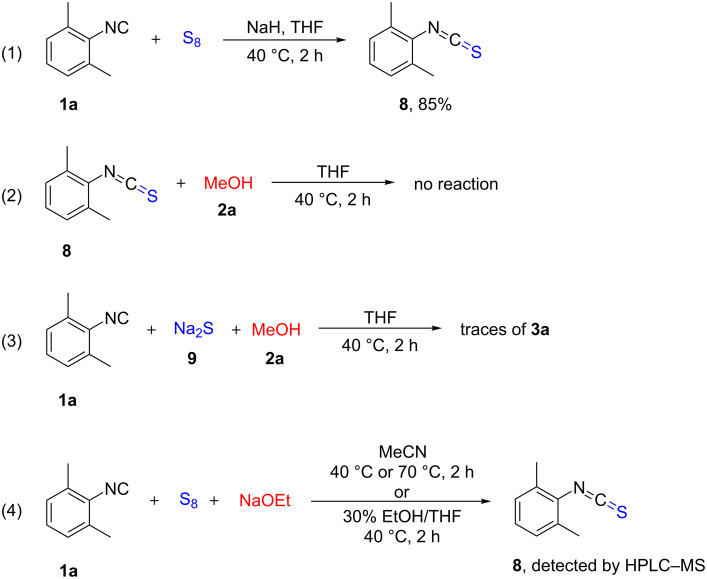
Control experiments.

As aforementioned, in some cases only isothiocyanate **8** was detected and/or isolated. Thus, in order to gain mechanistic insights on the generation of **3a**, we performed a series of experiments (Scheme 7). As shown in Table 1, isocyanide **1a** and sulfur did not react in the absence of a base (Table 1, entry 21). Therefore, the reaction was performed in the presence of NaH under the standard reaction conditions, providing **8** in 85% yield (Scheme 7, reaction 1). Notably, the analogous reaction reported by Tan and co-workers using potassium *tert*-butylate in *t*-BuOH/dioxane at 55 °C for 6 h resulted in the desired isothiocyanate in only 34% yield [69]. In the next step, we investigated the acylation of the alcohol component by the isothiocyanate (Scheme 7, reaction 2). In the absence of a base, no reaction occurred, and consequenctly the base was necessary for both steps of the thiocarbamate formation. This also explains why two equivalents of base were required. Sodium sulfide as the base provided only traces of **3a** suggesting that the activation of sulfur by NaH produces rather a polysulfide anion instead of sodium sulfide [74,107–108] (Scheme 7, reaction 3). It caught our attention that only isothiocyanate was generated in the presence of NaOEt (Table 1, entry 20). We suspected that THF might not be the best solvent for this base, hence the reaction was performed in MeCN providing exclusively **8** both at 40 °C and 70 °C, and the same result was obtained when a solvent mixture of ethanol and THF was used (Scheme 7, reaction 4).

**Scheme 8:**
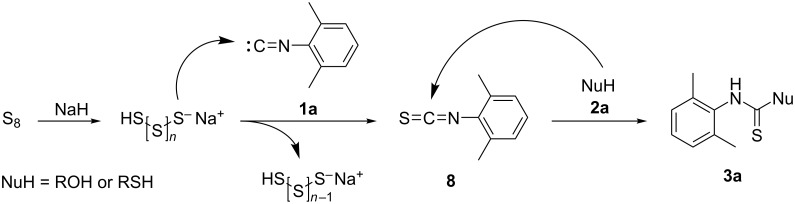
Proposed mechanism.

Based on the above experimental results and previous reports [74,107–108], a possible reaction mechanism has been proposed (Scheme 8). Initially, the reaction of elemental sulfur and NaH generates a polysulfide anion that is able to attack the carbenoid carbon atom of isocyanide **1a** yielding the isothiocyanate intermediate **8**. Then, the present nucleophile (NuH, alcohol or thiol) undergoes a nucleophilic addition on **8** providing thiocarbamate **3a**.

## Conclusion

In summary, we have developed an efficient, convenient and scalable multicomponent method for the synthesis of *O*-thiocarbamates and dithiocarbamates under mild reaction conditions. This approach includes an improved catalyst-free synthesis of isothiocyanates from elemental sulfur and isocyanides, and shows good functional group tolerance to halogen, olefin and nitrile groups among others. Moreover, this multicomponent reaction is suitable for a one-pot cascade annulation providing a thioxo dihydroquinazolinone derivative in a metal-free approach. Compared to other reported syntheses of thiocarbamates, this method is highlighted by its simplicity, atom economical nature and green operational method. Out of the 29 synthesized compounds, 18 new *O*-thiocarbamates, 5 new dithiocarbamates and 1 new thioxodihydroquinazolinone were characterized.

## Supporting Information

File 1 Experimental procedures, characterization data and copies of NMR spectra.
