# Supplementary material for: A novel three-component reaction between isocyanides, alcohols or thiols and elemental sulfur: a mild, catalyst-free approach towards O-thiocarbamates and dithiocarbamates
Source: Beilstein J Org Chem. 2019 Jul 10;15:1523–33. doi: 10.3762/bjoc.15.155 (PMC6633899; doi:10.3762/bjoc.15.155)
Supplement: File 1 — Experimental procedures, characterization data and copies of NMR spectra. [file Beilstein_J_Org_Chem-15-1523-s001.pdf]

BEILSTEIN JOURNAL OF ORGANIC CHEMISTRY

Supporting Information

for

A novel three-component reaction between isocyanides, alcohols or thiols and elemental sulfur: a mild, catalyst-free approach towards *O*-thiocarbamates and dithiocarbamates

András György Németh, György Miklós Keserű and Péter Ábrányi-Balogh

Beilstein J. Org. Chem. **2019,** *15,* 1523–1533. doi:10.3762/bjoc.15.155

Experimental procedures, characterization data and copies of NMR spectra

License and Terms: This is a supporting information file under the terms of the Creative Commons Attribution License (http://creativecommons.org/licenses/by/4.0). Please note that the reuse, redistribution and reproduction in particular requires that the authors and source are credited.
The license is subject to the *Beilstein Journal of Organic Chemistry* terms and conditions: (https://www.beilstein-journals.org/bjoc)

Table of contents

General information S2

Synthesis and experimental data S3

Table S1: Characteristic ^{1}H NMR chemical shifts and the ratio of the integrated area S16

^{1}H and APT NMR spectra S17

References S70

Experimental

General information

All melting points were determined on a Jasco SRS OptiMelt apparatus and are uncorrected. ^{1}H and ^{13}C NMR spectra were recorded in DMSO-d_6 or $CDCl_3$ solution at room temperature, on a Varian Unity Inova 500 spectrometer (500 and 125 MHz for ^{1}H and APT NMR spectra, respectively), with the residual solvent signal as the lock and TMS as the internal standard. Chemical shifts (δ) and coupling constants (*J*) are given in ppm and Hz, respectively. HPLC–MS measurements were performed using a Shimadzu LCMS-2020 device equipped with a Reprospher 100 C18 (5 µm; 100 × 3 mm) column and positive-negative double ion source (DUIS±) with a quadrupole MS analyzer in a range of 50–1000 *m*/*z*. The samples were eluted with gradient elution using eluent A (0.1% formic acid in water) and eluent B (0.1% formic acid in acetonitrile). Flow rate was set to 1.5 mL/min. The initial condition was 5% B eluent, followed by a linear gradient to 100% B eluent by 1.5 min, from 1.5 to 4.0 min 100% B eluent was retained; and from 4.0 to 4.5 min back to initial condition with 5% B eluent and retained to 5 min. The column temperature was kept at room temperature, and the injection volume was 1–10 µL. The purity of the compounds was assessed by HPLC with UV detection at 215 and 254 nm; all starting compounds are known, purchased or synthetically feasible and >95% pure.

The rotation around the N–CS bond of the *O*-thiocarbamates and dithiocarbamates is hindered and slow at room temperature compared to the time of the NMR measurement, therefore both *S*-*cis* and *S*-*trans* isomers were observed [1]. By the integration of characteristic signals of the ^{1}H NMR spectra, the ratio of the isomers is calculated and presented in (Table S1). In the experimental data, the mean of the rotamer signals and when possible, the individual chemical shifts are presented.

General procedure for the synthesis of thiocarbamates 3a–v:

Isocyanide (**1**, 1.0 mmol) was added to a mixture of sulfur (64 mg, 2.0 mmol), alcohol (**2**, 2.0 mmol) and sodium hydride (60% in paraffin oil, 80 mg, 2.0 mmol) in tetrahydrofuran (3 mL) under argon atmosphere, and the resulting mixture was stirred at 40 °C for 2 hours. Afterwards, the reaction mixture was diluted with ethyl acetate (30 mL), filtered through celite, and washed with brine (2 × 30 mL). Evaporation of the solvent, followed by the purification by flash column chromatography on silica gel provided the thiocarbamates **3a–v** in 30–94% yield.

Scaled-up procedure for the synthesis of thiocarbamate 3a:

The solution of 2,6-dimethylphenylisocyanide (**1a**, 2.62 g, 20.0 mmol) and **2a** (1.62 mL, 40.0 mmol) in tetrahydrofuran (30 mL) was added dropwise to a mixture of sulfur (1.28 g, 40.0 mmol), sodium hydride (60% in paraffin oil, 1.60 g, 40.0 mmol) in tetrahydrofuran (30 mL) under argon atmosphere at 0 °C and the resulting mixture was stirred at 40 °C for 2 hours. Afterwards, the reaction mixture was diluted with ethyl acetate (60 mL), filtered through celite, and washed with brine (2 × 60 mL), then the aqueous phase was extracted with ethyl acetate (60 mL). The organic phases were combined and after the evaporation of the solvent the crude product was recrystallized from ethyl acetate/hexane. Two additional crops were obtained from the recycled mother liquor. The combined fractions provided the thiocarbamate **3a** in 74% yield.

***O*-Methyl (2,6-dimethylphenyl)carbamothioate (3a):** White solid (184 mg, 94%), m.p. 65–66 °C (ethyl acetate); ^{1}H NMR: (500 MHz, $CDCl_3$) δ 8.16 (bs, NH), 7.47 (bs, NH), 7.18–7.07 (m, 3H), 4.04 (two signals: 4.08 (s), 4.00 (s), 3H) 2.26 (two signals:

2.27 (s), 2.25 (s), 6H) ppm; ^{13}C NMR: (125 MHz, $CDCl_3$) δ 190.97 (C), 190.94, (C), 136.50 (C), 135.66 (C), 134.79 (C), 134.26 (C), 128.32 (CH), 128.29 (CH), 128.17 (CH), 128.03 (CH), 58.70 (CH_3), 57.52 (CH_3), 18.19 (CH_3), 18.10 (CH_3) ppm; HRMS (ESI/Q-TOF) m/z: $[M+H]^+$ Calcd. for $C_{10}H_{14}NOS^+$: 196.0796; found: 196.0782.

***O*-Methyl (2-iodophenyl)carbamothioate (3b):** Dark green oil (276 mg, 94%); ^{1}H NMR: (500 MHz, $CDCl_3$) δ 8.08 (bs, 1H), 7.84 (dd, 1H, J_1 = 7.96 Hz, J_2 = 1.19 Hz), 7.75 (bs, NH), 7.36 (t, 1H, J = 7.99 Hz), 6.94 (t, 1H, J = 7.50 Hz), 3.95 (two signals: 4.10 (s), 3.80 (s), 3H) ppm; ^{13}C NMR: (125 MHz, $CDCl_3$) δ 189.68 (C), 139.23 (CH), 138.40 (C), 128.87 (CH), 127.73 (CH), 125.83 (CH), 121.44 (CH), 92.46 (C), 57.91 (CH_3) ppm; HRMS (ESI/Q-TOF) m/z: $[M+H]^+$ Calcd. for $C_8H_9NOSI^+$ 293.9449; found 293.9438.

***O*-Methyl (4-fluorophenyl)carbamothioate (3c):** Brown solid (120 mg, 65%). HRMS (ESI/Q-TOF) m/z: $[M+H]^+$ Calcd. for $C_8H_9NOF^+$ 186.0383; found 186.0380.

***O*-Methyl (4-methoxyphenyl)carbamothioate (3d):** Light brown solid (124 mg, 63%), m.p. 57–58 °C (ethyl acetate; lit. [2] 103 °C); ^{1}H NMR: (500 MHz, $CDCl_3$) δ 8.68 (bs, NH), 7.40 (bs, 1H), 7.15 (bs, 1H), 6.86 (d, 2H, J = 7.6 Hz), 4.09 (bs, 3H), 3.78 (s, 3H) ppm; HRMS (ESI/Q-TOF) m/z: $[M+H]^+$ Calcd. for $C_9H_{12}NO_2S^+$ 198.0588; found 198.0577.

***O*-Methyl cyclohexylcarbamothioate (3e):** Pale yellow solid (142 mg, 82%), m.p. 30–31 °C (ethyl acetate; lit. [3] 39–40 °C, pentane); ^{1}H NMR: (500 MHz, $CDCl_3$) δ 6.60 (bs, NH), 6.12 (bs, NH), 4.00 (two signals: 4.05 (s), 3.94 (s), 3H), 3.85 (two signals: 4.05

(m), 3.65 (m), 1H), multiple signals (2.07–2.03 (m), 1.92–1.88 (m), 1.73–1.57 (m), 1.42–1.13 (m), 10H) ppm; ^{13}C NMR: (125 MHz, $CDCl_3$) δ 189.85 (C), 189.75 (C), 58.15 (CH_3), 56.61 (CH_3), 53.87 (CH), 52.38 (CH), 32.62 (CH_2), 32.14 (CH_2), 25.42 (CH_2), 25.23 (CH_2), 24.68 (CH_2), 24.59 (CH_2) ppm; HRMS (ESI/Q-TOF) m/z: $[M+H]^+$ Calcd. for $C_8H_{16}NOS^+$ 174.0952; found 174.0940.

***O*-Methyl *tert*-butylcarbamothioate (3f):** White solid (80 mg, 54%), m.p. 43–44 °C (ethyl acetate; lit. [4] 51–53 °C); ^{1}H NMR: (500 MHz, $CDCl_3$) δ 6.73 (bs, NH), 6.20 (bs, NH), 3.97 (two signals: 4.07 (s), 3.87 (s), 3H), 1.47 (two signals: 1.61 (s), 1.32 (s), 6H) ppm; ^{13}C NMR: (125 MHz, $CDCl_3$) δ 191.36 (C), 58.19 (CH_3), 54.43 (C), 29.01 (CH_3), 28.09 (CH_3) ppm; HRMS (ESI/Q-TOF) m/z: $[M+H]^+$ Calcd. for $C_6H_{14}NOS^+$ 148.0796; found 148.0785.

***O*-Ethyl (2,6-dimethylphenyl)carbamothioate (3g):** Pale yellow solid (178 mg, 85%), m.p. 72–73 °C (ethyl acetate); ^{1}H NMR: (500 MHz, $CDCl_3$) δ 7.80 (bs, NH), 7.36 (bs, NH), 7.19–7.00 (m, 3H), 4.59–4.49 (m, 2H), 2.26 (two signals: 2.27 (s), 2.24 (s), 6H), 1.32 (two signals: 1.40 (t, J = 7.1 Hz), 1.23 (t, J = 7.1 Hz), 3H) ppm; ^{13}C NMR: (125 MHz, $CDCl_3$) δ 190.17 (C), 190.13 (C), 136.54 (C), 135.59 (C), 134.76 (C), 134.27 (C), 128.29 (CH), 128.25 (CH), 128.09 (CH), 127.90 (CH), 68.15 (CH_2), 66.82 (CH_2), 18.21 (CH_3), 18.11 (CH_3), 14.20 (CH_3), 14.15 (CH_3) ppm; HRMS (ESI/Q-TOF) m/z: $[M+H]^+$ Calcd. for $C_{11}H_{16}NOS^+$ 210.0952; found 210.0939.

***O*-Isopropyl (2,6-dimethylphenyl)carbamothioate (3h):** Pale yellow solid (116 mg, 52%), m.p. 73–74 °C (ethyl acetate); ^{1}H NMR: (500 MHz, $CDCl_3$) δ 7.76 (bs, NH), 7.30 (bs, NH), 7.17–7.05 (m, 3H), 5.65–5.53 (m, 2H), 2.26 (two signals: 2.27 (s), 2.24 (s),

6H), 1.30 (two signals: 1.39 (d, J = 6.2 Hz), 1.22 (d, J = 6.3 Hz), 6H) ppm; ^{13}C NMR: (125 MHz, $CDCl_3$) δ 189.54 (C), 189.32 (C), 136.56 (C), 135.69 (C), 134.86 (C), 134.34 (C), 128.26 (CH), 128.20 (CH), 127.97 (CH), 127.84 (CH), 76.10 (CH), 74.28 (CH), 21.71 (CH_3), 21.53 (CH_3), 18.19 (CH_3), 18.08 (CH_3) ppm; HRMS (ESI/Q-TOF) m/z: $[M+H]^+$ Calcd. for $C_{12}H_{18}NOS^+$ 224.1109; found 224.1098.

***O*-(*tert*-Butyl) (2,6-dimethylphenyl)carbamothioate (3i):** Pale yellow solid (107 mg, 45%), m.p. 83–84 °C (ethyl acetate); ^{1}H NMR: (500 MHz, $CDCl_3$) δ 7.58 (bs, NH), 7.24 (bs, NH), 7.17–7.04 (m, 3H), 2.25 (two signals: 2.26 (s), 2.24 (s), 6H), 1.63 (two signals: 1.71 (s), 1.54 (s), 9H) ppm; ^{13}C NMR: (125 MHz, $CDCl_3$) δ 188.17 (C), 187.92 (C), 136.67 (C), 135.50 (C), 134.90 (C), 134.84 (C), 128.17 (CH), 128.06 (CH), 127.84 (CH), 127.58 (CH), 86.40 (C), 85.28 (C), 28.40 (CH_3), 27.99 (CH_3), 18.24 (CH_3), 18.10 (CH_3) ppm; HRMS (ESI/Q-TOF) m/z: $[M+H]^+$ Calcd. for $C_{13}H_{20}NOS^+$ 238.1265; found 238.1253.

***O*-Allyl (2,6-dimethylphenyl)carbamothioate (3j):** Yellow solid (159 mg, 72%), m.p. 74–75 °C (ethyl acetate); ^{1}H NMR: (500 MHz, $CDCl_3$) δ 7.94 (bs, NH), 7.46 (bs, NH), 7.19–7.07 (m, 3H), 5.95 (two signals: 6.09–6.01 (m), 5.90–5.82 (m), 1H), 5.27 (two signals: 5.41 (d, J = 16.2 Hz), 5.12 (d, J = 17.2 Hz), 2H), 5.22 (two signals: 5.31 (d, J = 10.4 Hz), 5.13 (d, J = 10.5 Hz), 2H), 2.26 (two signals: 2.27 (s), 2.24 (s), 6H) ppm; ^{13}C NMR: (125 MHz, $CDCl_3$) δ 189.74 (C), 136.49 (C), 135.58 (C), 134.76 (C), 134.26 (C), 132.14 (CH), 131.53 (CH), 128.32 (CH), 128.30 (CH), 128.12 (CH), 127.97 (CH), 118.60 (CH_2), 118.22 (CH_2), 72.19 (CH_2), 71.19 (CH_2), 18.36 (CH_3), 18.23 (CH_3) ppm; HRMS (ESI/Q-TOF) m/z: $[M+H]^+$ Calcd. for $C_{12}H_{16}NOS^+$ 222.0952; found 222.0944.

***O*-(2-Hydroxyethyl) (2,6-dimethylphenyl)carbamothioate (3k):** Light yellow oil (136 mg, 34%); ^{1}H NMR: (500 MHz, $CDCl_3$) δ 10.47 (bs, NH), 10.37 (bs, NH), 7.10–7.03 (m, 3H), 4.40 (two signals: 4.42 (t, J = 4.8 Hz), 4.37 (t, J = 4.9 Hz), 2H), 3.59 (two signals: 3.70 (t, J = 4.9 Hz), 3.48 (t, J = 5.0 Hz), 2H), 2.13 (two signals: 2.14 (s), 2.12 (s), 6H) ppm; ^{13}C NMR: (125 MHz, $CDCl_3$) δ 190.04 (C), 188.78 (C), 136.45 (C), 136.11 (C), 135.67 (C), 135.40 (C), 128.28 (CH), 128.19 (CH), 127.62 (CH), 127.55 (CH), 72.53 (CH_2), 72.09 (CH_2), 59.56 (CH_2), 59.29 (CH_2), 18.21 (CH_3), 18.15 (CH_3); HRMS (ESI/Q-TOF) m/z: $[M+H]^+$ Calcd. for $C_{11}H_{16}NO2S^+$ 226.0901; found 226.0893.

***O*-Benzyl (2,6-dimethylphenyl)carbamothioate (3l):** White solid (201 mg, 74%), m.p. 69–70 °C (ethyl acetate); ^{1}H NMR: (500 MHz, $CDCl_3$) δ 7.81 (bs, NH), multiple signals: (7.47–7.37 (m), 7.28–7.05 (m), 8H) 5.57 (two signals: 5.58 (s), 5.55 (s), 2H), 2.24 (two signals: 2.28 (s), 2.20 (s), 6H) ppm; ^{13}C NMR: (125 MHz, $CDCl_3$) δ 190.07 (C), 189.88 (C), 136.49 (C), 135.75 (C), 135.66 (C), 135.44 (C), 134.72 (C), 134.18 (C), 128.82 (CH), 128.61 (CH), 128.45 (CH), 128.35 (CH), 128.34 (CH), 128.13 (CH), 128.07 (CH), 127.59 (CH), 73.17 (CH_2), 72.43 (CH_2), 18.21 (CH_3), 18.12 (CH_3) ppm; HRMS (ESI/Q-TOF) m/z: $[M+H]^+$ Calcd. for $C_{16}H_{18}NOS^+$ 272.1109; found 272.1099.

***O*-(4-Chlorobenzyl) (2,6-dimethylphenyl)carbamothioate (3m):** White solid (272 mg, 89%), m.p. 96–97 °C (ethyl acetate); ^{1}H NMR: (500 MHz, $CDCl_3$) δ 7.87 (bs, NH), 7.48 (bs, NH), 7.40–7.36 (m, 2H), 7.25–7.05 (m, 5H), 5.53 (two signals: 5.55 (s), 5.50 (s), 2H), 2.23 (two signals: 2.27 (s), 2.19 (s), 6H) ppm; ^{13}C NMR: (125 MHz, $CDCl_3$) δ 189.66 (C), 136.43 (C), 135.60 (C), 134.75 (C), 134.35 (C), 134.28 (C), 134.21 (C), 134.00 (C), 133.99 (C), 129.64 (CH), 129.09 (CH), 128.79 (CH), 128.59 (CH), 128.39 (CH), 128.18 (CH), 128.15 (CH), 72.08 (CH_2), 71.35 (CH_2), 18.24 (CH_3), 18.15 (CH_3)

ppm; HRMS (ESI/Q-TOF) m/z: $[M+H]^+$ Calcd. for $C_{16}H_{17}NOSCl^+$ 306.0719; found 306.0710.

***O*-(4-Bromobenzyl) (2,6-dimethylphenyl)carbamothioate (3n):** White solid (284 mg, 81%), m.p. 91–92 °C (ethyl acetate); ^{1}H NMR: (500 MHz, $CDCl_3$) δ 8.02 (bs, NH), 7.53 (d, 1H, *J* = 8.3 Hz), 7.40 (d, 1H, *J* = 8.4 Hz), 7.33 (d, 1H, *J* = 8.3 Hz), 7.14–7.03 (m, 4H), 5.51 (two signals: 5.53 (s), 5.48 (s), 2H), 2.23 (two signals: 2.27 (s), 2.19 (s), 6H) ppm; ^{13}C NMR: (125 MHz, $CDCl_3$) δ 185.01 (C), 184.89 (C), 131.67 (C), 130.86 (C), 130.09 (C), 129.91 (C), 129.72 (C), 129.38 (C), 127.00 (CH), 126.80 (CH), 125.17 (CH), 124.58 (CH), 123.66 (CH), 123.63 (CH), 123.43 (CH), 117.70 (C), 117.40 (C), 67.40 (CH_2), 66.61 (CH_2), 13.48 (CH_3), 13.37 (CH_3) ppm; HRMS (ESI/Q-TOF) m/z: $[M+H]^+$ Calcd. for $C_{16}H_{17}NOSBr^+$ 350.0214; found 350.0202.

***O*-(4-Methoxybenzyl) (2,6-dimethylphenyl)carbamothioate (3o):** White solid (160 mg, 53%), m.p. 107–108 °C (ethyl acetate); ^{1}H NMR: (500 MHz, DMSO-*d*$_6$) δ 9.55 (bs, NH), 9.29 (bs, NH), 7.25–7.05 (m, 5H), 6.86–6.76 (m, 2H), 4.00 (two signals: 4.06 (bs), 3.93 (bs), 2H), 3.70 (two signals: 3.71 (bs), 3.69 (bs), 3H), 2.14 (two signals: 2.15 (bs), 2.12 (bs), 6H) ppm; ^{13}C NMR: (125 MHz, DMSO-*d*$_6$) δ 191.30 (C), 173.13 (C), 169.74 (C), 164.99 (C), 158.70 (C), 130.16 (CH), 128.19 (CH), 128.16 (CH), 127.22 (CH), 114.28 (CH), 55.50 (CH_3), 32.84 (CH_2), 18.43 (CH_3) ppm; HRMS (ESI/Q-TOF) m/z: $[M+H]^+$ Calcd. for $C_{17}H_{20}NO_2S^+$ 302.1214; found 302.1204.

***O*-(4-Cyanobenzyl) (2,6-dimethylphenyl)carbamothioate (3p):** Pale yellow solid (184 mg, 62%), m.p. 132–133 °C (ethyl acetate); ^{1}H NMR: (500 MHz, DMSO-*d*$_6$) δ 10.75 (bs, NH), 10.66 (bs, NH), multiple signals: (7.86 (d, *J* = 8.18 Hz), 7.74 (d, *J* =

8.23 Hz), 7.59 (d, J = 8.58 Hz), 7.30 (d, J = 8.18 Hz), 7.13–7.05 (m), 7H), 5.60 (two signals: 5.64 (s), 5.55 (s), 2H), 2.13 (two signals: 2.15 (s), 2.11 (s), 6H) ppm; ^{13}C NMR: (125 MHz, DMSO-d_6) δ 189.42 (C), 188.26 (C), 142.77 (C), 142.20 (C), 136.25 (C), 135.97 (C), 135.57 (C), 135.29 (C), 132.86 (CH), 132.72 (C), 128.96 (CH), 128.51 (CH), 128.44 (CH), 128.38 (CH), 128.32 (CH), 128.26 (CH), 127.87 (CH), 127.82 (CH), 119.13 (C), 118.99 (C), 111.07 (C), 70.51 (CH_2), 69.92 (CH_2), 18.21 (CH_3), 18.09 (CH_3) ppm; HRMS (ESI/Q-TOF) m/z: $[M+H]^+$ Calcd. for $C_{17}H_{16}N_2OS^+$ 297.1056; found 297.1052.

***O*-(4-Nitrobenzyl) (2,6-dimethylphenyl)carbamothioate (3q).** Yellow solid (95 mg, 30%), m.p. 133–134 °C (ethyl acetate); ^{1}H NMR: (500 MHz, $CDCl_3$) δ multiple signals: (8.25 (d, J = 8.62 Hz), 8.12 (d, J = 8.65 Hz), 7.95 (bs, NH), 7.60 (d, 1H, J = 8.60 Hz), 7.27 (d, 1H, J = 8.59 Hz), 7.21–7.09 (m), 8H), 5.66 (two signals: 5.70 (s), 5.62 (s), 2H), 2.25 (two signals: 2.27 (s), 2.23 (s), 6H)) ppm; ^{13}C NMR: (125 MHz, $CDCl_3$) δ 189.49 (C), 189.29 (C), 143.29 (C), 142.74 (C), 136.32 (C), 135.59 (C), 134.49 (C), 133.99 (C), 128.53 (CH), 128.44 (CH), 128.40 (CH), 128.29 (CH), 128.26 (CH), 127.71 (CH), 123.79 (CH), 123.66 (CH), 71.21 (CH_2), 70.27 (CH_2), 18.21 (CH_3), 18.07 (CH_3) ppm; HRMS (ESI/Q-TOF) m/z: $[M+H]^+$ Calcd. for $C_{16}H_{17}N_2O_3S^+$ 317.0959; found 317.0944.

***O*-(2-Chlorobenzyl) (2,6-dimethylphenyl)carbamothioate (3r):** White solid (254 mg, 83%), m.p. 99–100 °C (ethyl acetate); ^{1}H NMR: (500 MHz, $CDCl_3$) δ 8.12 (bs, NH), 7.55 (bs, NH), multiple signals: (7.53–7.51 (m), 7.44–7.42 (m), 7.32–7.30 (m), 7.21–7.05 (m), 7H), 5.65 (two signals: 5.68 (s), 5.61 (s), 2H), 2.27 (two signals: 2.29 (s), 2.24 (s), 6H) ppm; ^{13}C NMR: (125 MHz, $CDCl_3$) δ 189.71 (C), 189.64 (C), 136.49 (C), 135.72 (C), 134.71 (C), 134.18 (C), 134.03 (C), 133.60 (C), 133.26 (C), 133.02 (C), 130.18

(CH), 129.76 (CH), 129.70 (CH), 129.34 (CH), 129.22 (CH), 128.87 (CH), 128.37 (CH), 128.16 (CH), 126.90 (CH), 126.69 (CH), 70.39 (CH_2), 69.65 (CH_2), 18.24 (CH_3), 18.13 (CH_3) ppm; HRMS (ESI/Q-TOF) m/z: $[M+H]^+$ Calcd. for $C_{16}H_{17}NOSCl^+$ 306.0719; found 306.0706.

***O*-(2-Iodobenzyl) (2,6-dimethylphenyl)carbamothioate (3s):** White solid (322 mg, 81%), m.p. 69–70 °C (ethyl acetate); ^{1}H NMR: (500 MHz, $CDCl_3$) δ 8.02 (bs, NH), multiple signals: (7.91 (d, J = 7.86 Hz), 7.77 (d, J = 7.82 Hz), 7.54 (bs, NH), 7.47 (d, J = 7.32 Hz), 7.39 (t, J = 7.42 Hz), 7.22–6.93 (m), 7H), 5.53 (two signals: 5.56 (s), 5.50 (s), 2H), 2.27 (two signals: 2.30 (s), 2.24 (s), 6H) ppm; ^{13}C NMR: (125 MHz, $CDCl_3$) δ 189.52 (C), 189.45 (C), 139.68 (CH), 139.26 (CH), 138.33 (C), 137.91 (C), 136.49 (C), 135.73 (C), 134.69 (C), 134.22 (C), 130.15 (CH), 130.03 (CH), 129.59 (CH), 128.68 (CH), 128.38 (CH), 128.19 (CH), 128.15 (CH), 128.12 (CH), 98.83 (C), 97.37 (C), 76.63 (CH_2), 75.78 (CH_2), 18.36 (CH_3), 18.21 (CH_3) ppm; HRMS (ESI/Q-TOF) m/z: $[M+H]^+$ Calcd. for $C_{16}H_{17}NOSI^+$ 398.0075; found 398.0060.

***O*-(2-Methoxybenzyl) (2,6-dimethylphenyl)carbamothioate (3t):** Yellow oil (169 mg, 56%); ^{1}H NMR: (500 MHz, $CDCl_3$) δ 8.43 (bs, NH), 7.65 (bs, NH), multiple signals: (7.45 (d, J = 7.44 Hz), 7.36 (td, J_1 = 8.32 Hz, J_2 = 1.41 Hz), 7.25 (td, J_1 = 8.37 Hz, J_2 = 1.39 Hz), 7.21–7.12 (m), 7.08-7.00 (m), 6.93 (d, J = 8.24 Hz), 6.85–6.81 (m), 7H), 5.63 (two signals: 5.67 (s), 5.58 (s), 2H), 3.80 (two signals: 3.87 (s), 3.73 (s), 3H), 2.29 (two signals: 2.32 (s), 2.26 (s), 3H) ppm; ^{13}C NMR: (125 MHz, $CDCl_3$) δ 190.20 (C), 190.01 (C), 157.70 (C), 157.17 (C), 136.57 (C), 135.74 (C), 135.01 (C), 134.46 (C), 129.86 (CH), 129.33 (CH), 128.71 (CH), 128.35 (CH), 128.26 (CH), 128.06 (CH), 127.94 (CH), 124.18 (C), 123.92 (C), 120.50 (CH), 120.28 (CH), 110.65 (CH), 110.26 (CH), 69.22

(CH_2), 68.05 (CH_2), 55.50 (CH_3), 55.22 (CH_3), 18.23 (CH_3), 18.18 (CH_3) ppm; HRMS (ESI/Q-TOF) m/z: $[M+H]^+$ Calcd. for $C_{17}H_{20}NO_2S^+$ 302.1214; found 302.1197.

***O*-(3,4,5-Trimethoxybenzyl) (2,6-dimethylphenyl)carbamothioate (3u):** White solid (123 mg, 34%), m.p. 133–134 °C (ethyl acetate); ^{1}H NMR: (500 MHz, DMSO-d_6) δ 10.66 (bs, NH), 10.53 (bs, NH), 7.12–7.07 (m, 3H), 6.77 (s, 1H), 6.40 (s, 1H), 5.41 (two signals: 5.47 (s), 5.35 (s), 2H), 3.77 (s, 3H), multiple signals: (3.77 (s), 3.65 (s), 3.61 (s), 3.58 (s), 9H), 2.13 (two signals: 2.14 (s), 2.12 (s), 6H) ppm; ^{13}C NMR: (125 MHz, DMSO-d_6) δ 189.78 (C), 188.36 (C), 153.39 (C), 153.19 (C), 137.78 (C), 137.33 (C), 136.40 (C), 136.01 (C), 135.79 (C), 135.40 (C), 132.47 (C), 131.89 (C), 128.35 (CH), 128.27 (CH), 127.77 (CH), 127.74 (CH), 105.69 (CH), 104.48 (CH), 71.69 (CH_2), 71.28 (CH_2), 60.49 (CH_3), 60.39 (CH_3), 56.35 (CH_3), 56.12 (CH_3), 18.23 (CH_3), 18.13 (CH_3) ppm; HRMS (ESI/Q-TOF) m/z: $[M+H]^+$ Calcd. for $C_{19}H_{24}NO_4S^+$ 362.1426; found 362.1422.

General procedure for the synthesis of dithiocarbamates 5a–e:

2,6-Dimethylphenylisocyanide (**1a**, 131 mg, 1.0 mmol) was added to a mixture of sulfur (38 mg, 1.2 mmol), thiol (**4**, 2.0 mmol) and sodium hydroxide (80 mg, 2.0 mmol) in *N*,*N*-dimethylacetamide (3 mL) under argon atmosphere and the resulting mixture was stirred at 70 °C and monitored by TLC. Afterward completion, the reaction mixture was diluted with ethyl acetate (30 mL), and washed with brine (2 × 30 mL). The aqueous phase was washed with ethyl acetate (15 mL), and the organic layers were combined. Evaporation of the solvent followed by purification by flash column chromatography on silica gel provided the dithiocarbamate **5a–e** in 22–65% yield.

Benzyl (2,6-dimethylphenyl)carbamodithioate (5a): Off-white solid (170 mg, 59%), m.p. 78–79 °C (ethyl acetate); ^{1}H NMR: (500 MHz, $CDCl_3$) δ 8.95 (bs, NH), 8.05 (bs, NH), 7.43–7.09 (m, 8H), 4.57 (two signals: 4.62 (s), 4.51 (s), 2H), 2.26 (two signals: 2.29 (s), 2.23 (s), 6H) ppm; ^{13}C NMR: (125 MHz, $CDCl_3$) δ 202.81 (C), 197.92 (C), 136.93 (C), 136.63 (C), 136.37 (C), 136.11 (C), 135.98 (C), 135.21 (C), 129.31 (CH), 129.22 (CH), 129.03 (CH), 128.71 (CH), 128.57 (CH), 128.50 (CH), 128.32 (CH), 127.61 (CH), 127.45 (CH), 41.12 (CH_2), 40.71 (CH_2), 18.11 (CH_3), 18.07 (CH_3) ppm; HRMS (ESI/Q-TOF) m/z: $[M+H]^+$ Calcd. for $C_{16}H_{18}NS_2^+$ 288.0880; found 288.0871.

Pentyl (2,6-dimethylphenyl)carbamodithioate (5b): White solid (175 mg, 65%), m.p. 60–61 °C (ethyl acetate); ^{1}H NMR: (500 MHz, $CDCl_3$) δ 8.94 (bs, NH), 8.04 (bs, NH), 7.24–7.09 (m, 3H), 3.27 (two signals: 3.33 (t, J = 7.38 Hz), 3.21 (t, J = 7.45 Hz), 2H), 2.27 (two signals: 2.29 (s), 2.25 (s), 6H), 1.77–1.61 (m, 2H), 1.46–1.26 (m, 4H), 0.90 (two signals: 0.92 (t, J = 7.22 Hz), 0.87 (t, J = 7.01 Hz), 3H) ppm; ^{13}C NMR: (125 MHz, $CDCl_3$) δ 203.68 (C), 136.96 (C), 136.19 (C), 135.16 (C), 129.20 (CH), 128.49 (CH), 128.37 (CH), 128.25 (CH), 36.28 (CH_2), 36.17 (CH_2), 31.06 (CH_2), 31.00 (CH_2), 28.73 (CH_2), 28.29 (CH_2), 22.25 (CH_2), 22.19 (CH_2), 18.05 (CH_3), 13.92 (CH_3), 13.87 (CH_3) ppm; HRMS (ESI/Q-TOF) m/z: $[M+H]^+$ Calcd. for $C_{14}H_{22}NS_2^+$ 268.1188; found 268.1188.

Isopropyl (2,6-dimethylphenyl)carbamodithioate (5c): Off-white solid (122 mg, 51%), m.p. 54–55 °C (ethyl acetate); ^{1}H NMR: (500 MHz, $CDCl_3$) δ 8.93 (bs, NH), 8.03 (bs, NH), 7.24–7.09 (m, 3H), 4.07–3.98 (m, 1H), 2.27 (two signals: 2.29 (s), 2.25 (s), 6H), 1.40 (two signals: 1.46 (d, J = 6.85 Hz), 1.35 (d, J = 6.89 Hz), 6 H) ppm; ^{13}C NMR: (125 MHz, $CDCl_3$) δ 202.98 (C), 198.25 (C), 136.84 (C), 136.21 (C), 135.39 (C), 129.17

(CH), 128.45 (CH), 128.24 (CH), 41.73 (CH), 41.60 (CH), 22.76 (CH_3), 22.63 (CH_3), 18.06 (CH_3) ppm; HRMS (ESI/Q-TOF) m/z: $[M+H]^+$ Calcd. for $C_{12}H_{18}NS_2^+$ 240.0875; found 240.0876.

Cyclohexyl (2,6-dimethylphenyl)carbamodithioate (5d): White solid (131 mg, 47%), m.p. 106–107 °C (ethyl acetate); ^{1}H NMR: (500 MHz, $CDCl_3$) δ 8.66 (bs, NH), 7.94 (bs, NH), 7.23–7.09 (m, 3H), 3.95–3.86 (m, 1H), 2.26 (two signals: 2.28 (s), 2.24 (s), 6H), multiple signals: (2.16–2.07 (m), 1.77–1.59 (m), 1.53–1.16 (m), 10 H) ppm; ^{13}C NMR: (125 MHz, $CDCl_3$) δ 202.97 (C), 136.88 (C), 136.22 (C), 135.38 (C), 129.16 (CH), 128.45 (CH), 128.36 (CH), 128.23 (CH), 49.52 (CH), 49.46 (CH), 32.90 (CH_2), 32.72 (CH_2), 26.13 (CH_2), 25.52 (CH_2), 18.06 (CH_3) ppm; HRMS (ESI/Q-TOF) m/z: $[M+H]^+$ Calcd. for $C_{15}H_{22}NS_2^+$ 280.1188; found 280.1186.

***tert*-Butyl (2,6-dimethylphenyl)carbamodithioate (5e):** White solid (55 mg, 22%), m.p. 97–98 °C (ethyl acetate); ^{1}H NMR: (500 MHz, $CDCl_3$) δ 8.51 (bs, NH), 8.12 (bs, NH), 7.21–7.09 (m, 3H), 2.28 (two signals: 2.29 (s), 2.26 (s), 6H), 1.64 (two signals: 1.67 (s), 1.60 (s), 9H) ppm; ^{13}C NMR: (125 MHz, $CDCl_3$) δ 202.43 (C), 197.09 (C), 136.66 (C), 136.21 (C), 135.92 (C), 135.64 (C), 128.96 (CH), 128.39 (CH), 128.37 (CH), 128.25 (CH), 50.79 (C), 50.55 (C), 30.05 (CH_3), 29.48 (CH_3), 18.15 (CH_3), 18.09 (CH_3) ppm; HRMS (ESI/Q-TOF) m/z: $[M+H]^+$ Calcd. for $C_{13}H_{20}NS_2^+$ 254.1032; found 254.1033.

Synthesis of 3-(2,6-dimethylphenyl)-2-thioxo-2,3-dihydroquinazolin-4(1*H*)-one (7):

2,6-Dimethylphenylisocyanide (**1a**, 131 mg, 1.0 mmol) was added to a mixture of sulfur (38 mg, 1.2 mmol), methyl 2-aminobenzoate (**6**, 259 µL, 2.0 mmol) and sodium hydroxide (80 mg, 2.0 mmol) in dimethyl sulfoxide (3 mL) under argon atmosphere, and the resulting mixture was stirred at 85 °C for 1.5 hours. Afterwards, the reaction mixture was diluted with ethyl acetate (30 mL), and washed with brine (2 × 30 mL). The aqueous phase was washed with ethyl acetate (15 mL), and the organic layers were combined. Evaporation of the solvent followed by purification by flash column chromatography on silica gel provided **7** in 40% yield (113 mg).

3-(2,6-Dimethylphenyl)-2-thioxo-2,3-dihydroquinazolin-4(1*H*)-one (7): White solid (113 mg, 40%), m.p. 168–169 °C (ethyl acetate); ^{1}H NMR: (500 MHz, DMSO-d_6) δ 13.14 (bs, NH), 7.97 (d, J = 7.81 Hz, 1H), 7.79 (t, J = 8.13 Hz, 1H), 7.46 (d, J = 8.26 Hz, 1H), 7.35 (t, J = 7.58 Hz, 1H), 7.21–7.14 (m, 3H), 1.99 (s, 6H) ppm; ^{13}C NMR: (125 MHz, DMSO-d_6) δ 174.98 (C), 159.34 (C), 140.17 (C), 137.53 (C), 136.45 (CH), 135.30 (C), 128.68 (CH), 128.67 (CH), 128.01 (CH), 125.12 (CH), 116.32 (CH), 115.81 (C), 17.63 (CH_3) ppm; HRMS (ESI/Q-TOF) m/z: $[M+H]^+$ Calcd. for $C_{16}H_{14}N_2OS^+$ 283.0905; found 283.0910.

Synthesis of 2-isothiocyanato-1,3-dimethylbenzene (8):

2,6-Dimethylphenylisocyanide (**1a**, 1.0 mmol) was added to a mixture of sulfur (64 mg, 2.0 mmol) and sodium hydride (60% in paraffin oil, 80 mg, 2.0 mmol) in tetrahydrofuran (3 mL) under argon atmosphere, and the resulting mixture was stirred at 40 °C for 2 hours. Afterwards, the reaction mixture was diluted with ethyl acetate (30 mL), filtered

through celite, and washed with brine (2 × 30 mL). Evaporation of the solvent, followed by purification by flash column chromatography on silica gel provided the isothiocyanate **8** in 85% yield.

2-Isothiocyanato-1,3-dimethylbenzene (8) [5]**:** Yellow oil (139 mg; 85%); ^{1}H NMR: (500 MHz, $CDCl_3$) δ 7.09–7.03 (m, 3H), 2.37 (s, 6H) ppm.

Table S1: Characteristic ^{1}H NMR chemical shifts and the ratio of the integrated area.

Entry	Signal	mean	upper signal (area%)	lower signal (area%)
**3a**	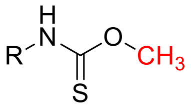	4.04	4.08 (36)	4.00 (64)
**3b**		3.95	4.10 (98)	3.85 (2)
**3d**		4.09	no rotamers observed	
**3e**		3.97	4.07 (87)	3.87 (13)
**3f**		3.85	4.05 (40)	3.65 (60)
**3g**	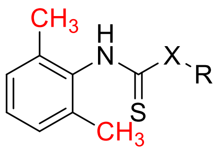 X = O,S	2.26	2.27 (41)	2.24 (59)
**3h**		2.26	2.27 (40)	2.24 (60)
**3i**		2.25	2.26 (37)	2.24 (63)
**3j**		2.26	2.27 (42)	2.24 (58)
**3k**		2.13	2.14 (13)	2.12 (87)
**3l**		2.24	2.28 (49)	2.20 (51)
**3m**		2.23	2.27 (46)	2.19 (54)
**3n**		2.23	2.27 (45)	2.19 (55)
**3o**		4.00	4.06 (75)	3.93 (25)
**3p**[a]		2.13	2.15 (66)	2.11 (34)
**3q**		2.25	2.27 (45)	2.23 (55)
**3r**		2.27	2.29 (49)	2.24 (51)
**3s**		2.27	2.30 (53)	2.24 (47)
**3t**		2.29	2.32 (44)	2.26 (56)
**3u**		2.13	2.14 (56)	2.12 (44)
**5a**		2.26	2.29 (82)	2.23 (18)
**5b**		2.27	2.29 (82)	2.25 (18)
**5c**		2.27	2.29 (82)	2.25 (18)
**5d**		2.26	2.28 (81)	2.24 (19)
**5e**		2.28	2.29 (62)	2.26 (38)

[a]Calculated from the benzylic protons.

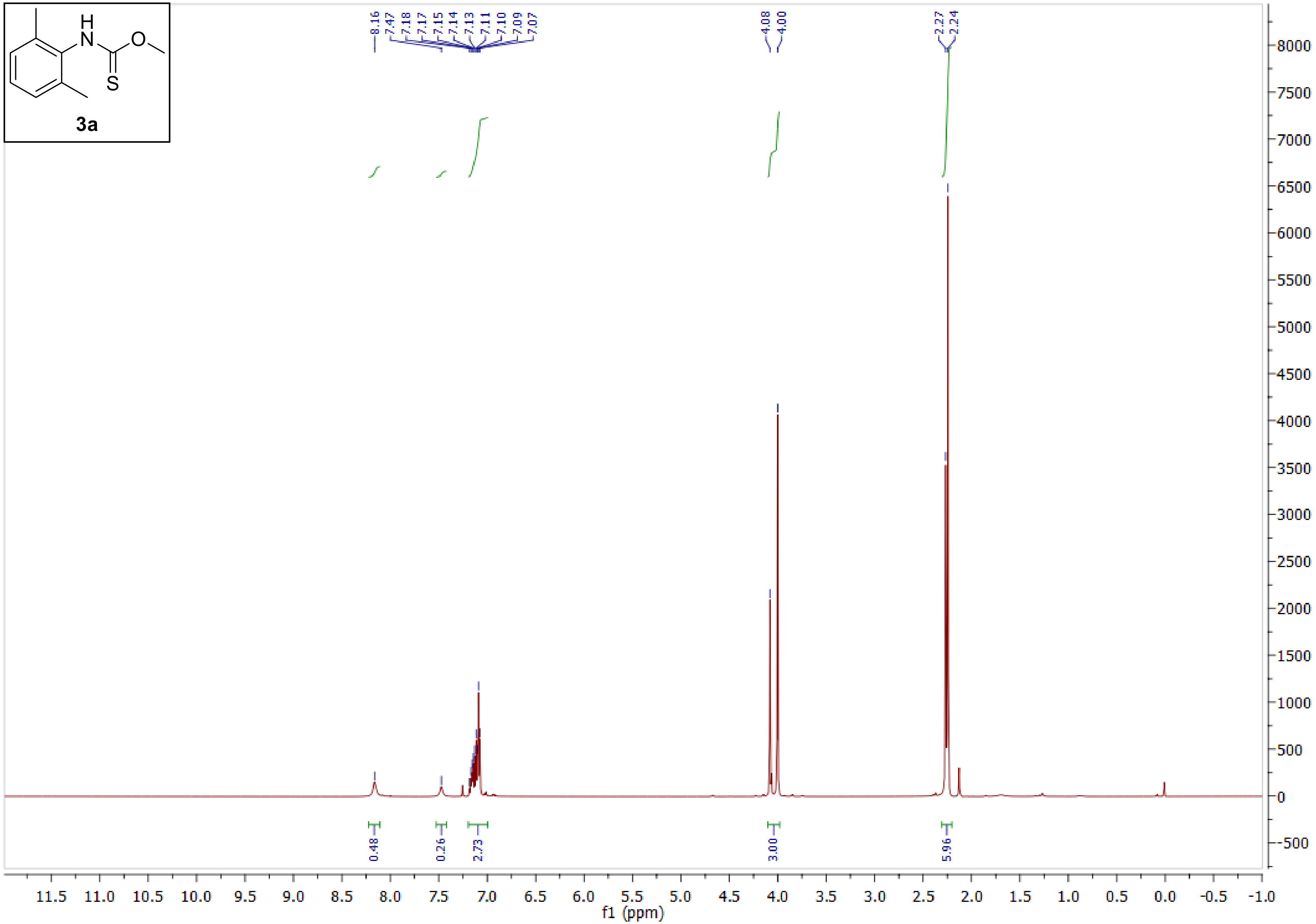
3a
8.16
7.47
7.18
7.17
7.15
7.14
7.13
7.11
7.10
7.09
7.07
4.08
4.00
2.27
2.24
0.48
0.26
2.73
3.00
5.96
f1 (ppm)

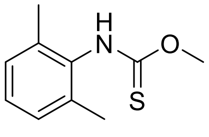

3a

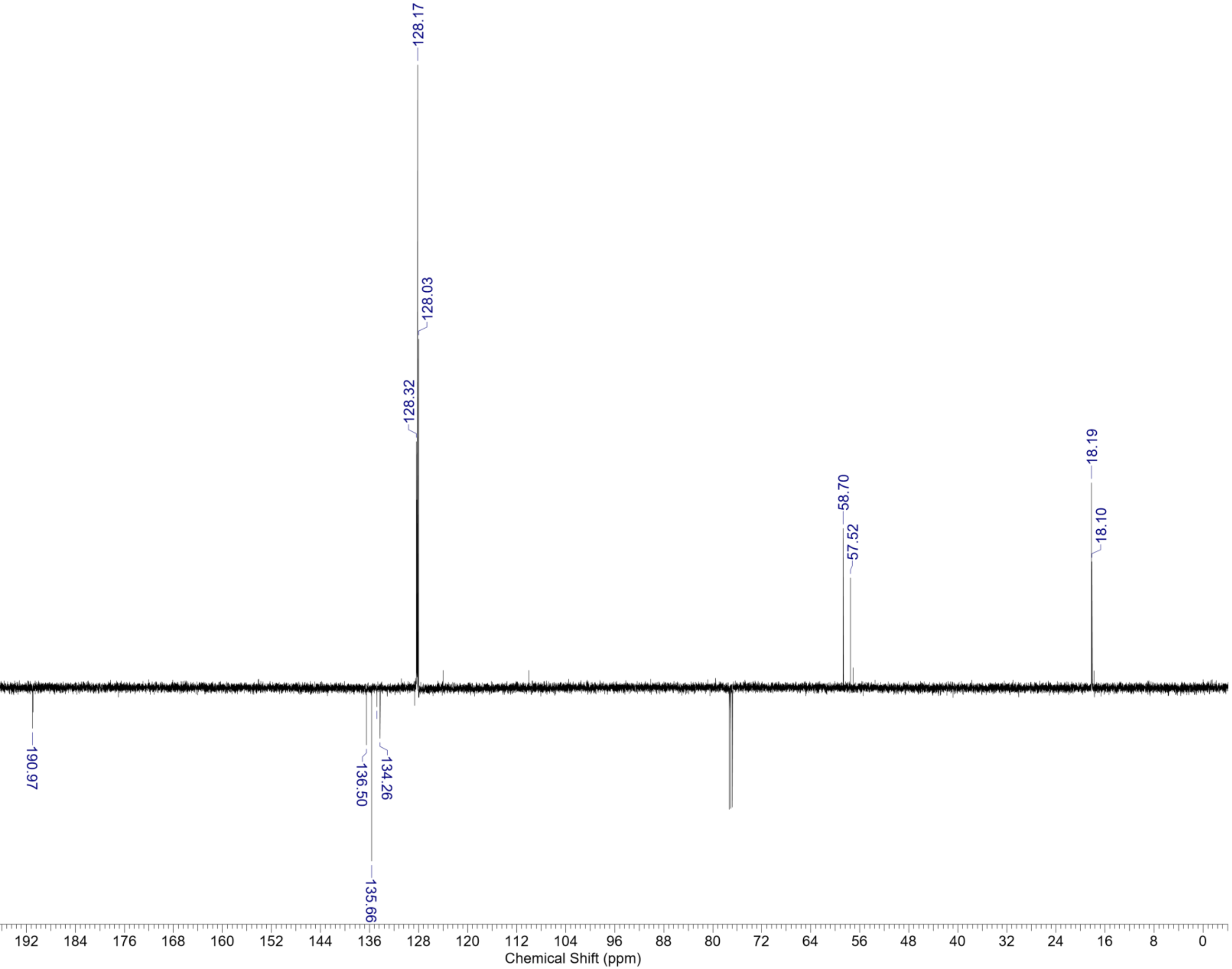
128.17
128.03
128.32
58.70
57.52
18.19
18.10
190.97
136.50
134.26
135.66
208 200 192 184 176 168 160 152 144 136 128 120 112 104 96 88 80 72 64 56 48 40 32 24 16 8 0
Chemical Shift (ppm)

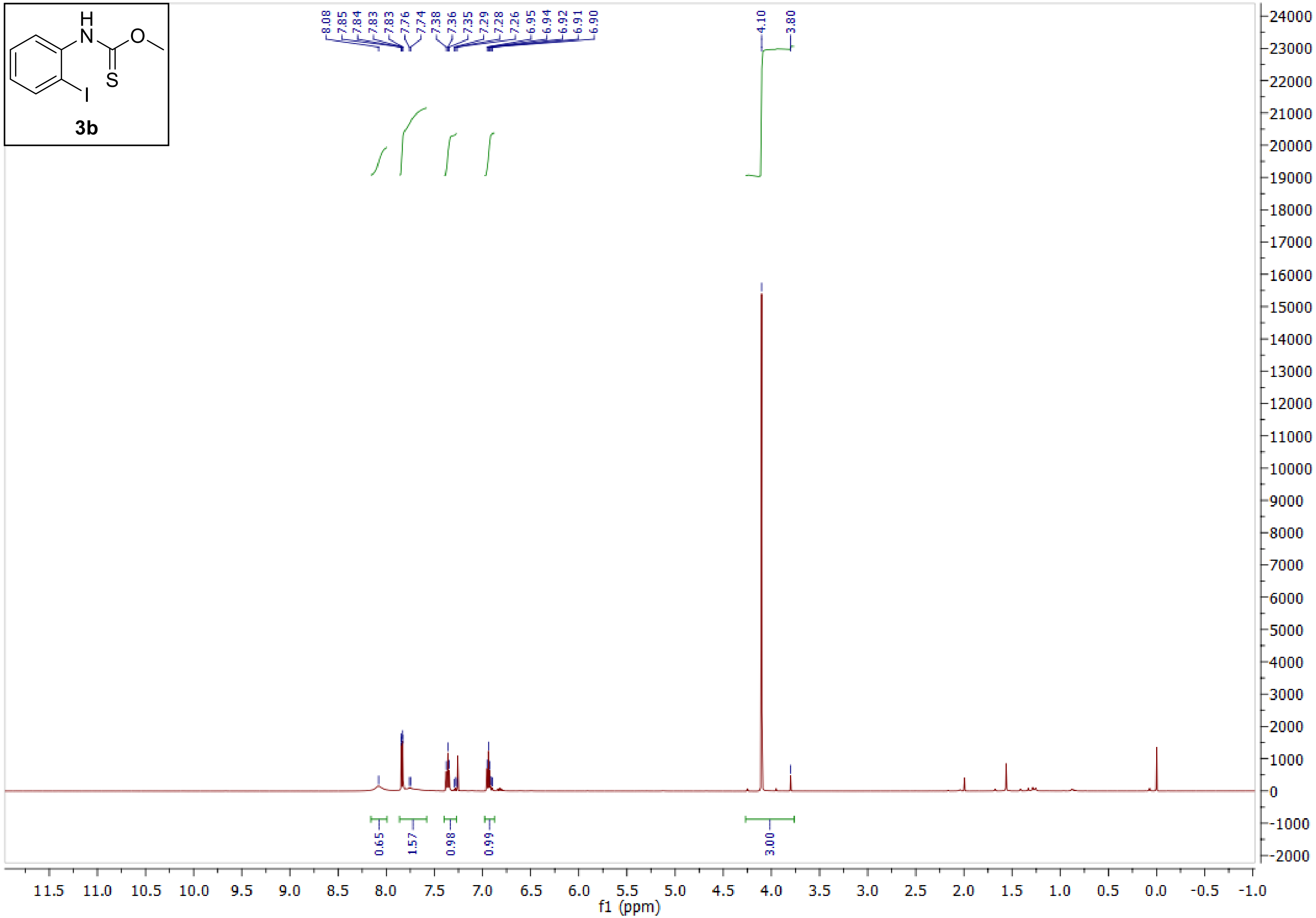
3b
8.08
7.85
7.84
7.83
7.83
7.76
7.74
7.38
7.36
7.35
7.29
7.28
7.26
6.95
6.94
6.92
6.91
6.90
4.10
3.80
0.65
1.57
0.98
0.99
3.00
f1 (ppm)

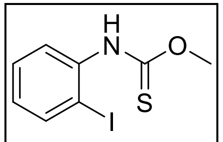

H
N
O
S
I

3b

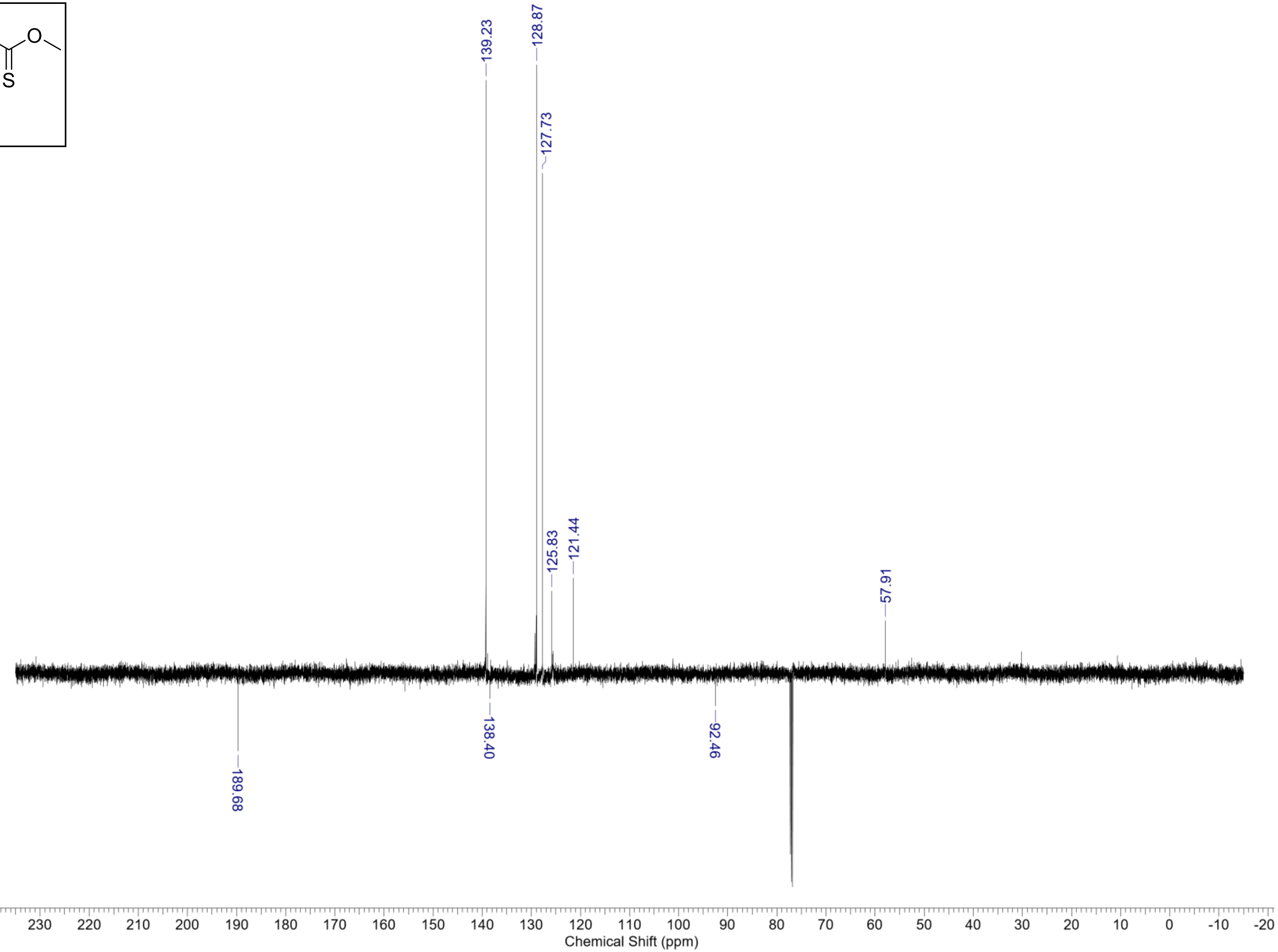

139.23
128.87
127.73
125.83
121.44
57.91
189.68
138.40
92.46
230 220 210 200 190 180 170 160 150 140 130 120 110 100 90 80 70 60 50 40 30 20 10 0 -10 -20
Chemical Shift (ppm)

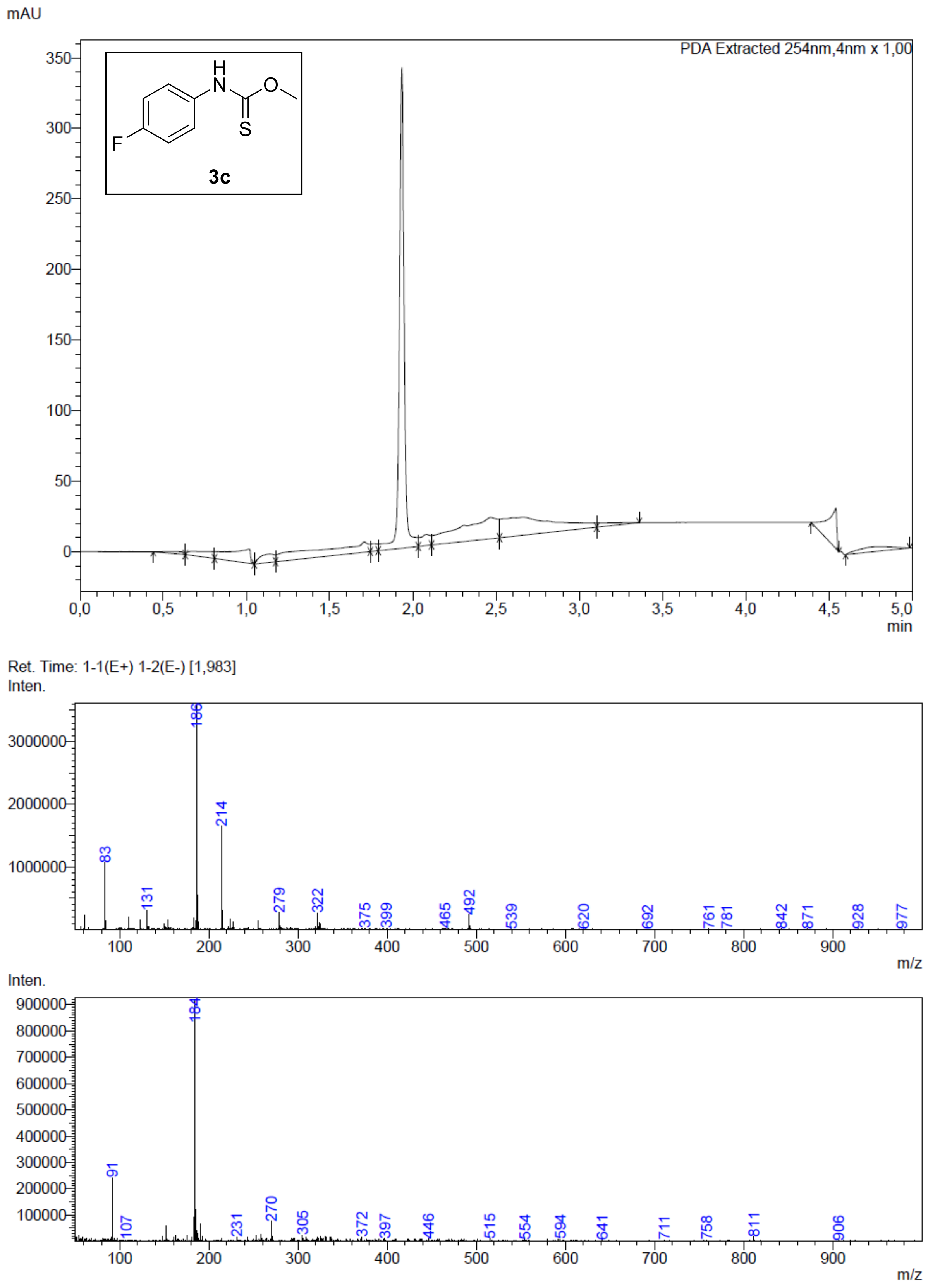

Figure S1: HPLC–MS spectrum of the reaction mixture of the preparation of **3c** after completion of the reaction.

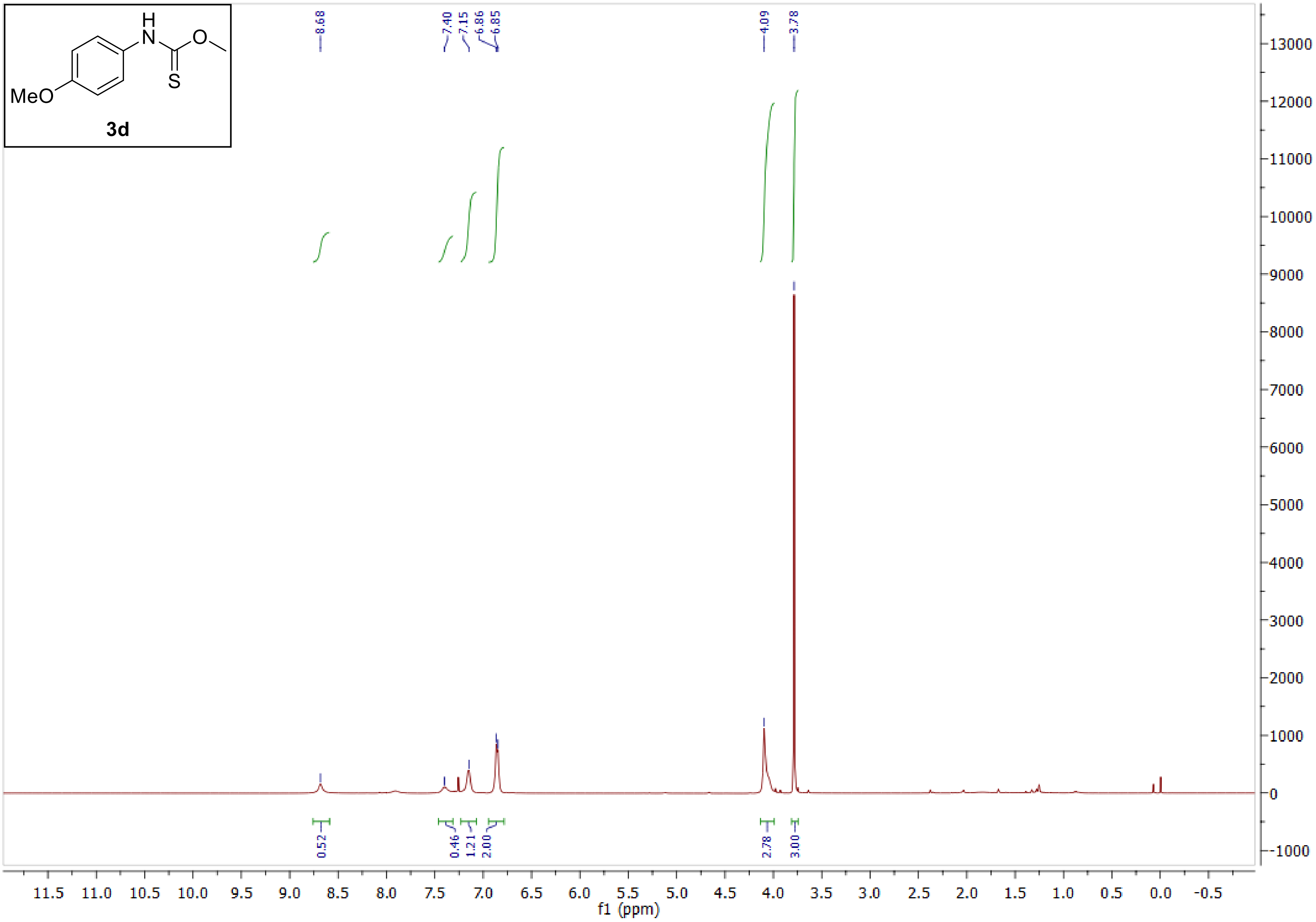
MeO
3d
8.68
7.40
7.15
6.86
6.85
4.09
3.78
0.52
0.46
1.21
2.00
2.78
3.00
f1 (ppm)

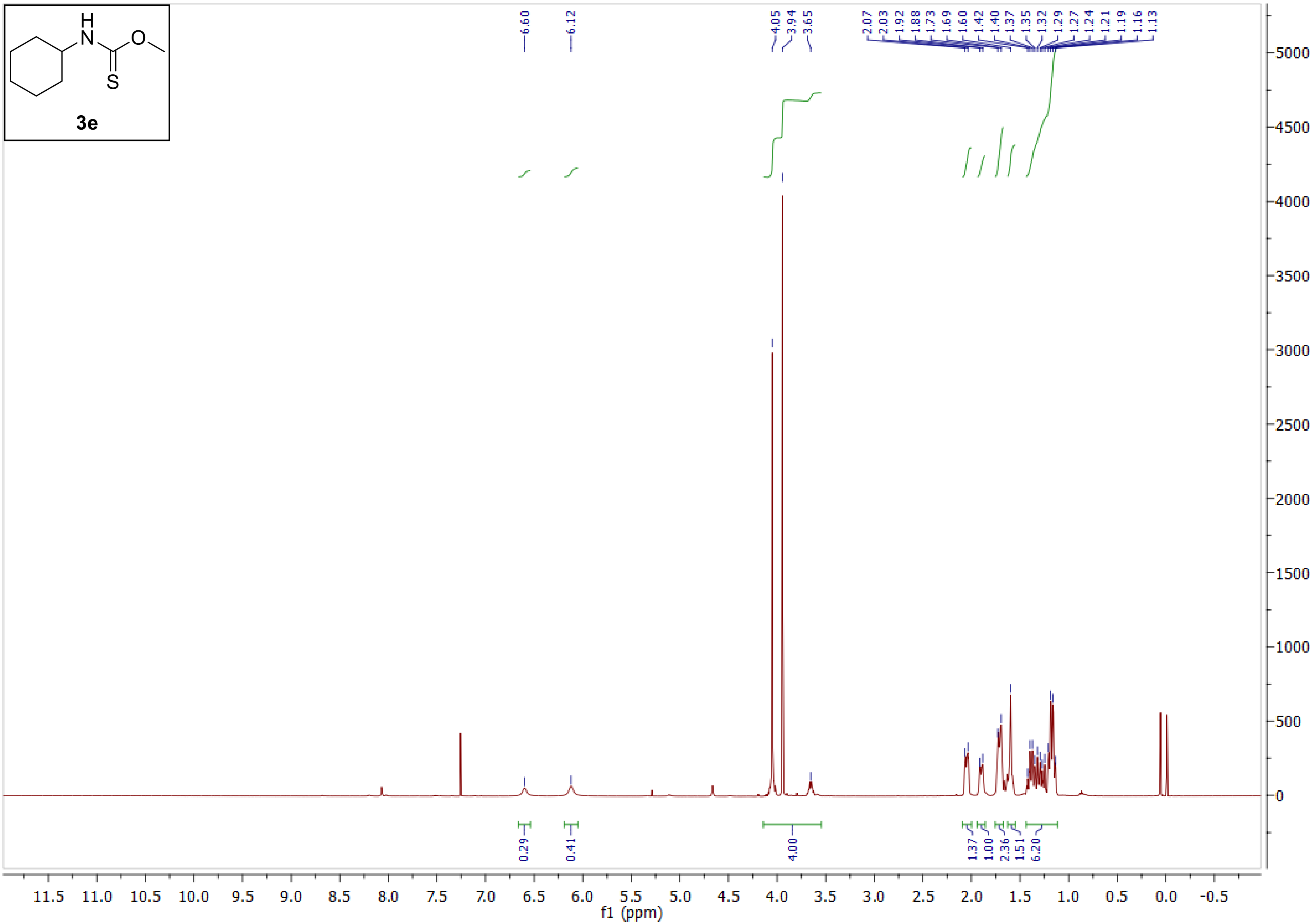

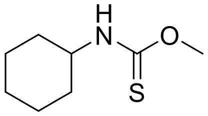

3e

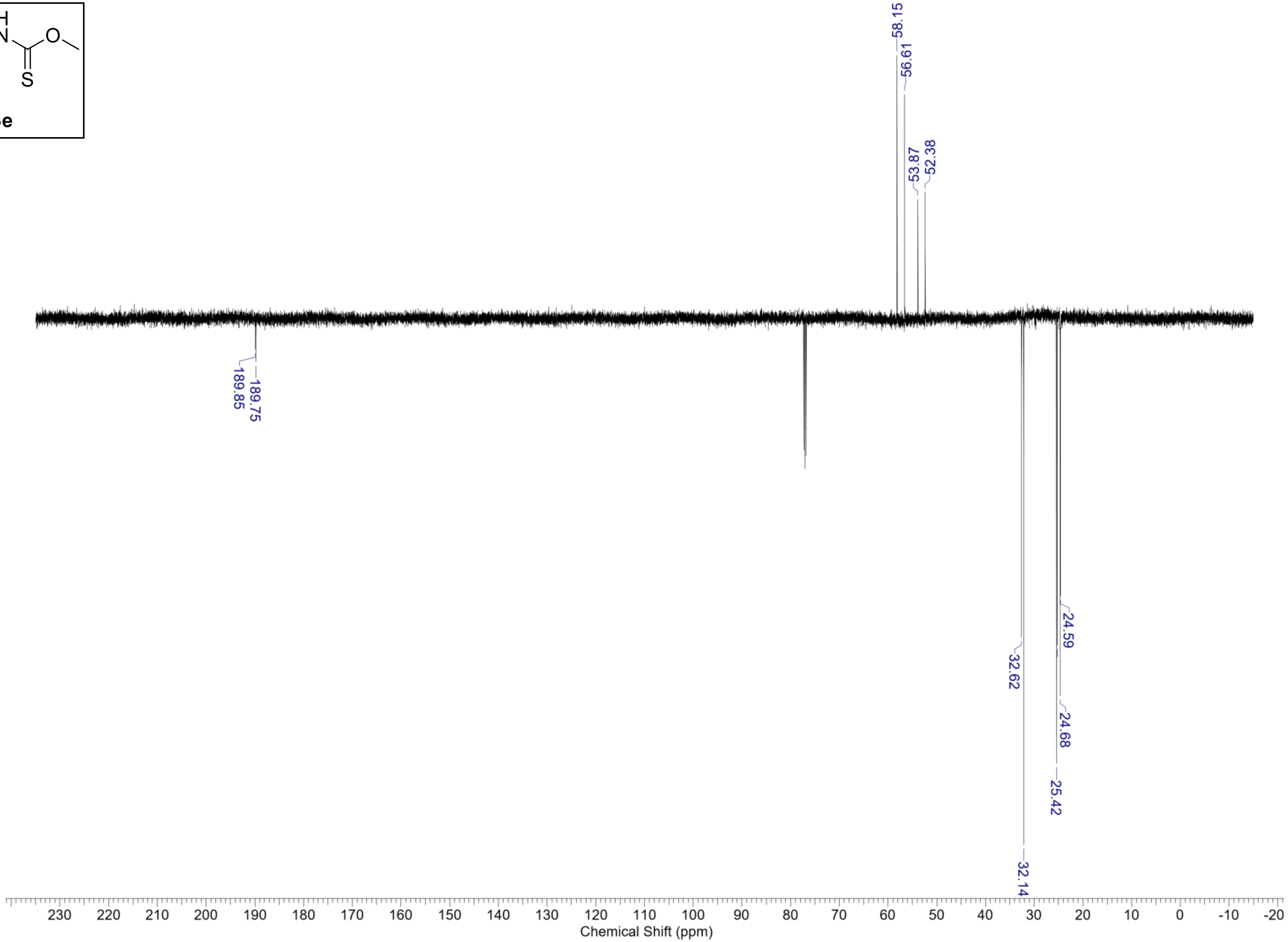

58.15
56.61
53.87
52.38
189.75
189.85
24.59
32.62
24.68
25.42
32.14
230 220 210 200 190 180 170 160 150 140 130 120 110 100 90 80 70 60 50 40 30 20 10 0 -10 -20
Chemical Shift (ppm)

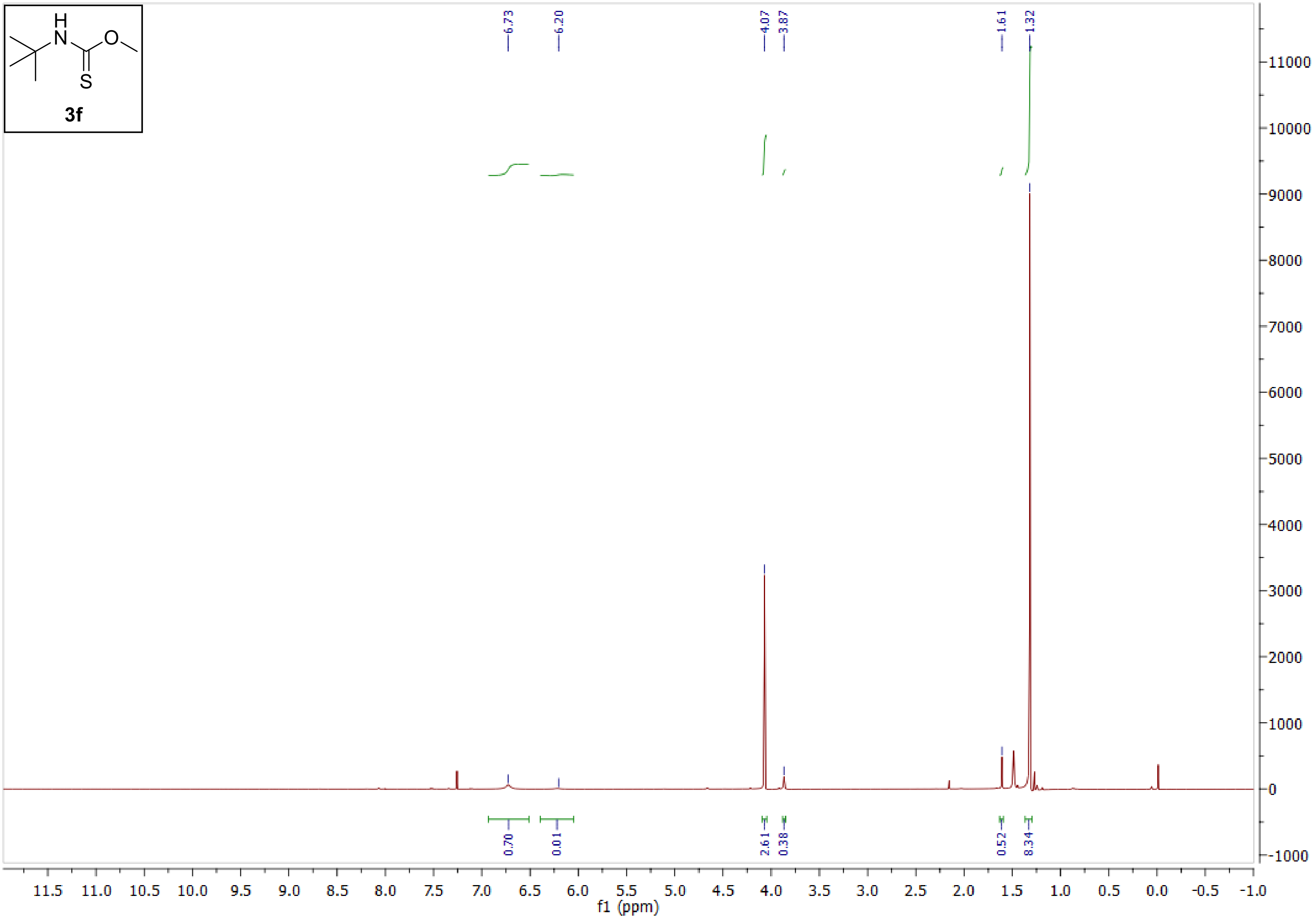
H
N
O
S
3f
6.73
6.20
4.07
3.87
1.61
1.32
0.70
0.01
2.61
0.38
0.52
8.34
11.5 11.0 10.5 10.0 9.5 9.0 8.5 8.0 7.5 7.0 6.5 6.0 5.5 5.0 4.5 4.0 3.5 3.0 2.5 2.0 1.5 1.0 0.5 0.0 -0.5 -1.0
f1 (ppm)
11000 10000 9000 8000 7000 6000 5000 4000 3000 2000 1000 0 -1000

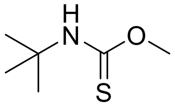
H
N
O
S

3f

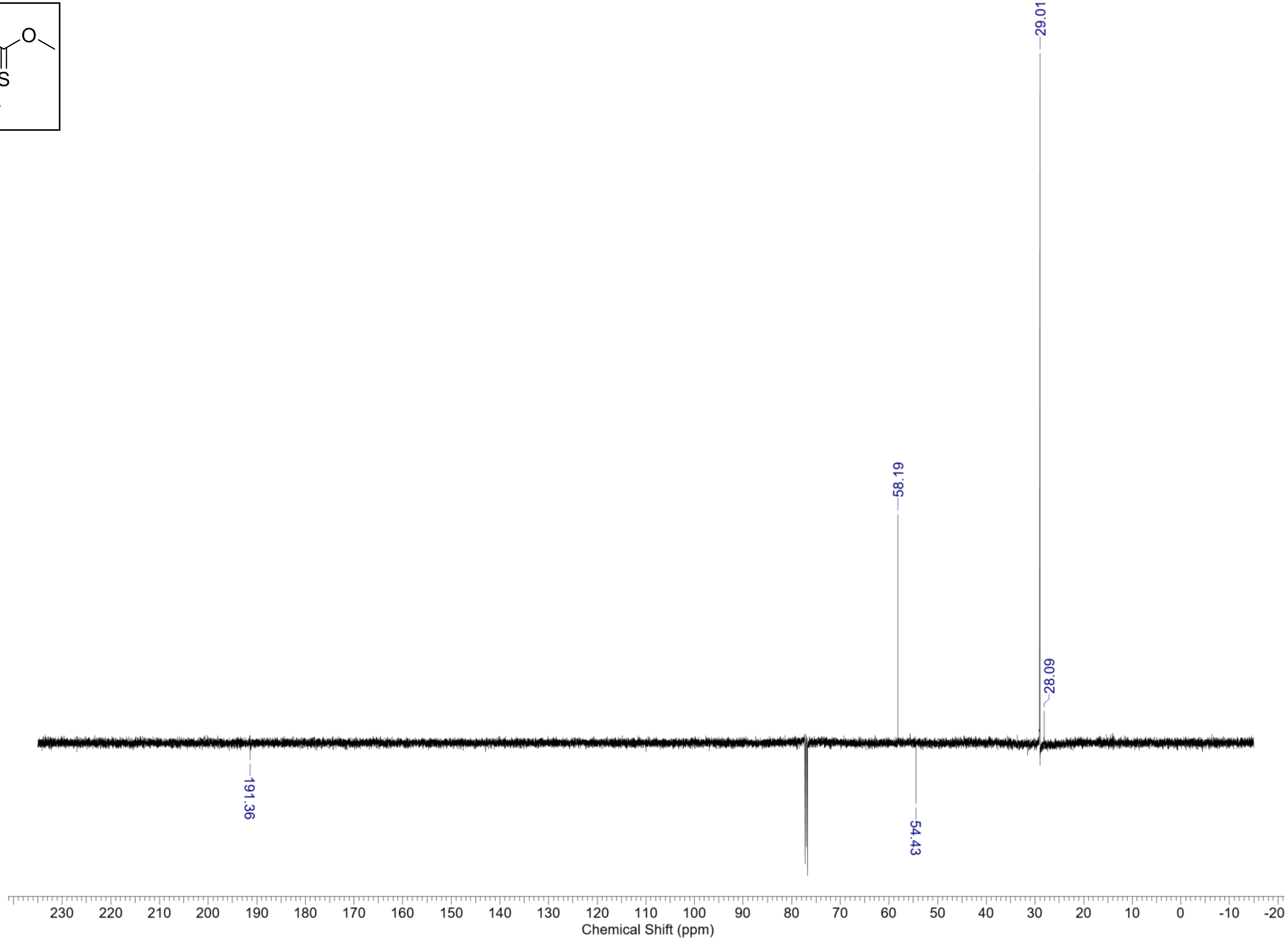
29.01
58.19
28.09
191.36
54.43
230 220 210 200 190 180 170 160 150 140 130 120 110 100 90 80 70 60 50 40 30 20 10 0 -10 -20
Chemical Shift (ppm)

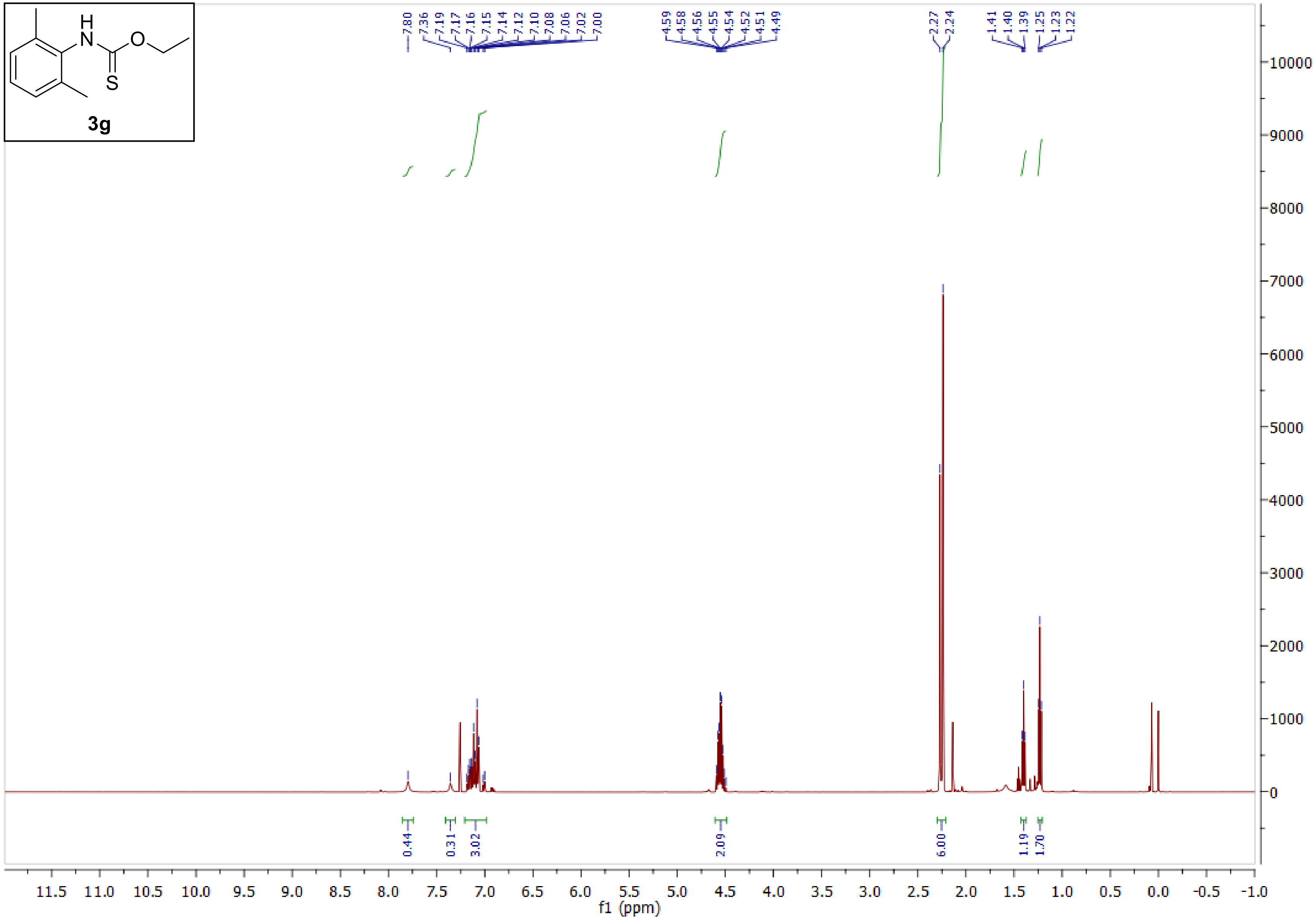

3g
7.80
7.36
7.19
7.17
7.16
7.15
7.14
7.12
7.10
7.08
7.06
7.02
7.00
4.59
4.58
4.56
4.55
4.54
4.52
4.51
4.49
2.27
2.24
1.41
1.40
1.39
1.25
1.23
1.22
0.44
0.31
3.02
2.09
6.00
1.19
1.70
f1 (ppm)

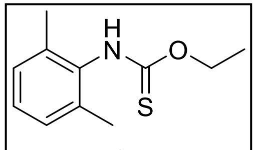

3g

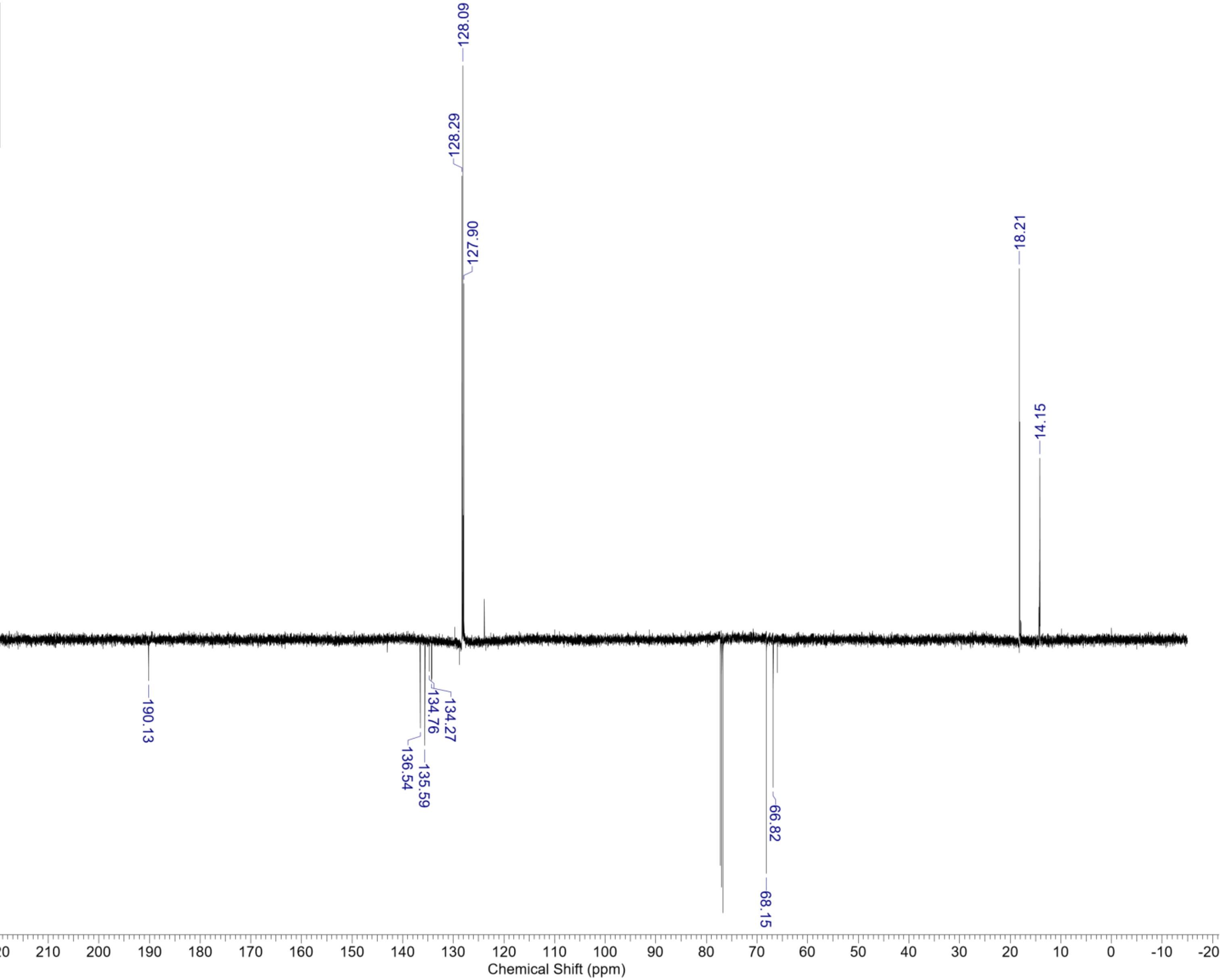

128.09
128.29
127.90
18.21
14.15
190.13
134.76
134.27
136.54
135.59
66.82
68.15
230
220
210
200
190
180
170
160
150
140
130
120
110
100
90
80
70
60
50
40
30
20
10
0
-10
-20
Chemical Shift (ppm)

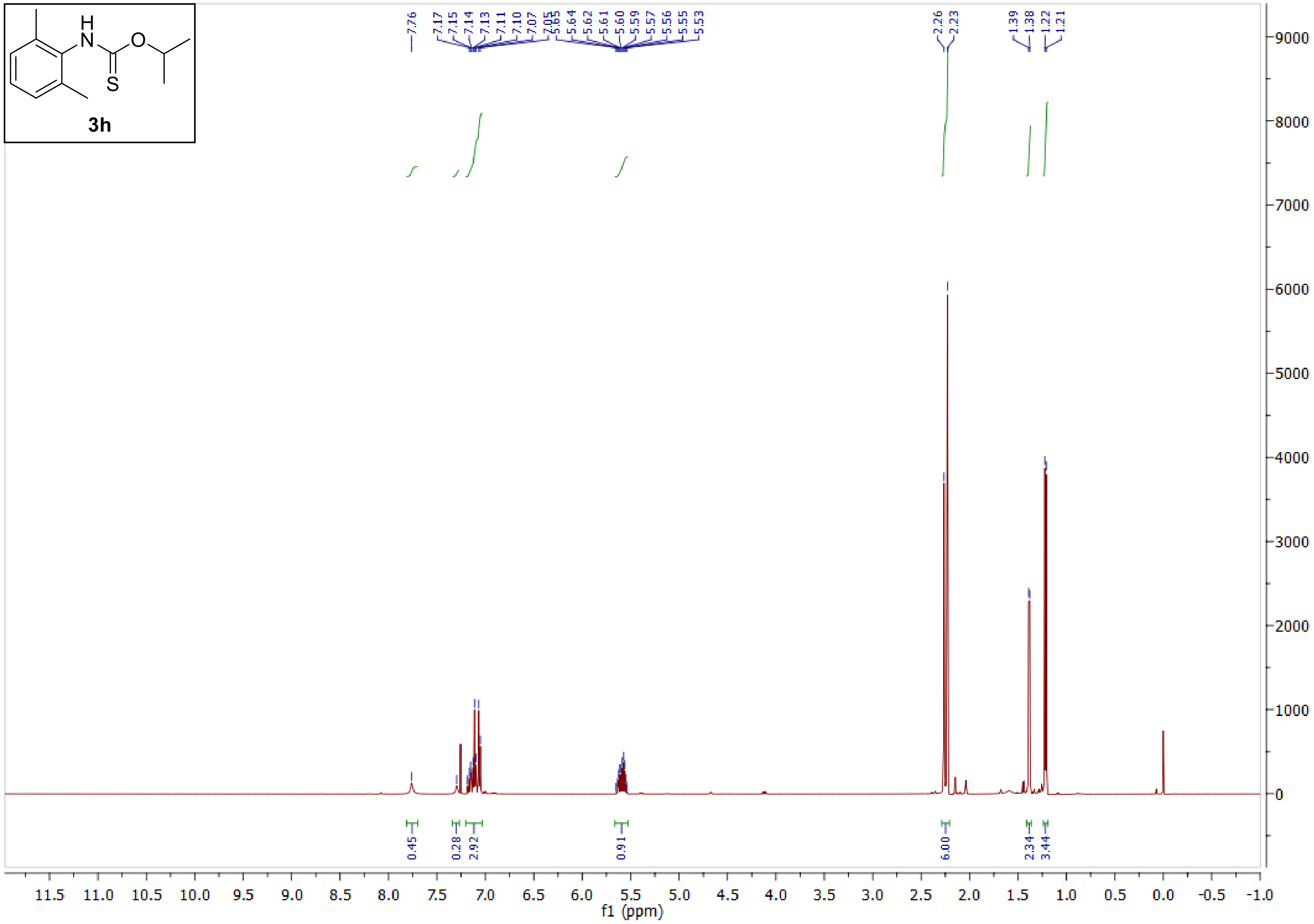

3h
f1 (ppm)

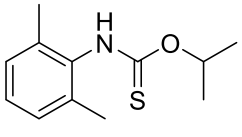

H
N
O
S

3h

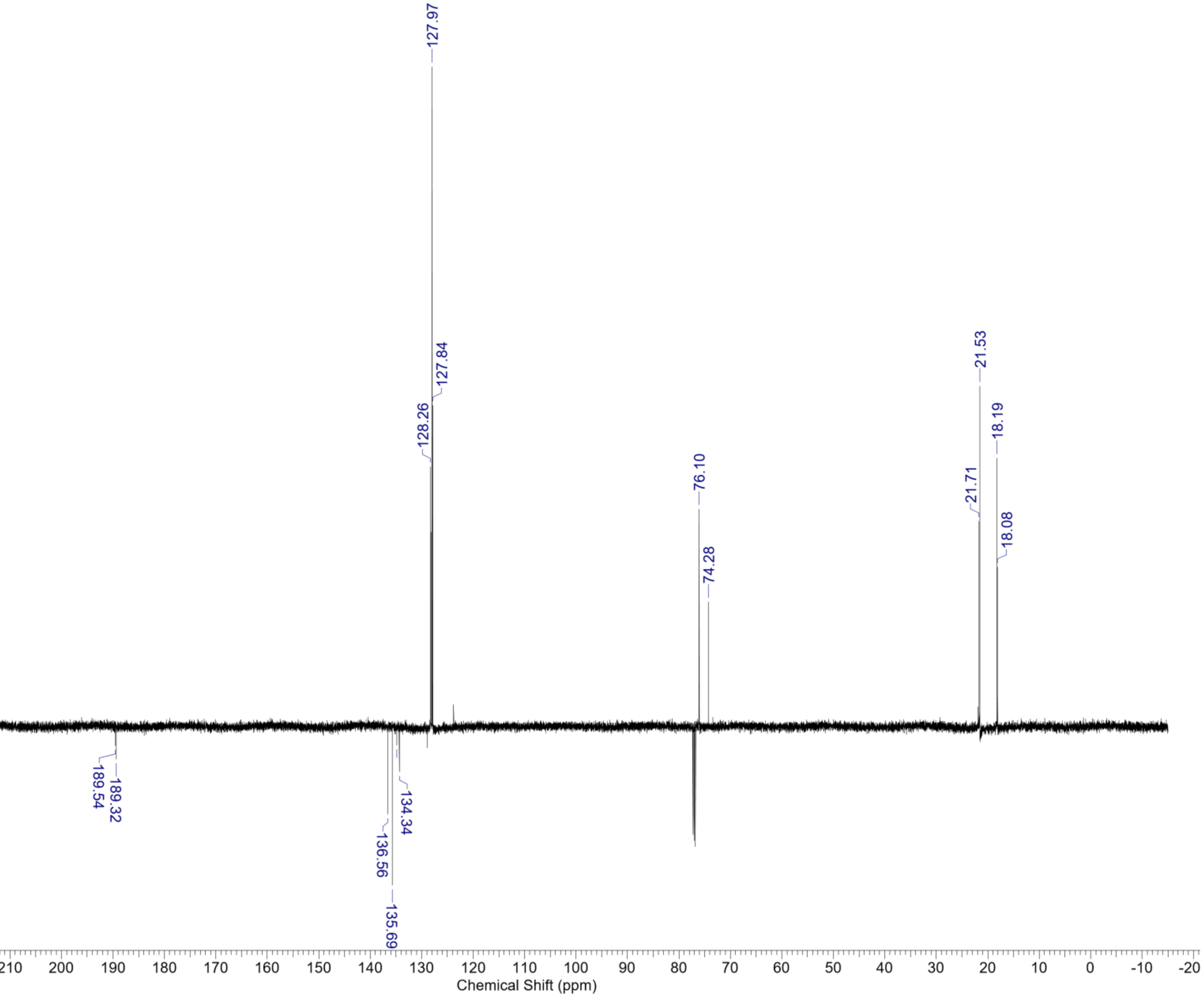

127.97
127.84
128.26
76.10
74.28
21.53
21.71
18.19
18.08
189.54
189.32
134.34
136.56
135.69
230 220 210 200 190 180 170 160 150 140 130 120 110 100 90 80 70 60 50 40 30 20 10 0 -10 -20
Chemical Shift (ppm)

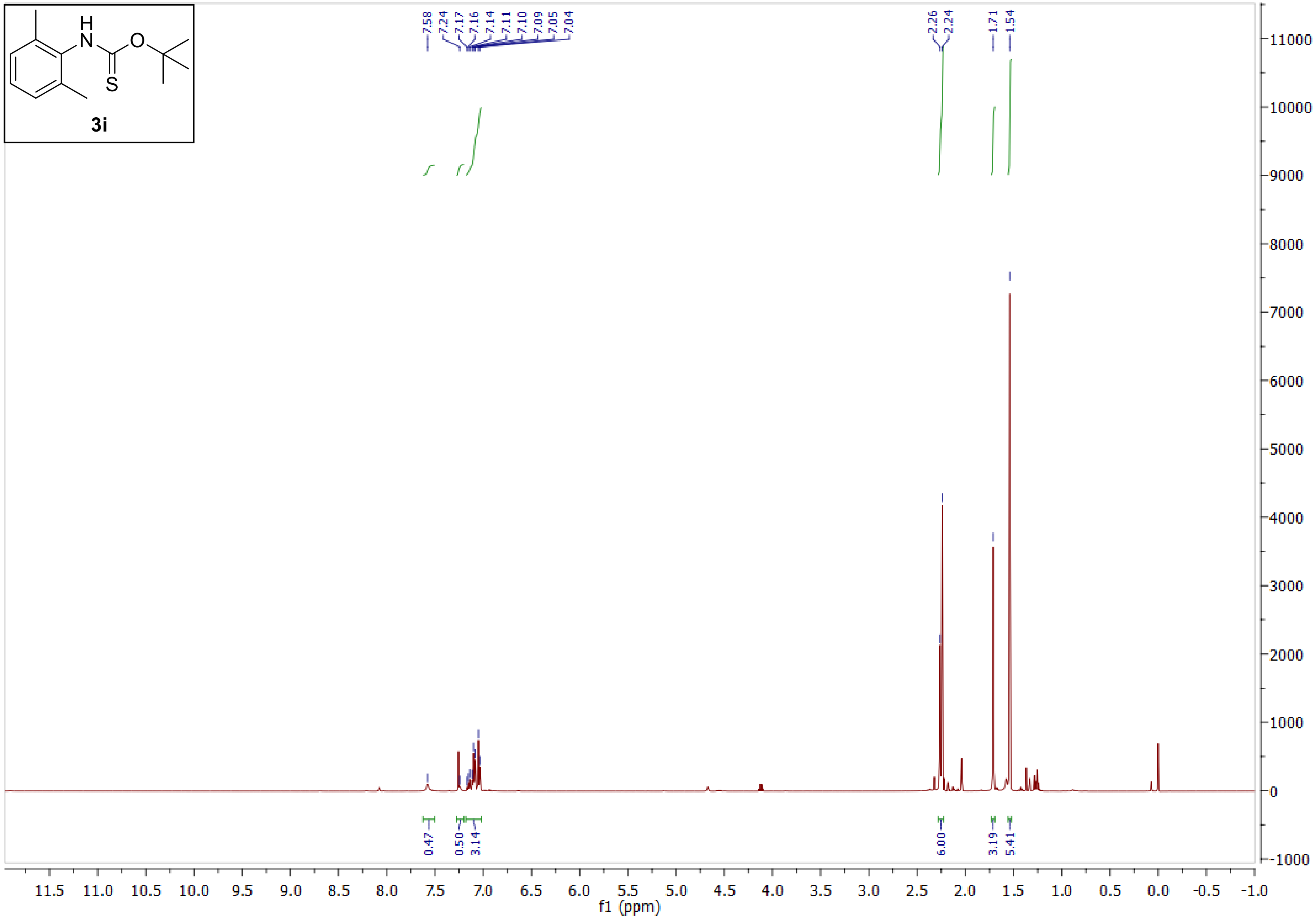

H
N
O
S
3i
7.58
7.24
7.17
7.16
7.14
7.11
7.10
7.09
7.05
7.04
2.26
2.24
1.71
1.54
0.47
0.50
3.14
6.00
3.19
5.41
f1 (ppm)

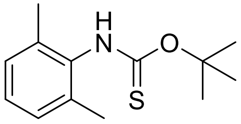
H
N
O
S

3i

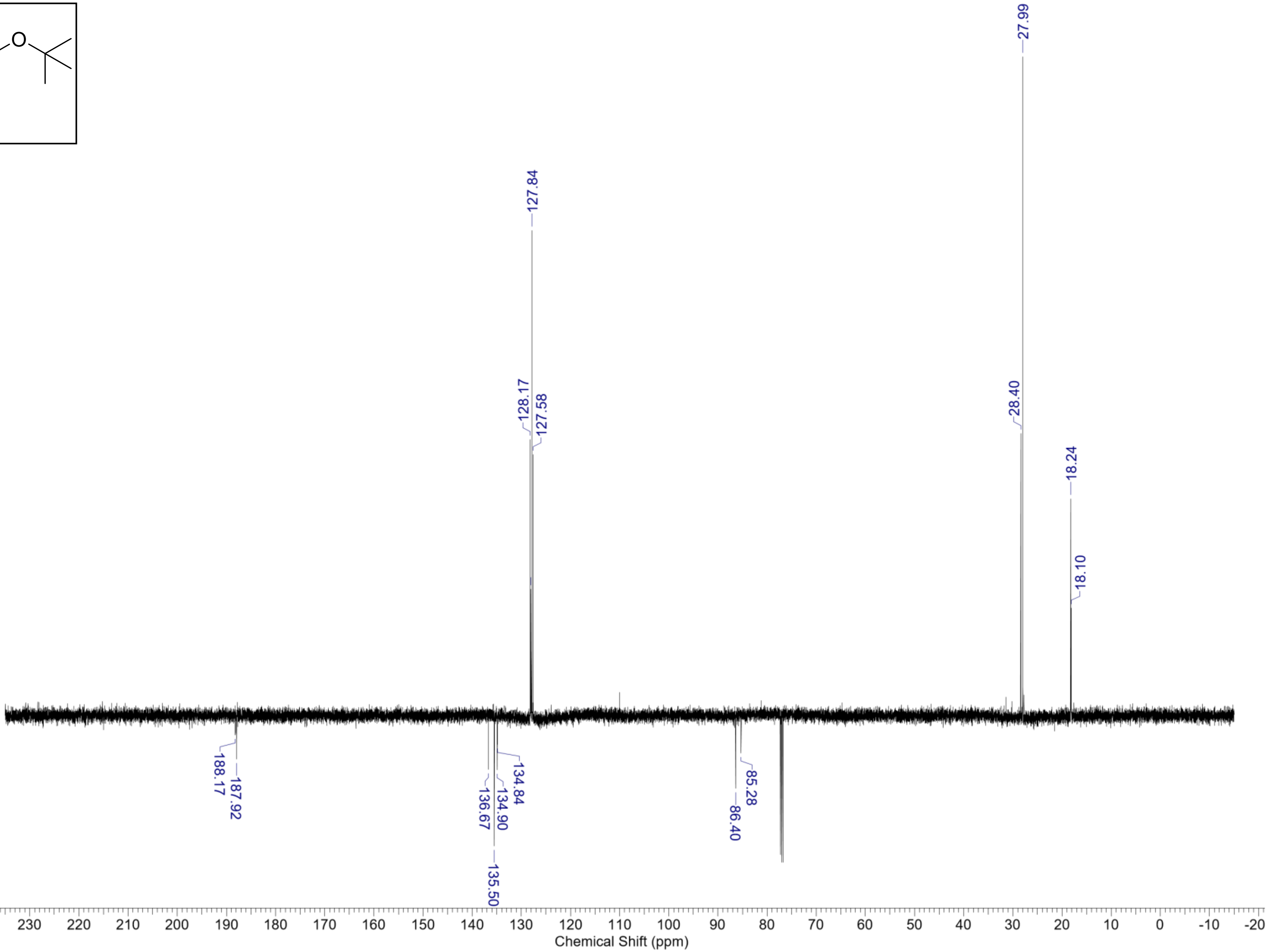
27.99
127.84
128.17
127.58
28.40
18.24
18.10
188.17
187.92
136.67
134.90
134.84
135.50
85.28
86.40
230 220 210 200 190 180 170 160 150 140 130 120 110 100 90 80 70 60 50 40 30 20 10 0 -10 -20
Chemical Shift (ppm)

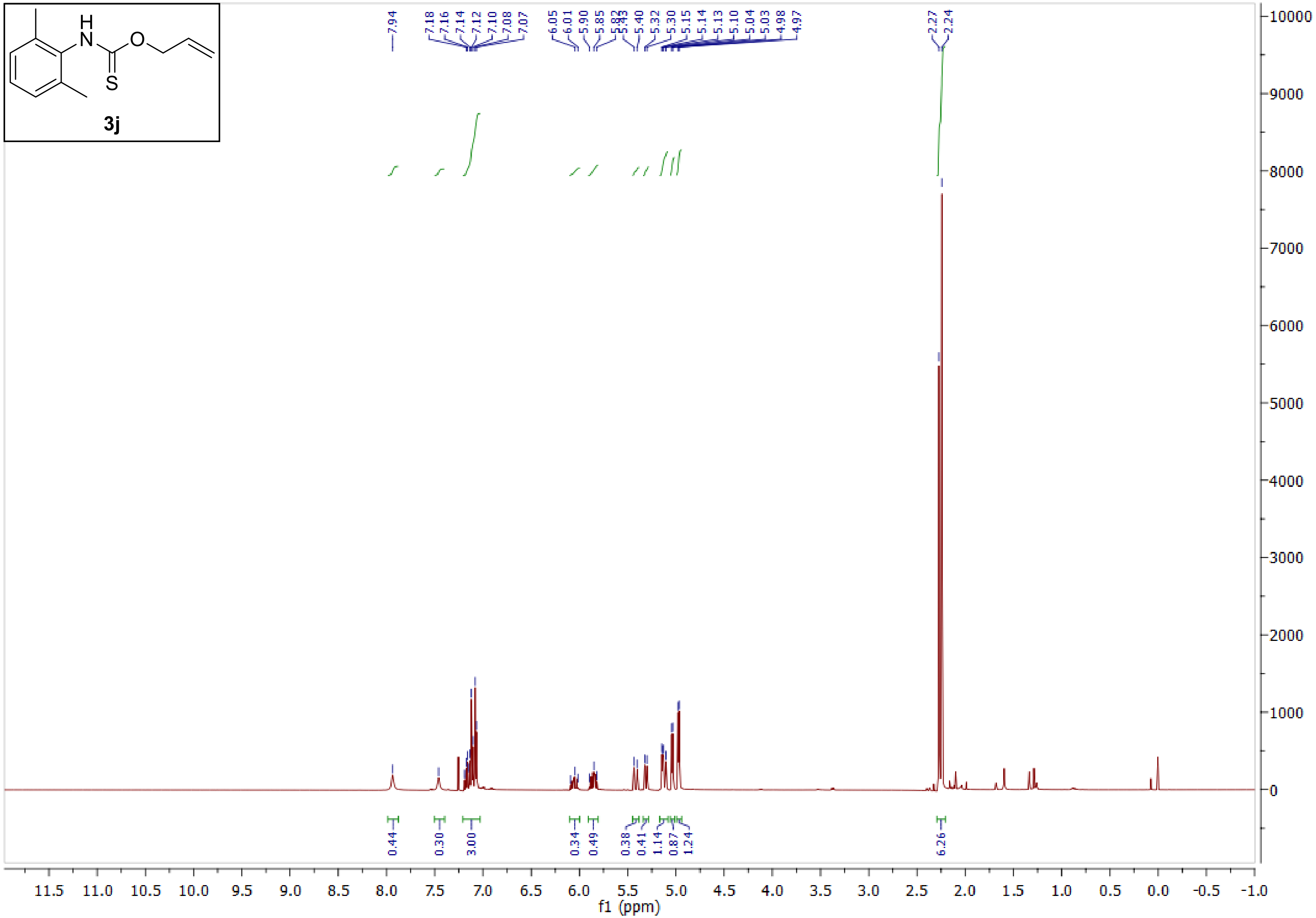
3j
7.94
7.18
7.16
7.14
7.12
7.10
7.08
7.07
6.05
6.01
5.90
5.85
5.82
5.43
5.40
5.32
5.30
5.15
5.14
5.13
5.10
5.04
5.03
4.98
4.97
2.27
2.24
0.44
0.30
3.00
0.34
0.49
0.38
0.41
1.14
0.87
1.24
6.26
f1 (ppm)

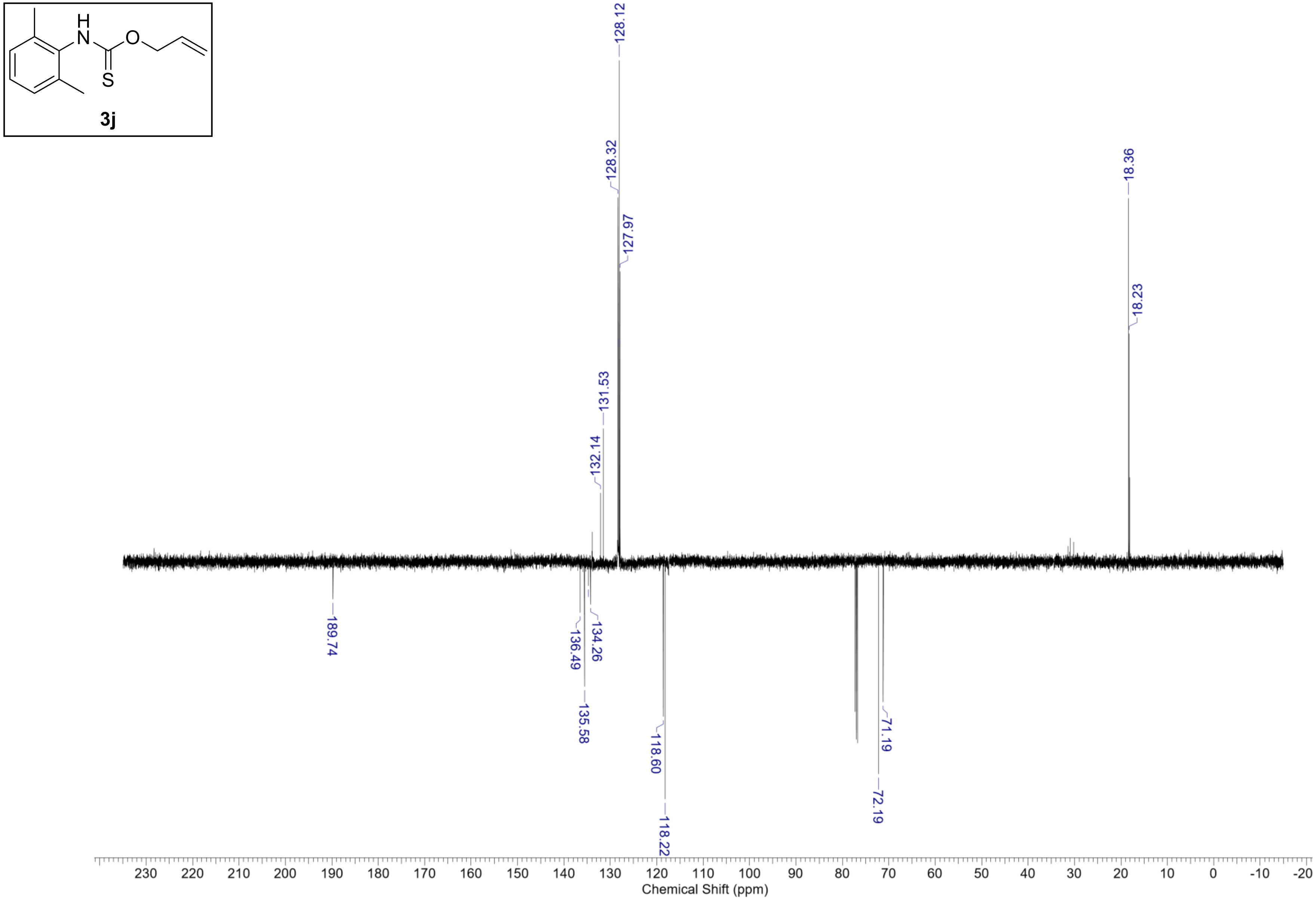
3j
128.12
128.32
127.97
18.36
18.23
131.53
132.14
189.74
136.49
134.26
135.58
118.60
118.22
71.19
72.19
230 220 210 200 190 180 170 160 150 140 130 120 110 100 90 80 70 60 50 40 30 20 10 0 -10 -20
Chemical Shift (ppm)

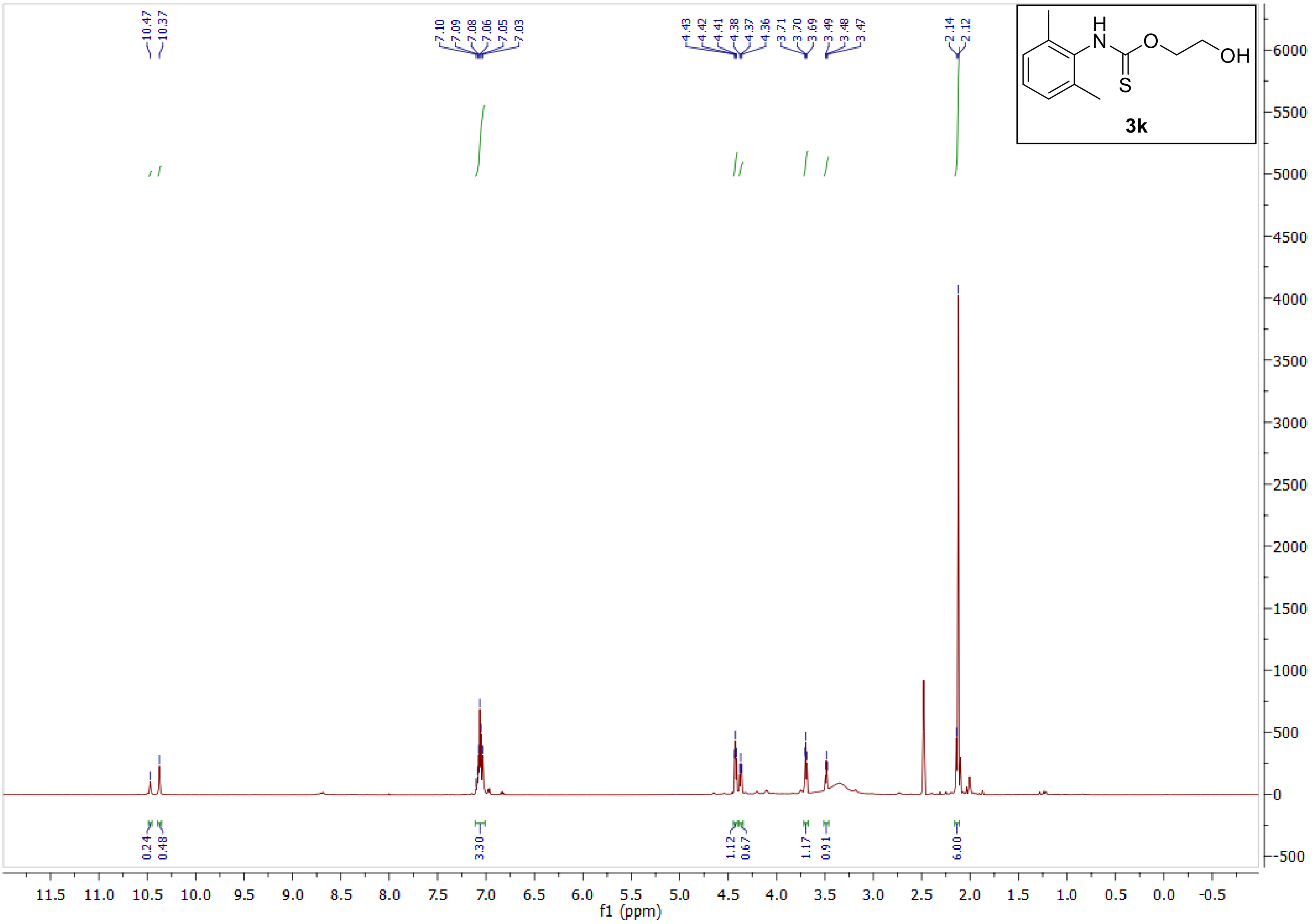
10.47
10.37
7.10
7.09
7.08
7.06
7.05
7.03
4.43
4.42
4.41
4.38
4.37
4.36
3.71
3.70
3.69
3.49
3.48
3.47
2.14
2.12
H
N
O
OH
S
3k
0.24
0.48
3.30
1.12
0.67
1.17
0.91
6.00
f1 (ppm)

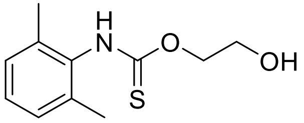
H
N
O
OH
S

3k

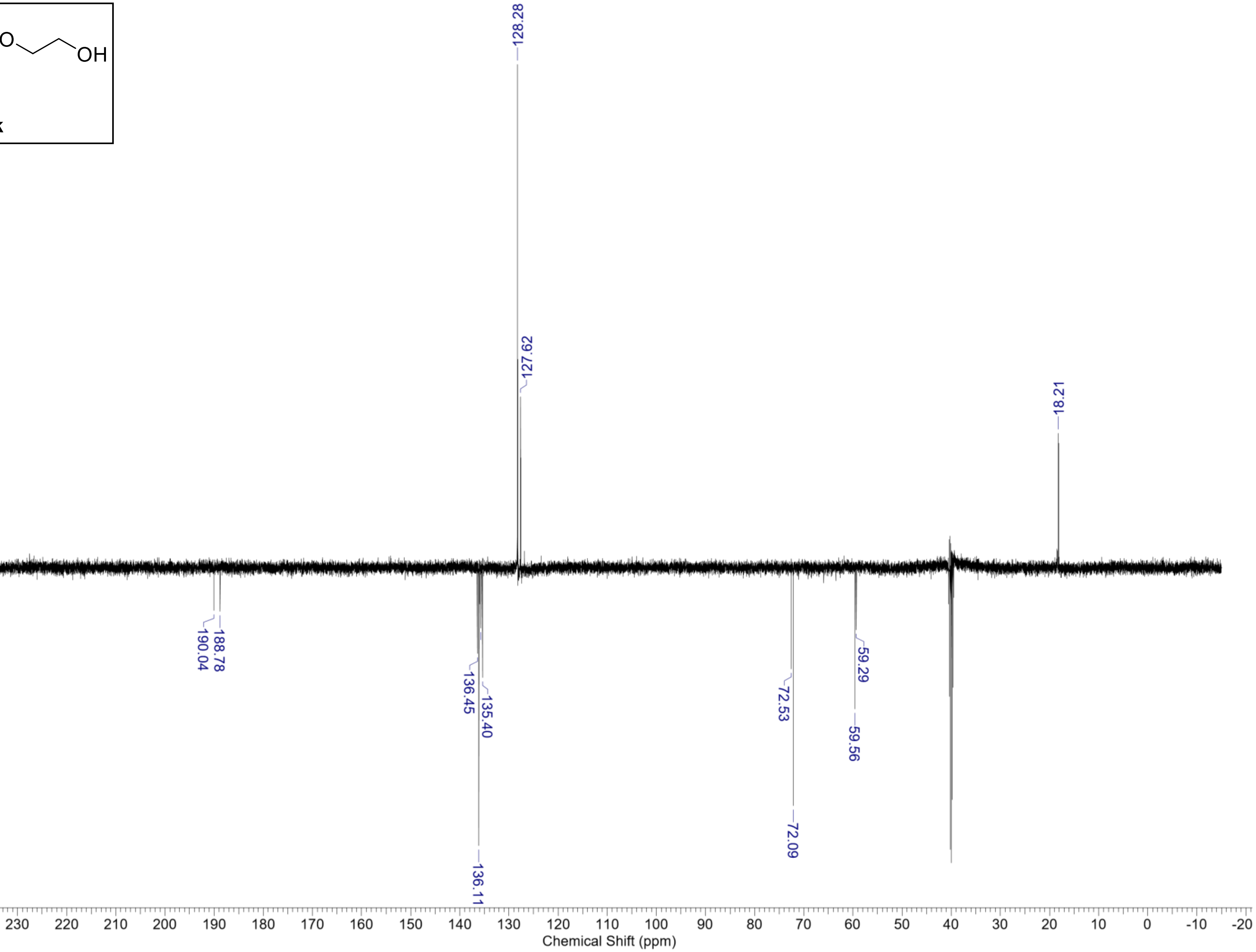
128.28
127.62
18.21
190.04
188.78
136.45
135.40
136.11
72.53
72.09
59.29
59.56
230 220 210 200 190 180 170 160 150 140 130 120 110 100 90 80 70 60 50 40 30 20 10 0 -10 -20
Chemical Shift (ppm)

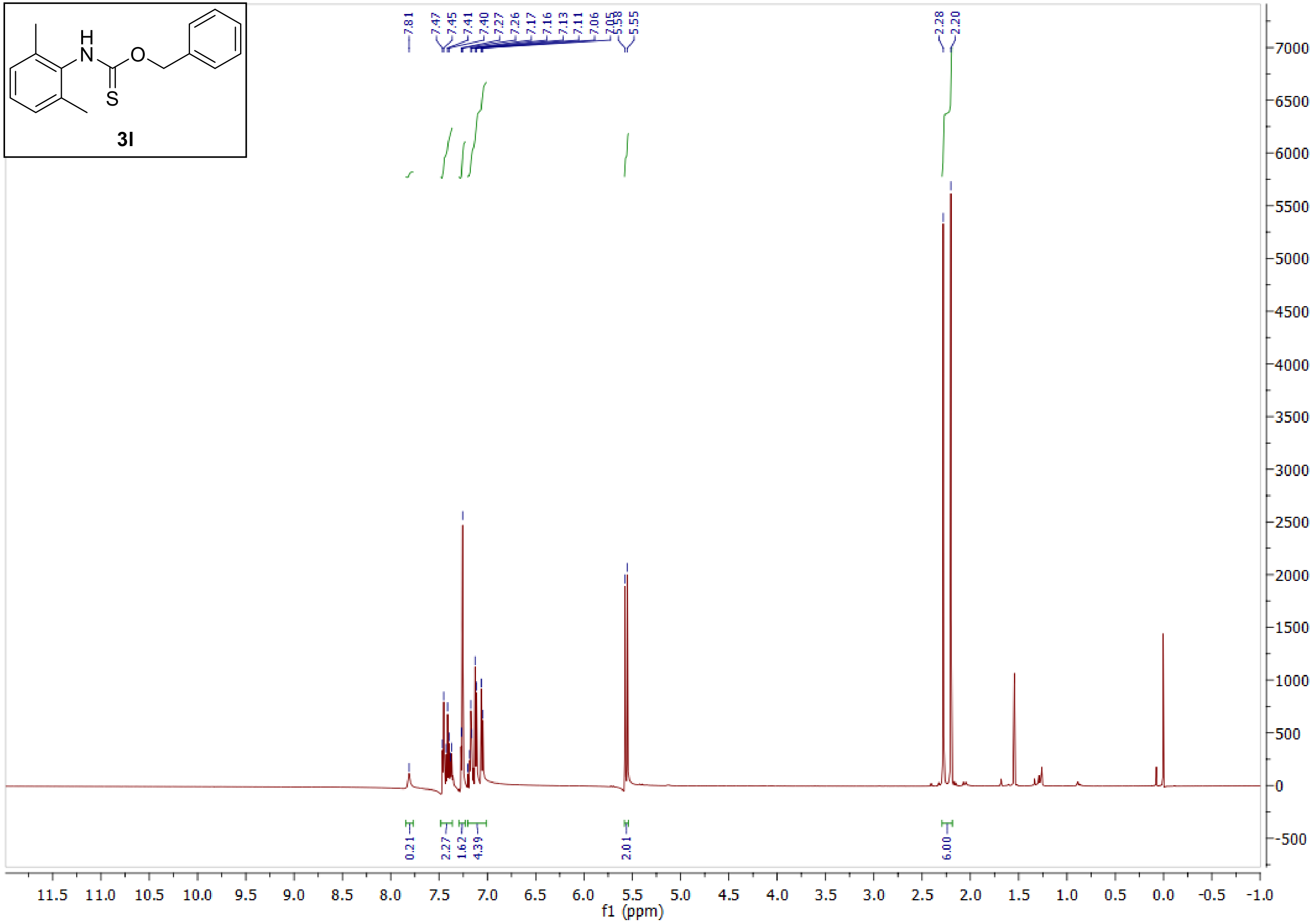
3l
7.81
7.47
7.45
7.41
7.40
7.27
7.26
7.17
7.16
7.13
7.11
7.06
7.05
5.58
5.55
2.28
2.20
0.21
2.27
1.62
4.39
2.01
6.00
f1 (ppm)

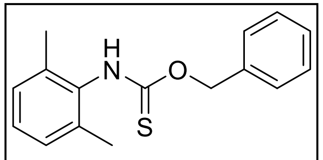
H
N
O
S

3l

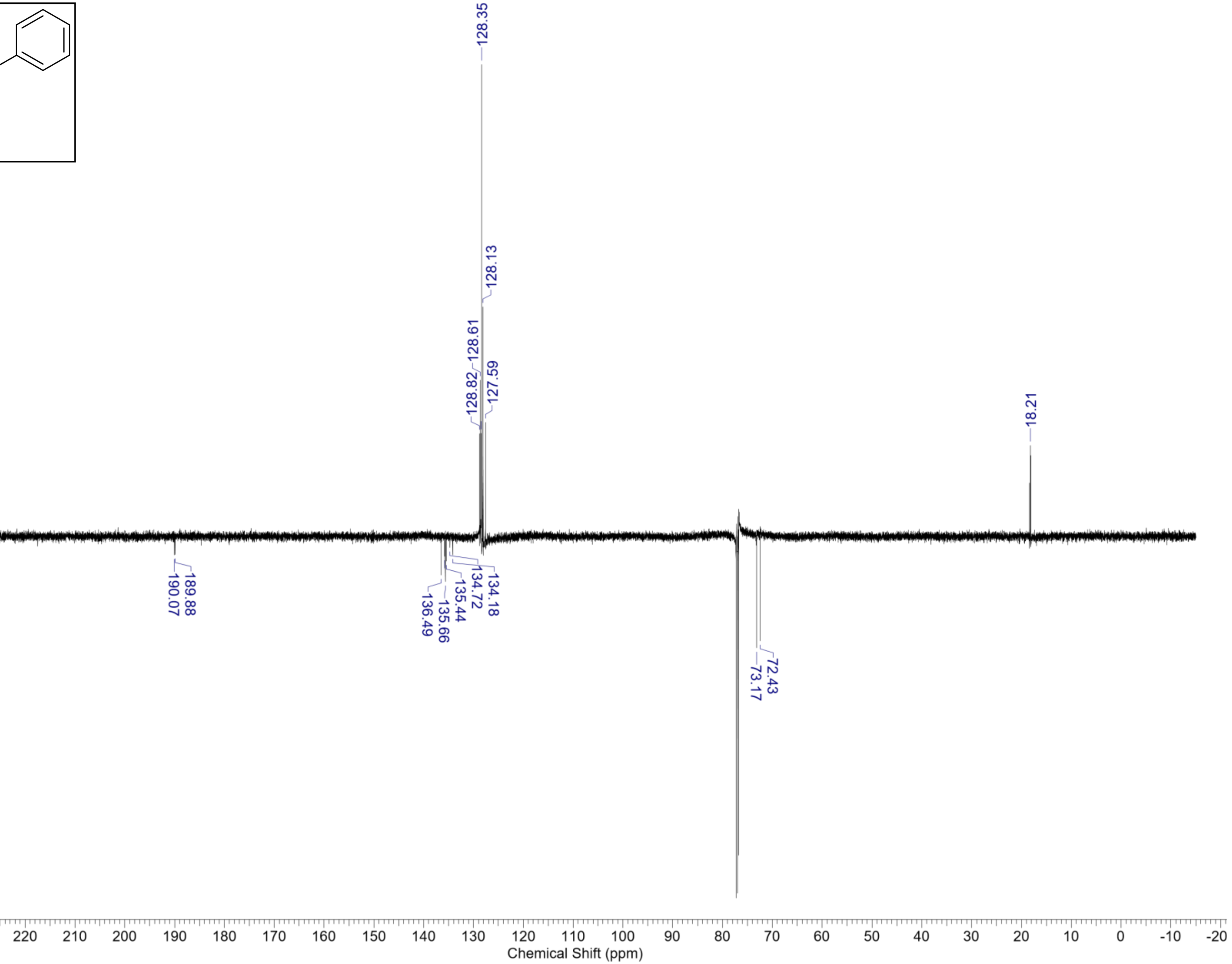
128.35
128.13
128.82
128.61
127.59
18.21
189.88
190.07
134.18
134.72
135.44
135.66
136.49
72.43
73.17
230 220 210 200 190 180 170 160 150 140 130 120 110 100 90 80 70 60 50 40 30 20 10 0 -10 -20
Chemical Shift (ppm)

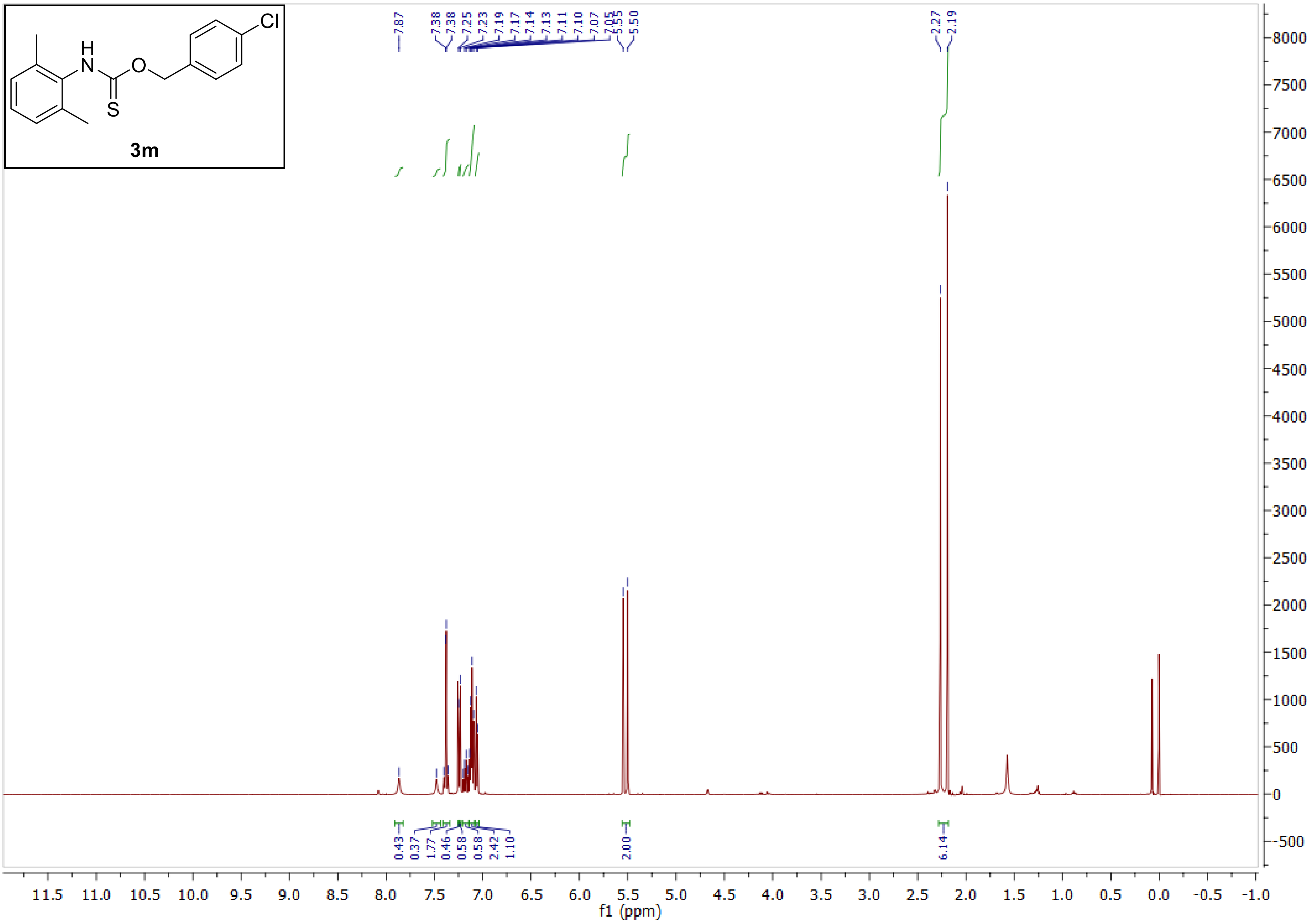

3m
7.87
7.38
7.38
7.25
7.23
7.19
7.17
7.14
7.13
7.11
7.10
7.07
7.05
5.55
5.50
2.27
2.19
0.43
0.37
1.77
0.46
0.58
0.58
2.42
1.10
2.00
6.14
f1 (ppm)

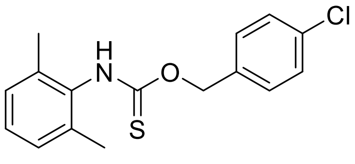
Cl
H
N
O
S

3m

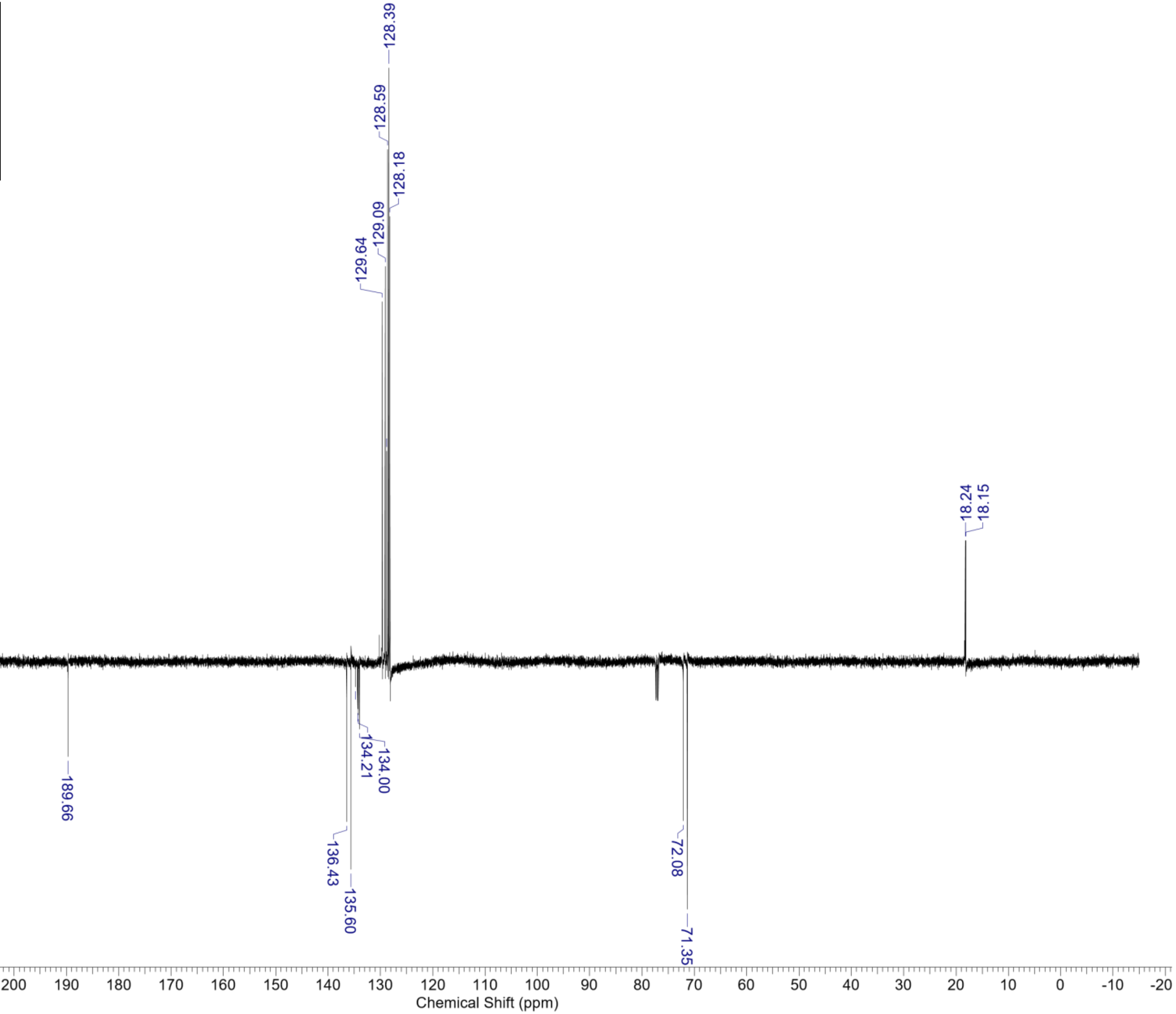
128.39
128.59
128.18
129.09
129.64
18.24
18.15
189.66
134.21
134.00
136.43
135.60
72.08
71.35
230 220 210 200 190 180 170 160 150 140 130 120 110 100 90 80 70 60 50 40 30 20 10 0 -10 -20
Chemical Shift (ppm)

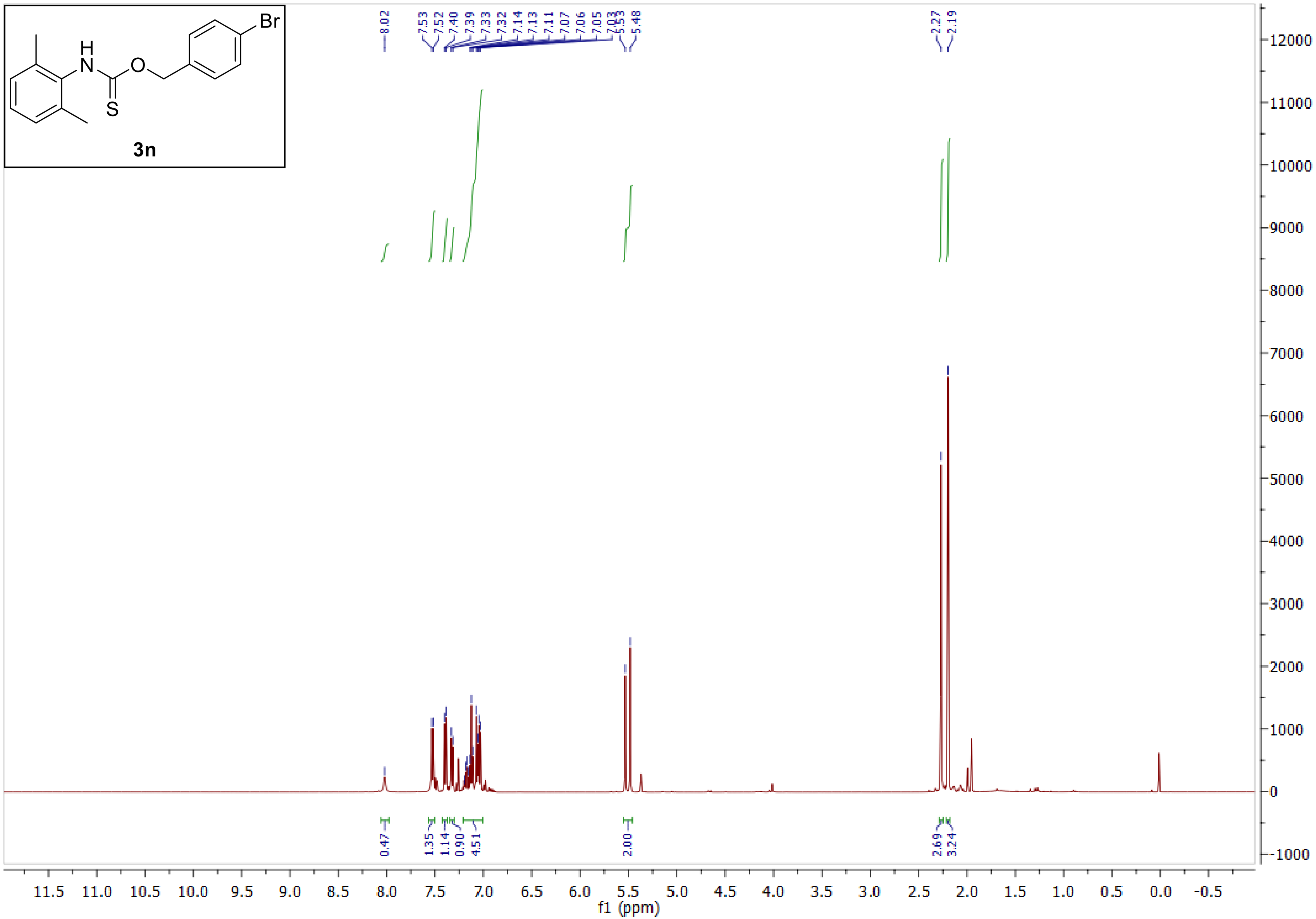

Br
H
N
O
S
3n
8.02
7.53
7.52
7.40
7.39
7.33
7.32
7.14
7.13
7.11
7.07
7.06
7.05
7.03
5.53
5.48
2.27
2.19
0.47
1.35
1.14
0.90
4.51
2.00
2.69
3.24
f1 (ppm)

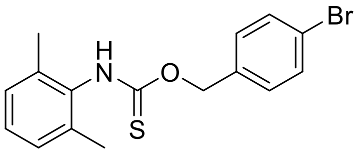
Br
H
N
O
S

3n

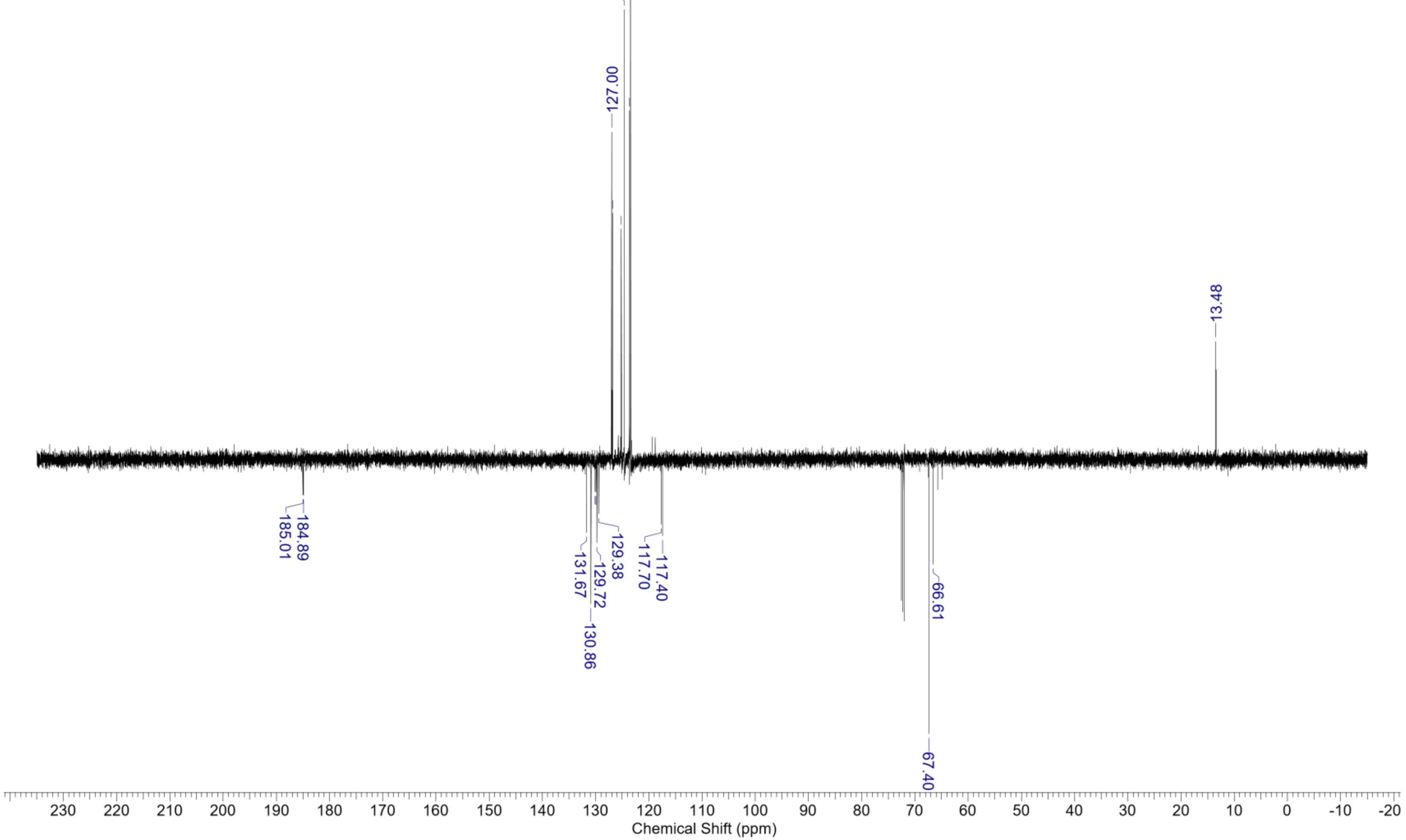
123.43
124.58
127.00
13.48
184.89
185.01
131.67
129.72
129.38
130.86
117.70
117.40
66.61
67.40
230 220 210 200 190 180 170 160 150 140 130 120 110 100 90 80 70 60 50 40 30 20 10 0 -10 -20
Chemical Shift (ppm)

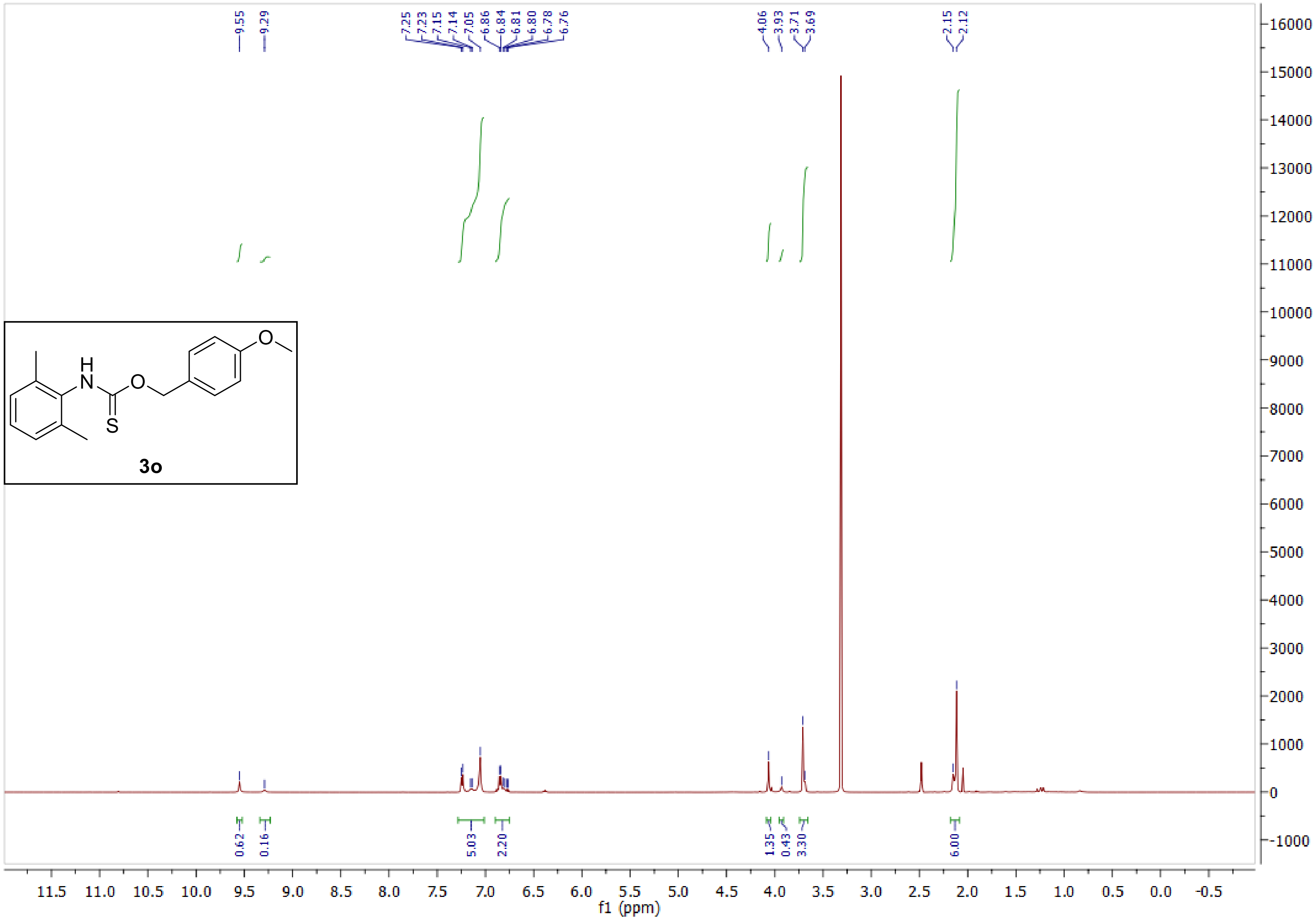

9.55
9.29
7.25
7.23
7.15
7.14
7.05
6.86
6.84
6.81
6.80
6.78
6.76
4.06
3.93
3.71
3.69
2.15
2.12
3o
0.62
0.16
5.03
2.20
1.35
0.43
3.30
6.00
f1 (ppm)

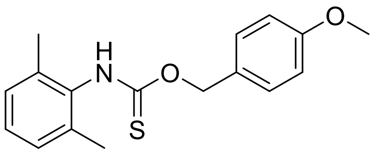

3o

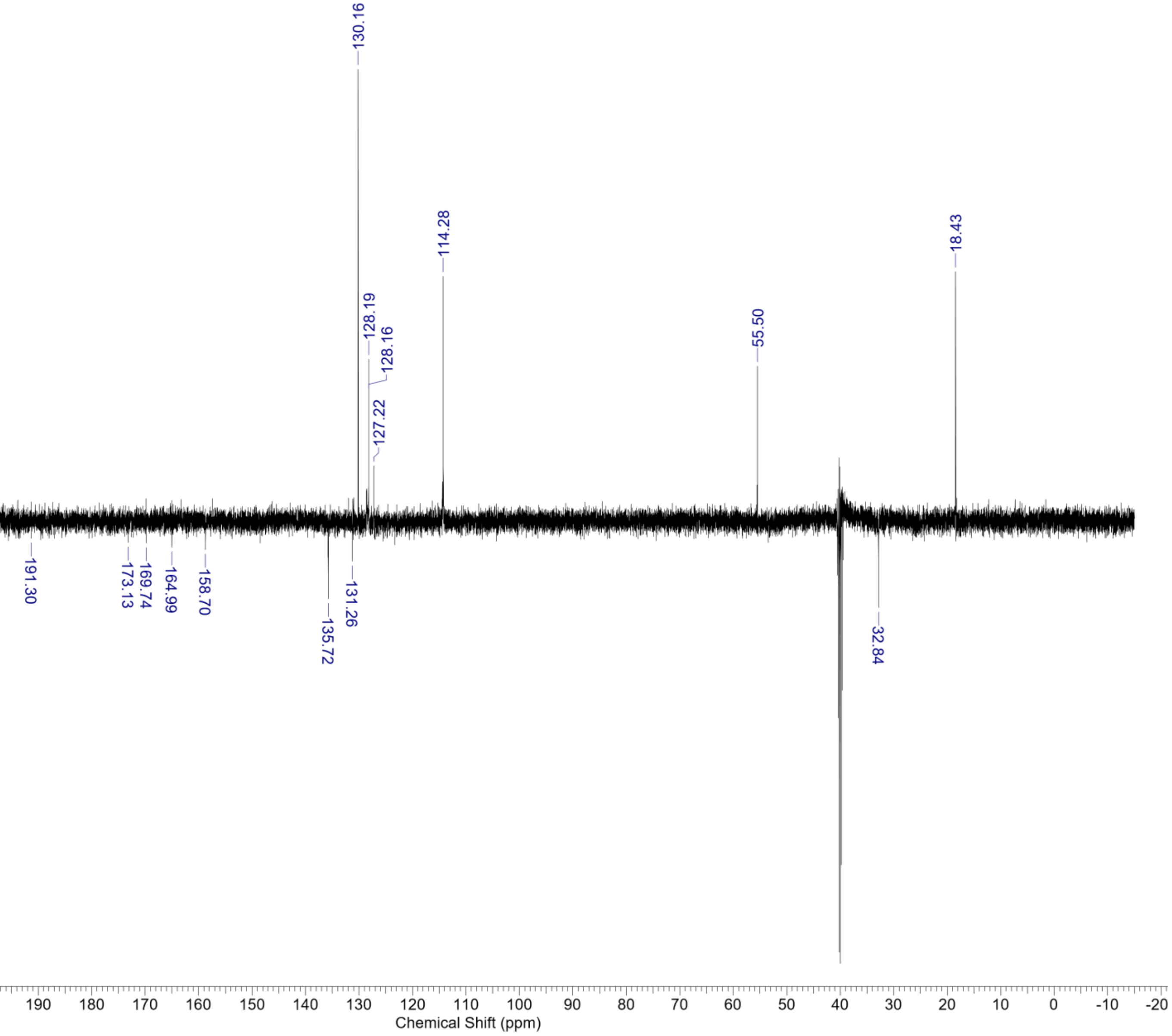
130.16
128.19
128.16
127.22
114.28
55.50
18.43
191.30
173.13
169.74
164.99
158.70
135.72
131.26
32.84
230 220 210 200 190 180 170 160 150 140 130 120 110 100 90 80 70 60 50 40 30 20 10 0 -10 -20
Chemical Shift (ppm)

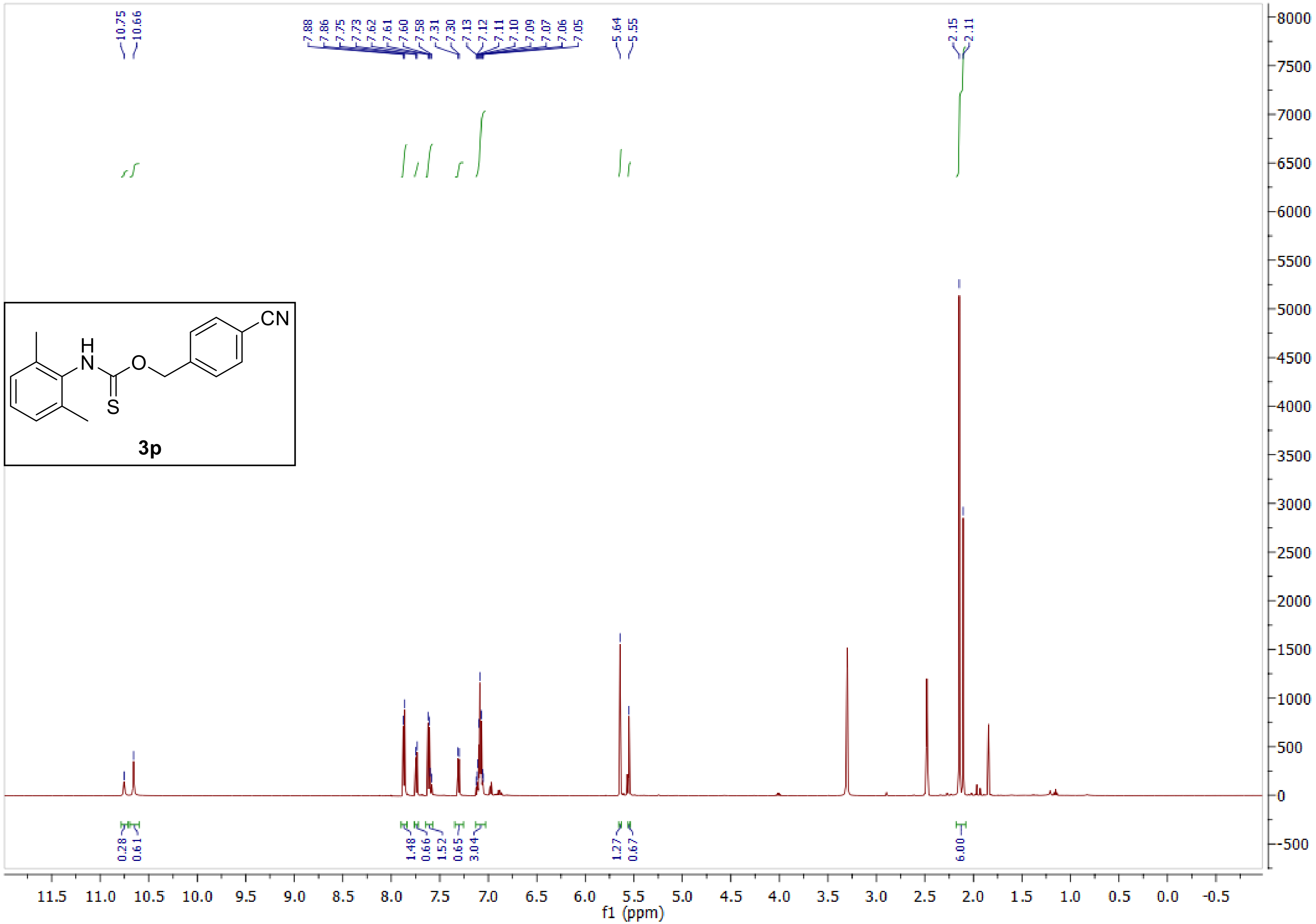

3p
f1 (ppm)

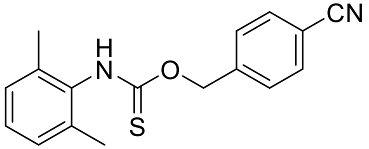
H
N
O
S
CN

3p

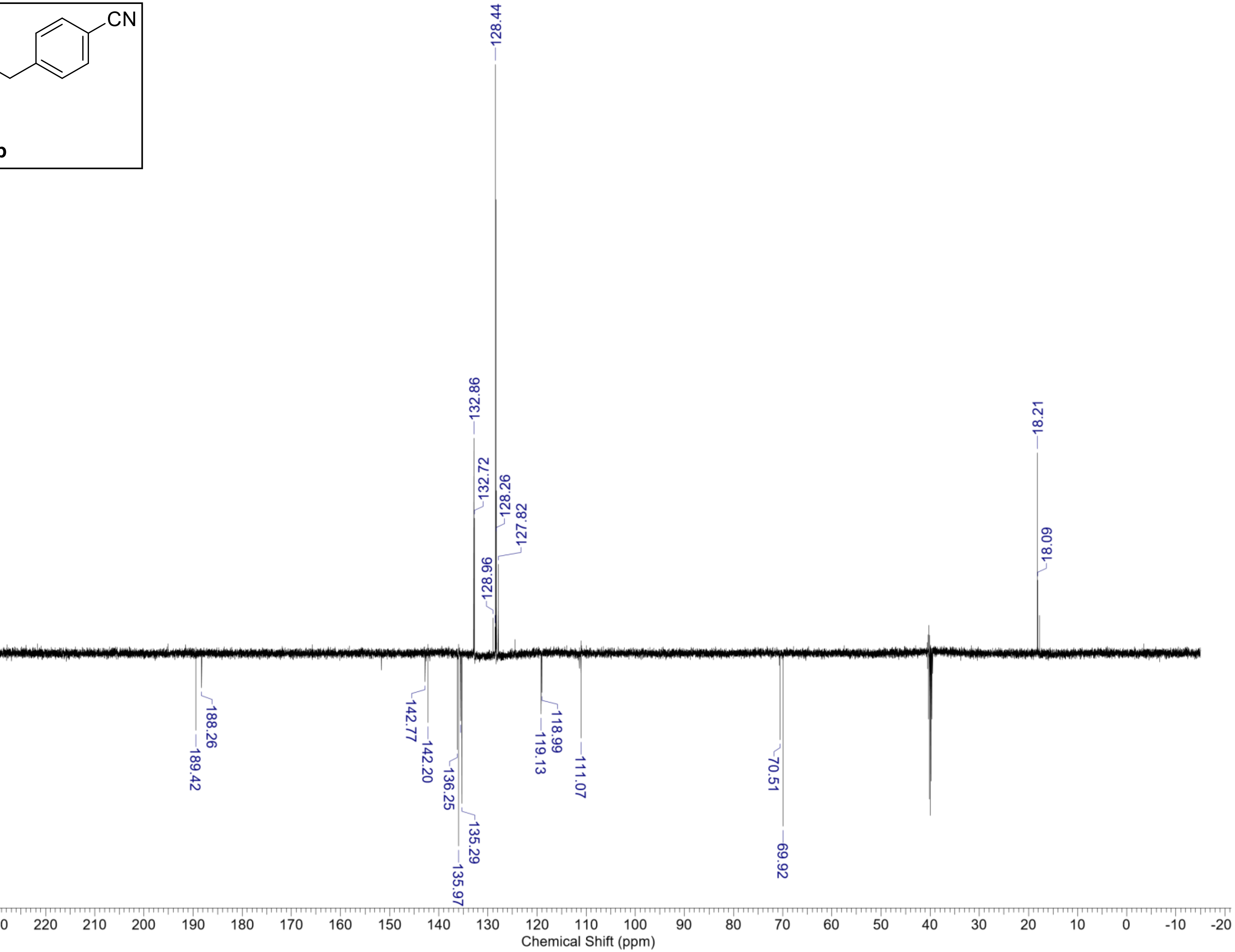
128.44
132.86
132.72
128.26
127.82
128.96
18.21
18.09
188.26
189.42
142.77
142.20
136.25
135.29
135.97
118.99
119.13
111.07
70.51
69.92
230 220 210 200 190 180 170 160 150 140 130 120 110 100 90 80 70 60 50 40 30 20 10 0 -10 -20
Chemical Shift (ppm)

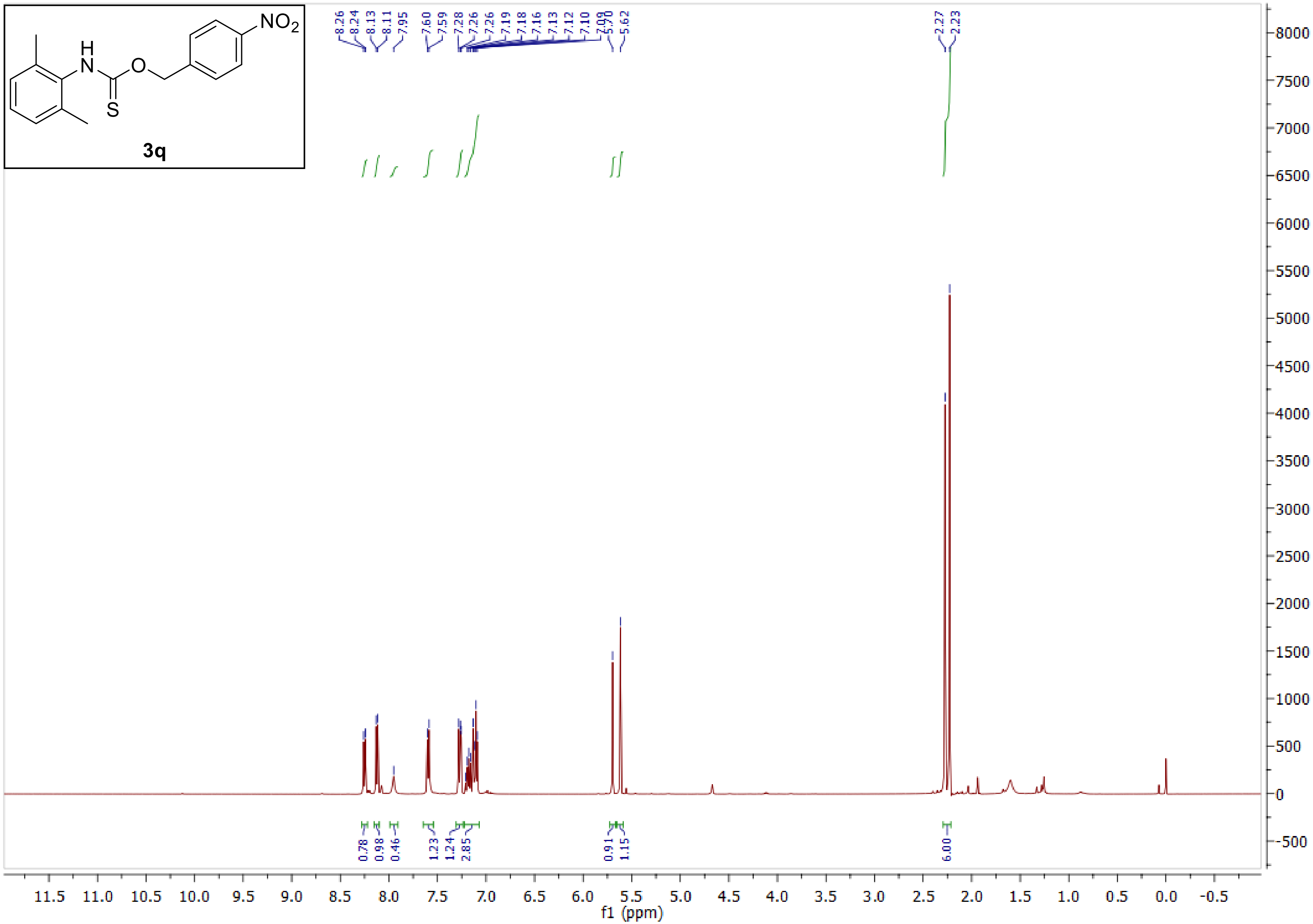

NO2
H
N
O
S
3q
8.26
8.24
8.13
8.11
7.95
7.60
7.59
7.28
7.26
7.19
7.18
7.16
7.13
7.12
7.10
7.09
5.70
5.62
2.27
2.23
0.78
0.98
0.46
1.23
1.24
2.85
0.91
1.15
6.00
f1 (ppm)

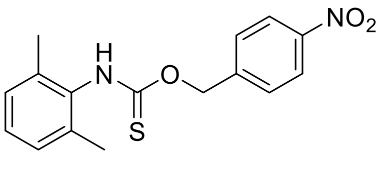

3q

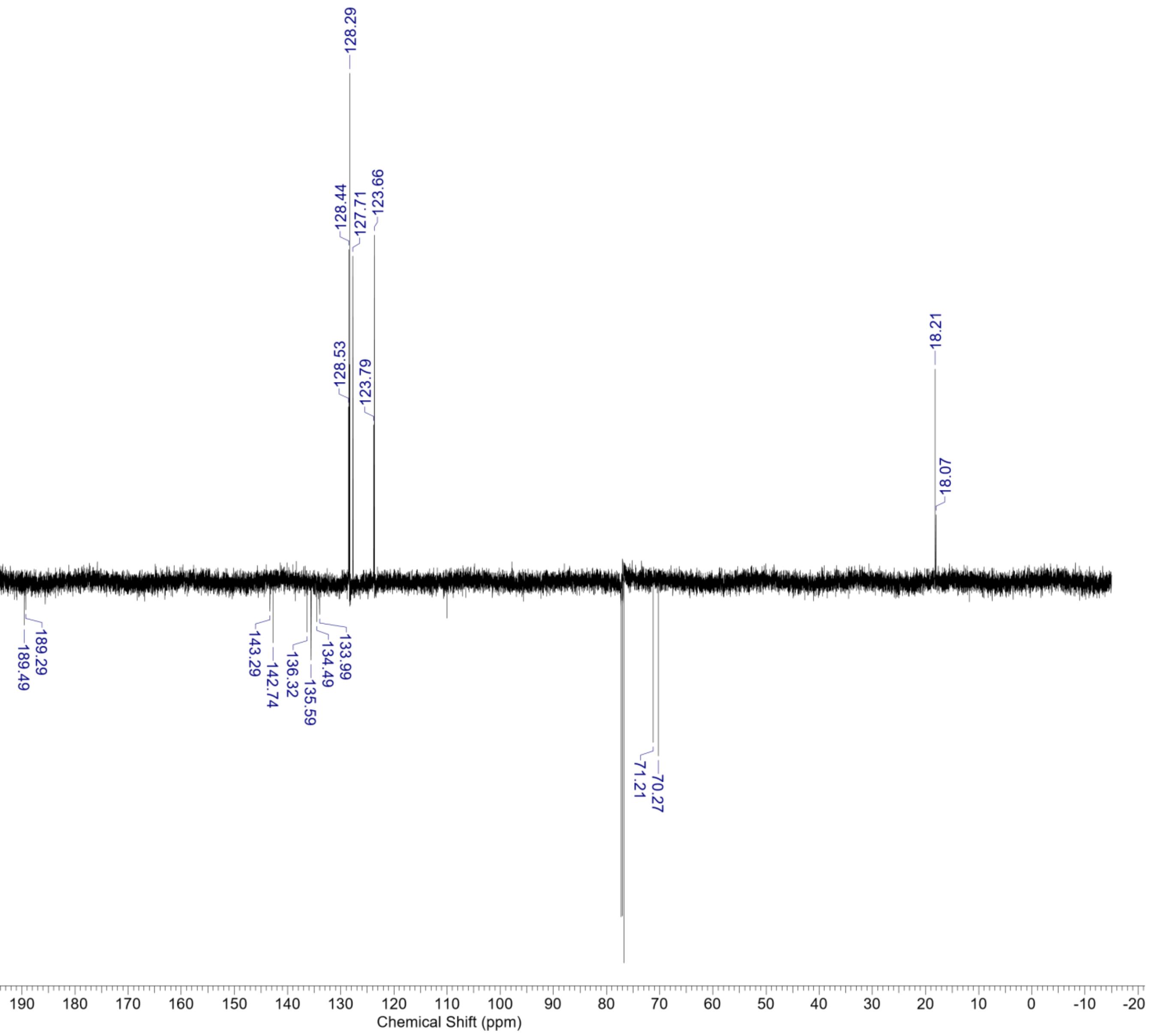

128.29
128.44
127.71
123.66
128.53
123.79
18.21
18.07
189.29
189.49
143.29
142.74
136.32
135.59
134.49
133.99
71.21
70.27
230 220 210 200 190 180 170 160 150 140 130 120 110 100 90 80 70 60 50 40 30 20 10 0 -10 -20
Chemical Shift (ppm)

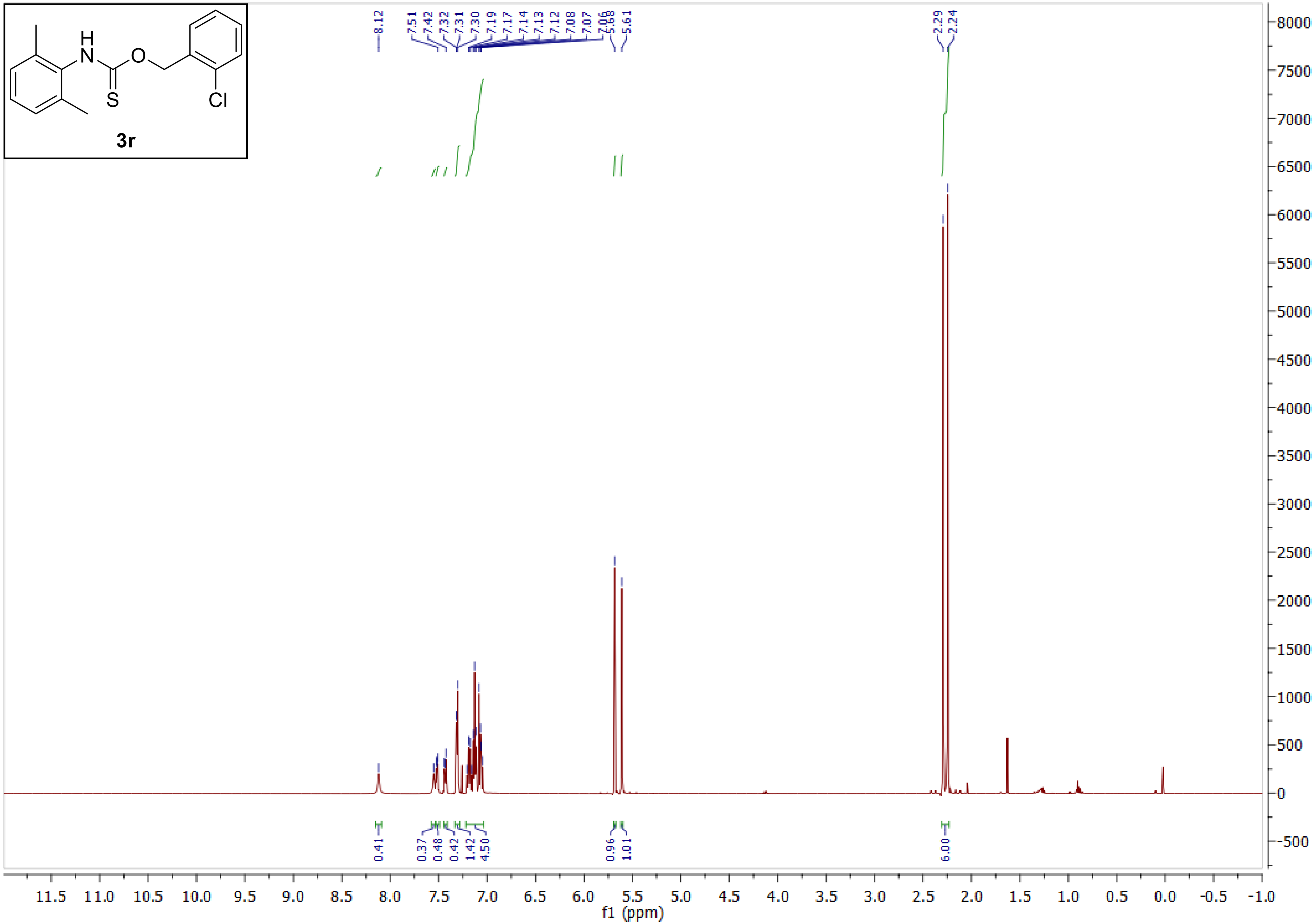

H
N
O
S
Cl
3r
8.12
7.51
7.42
7.32
7.31
7.30
7.19
7.17
7.14
7.13
7.12
7.08
7.07
7.06
5.68
5.61
2.29
2.24
0.41
0.37
0.48
0.42
1.42
4.50
0.96
1.01
6.00
f1 (ppm)

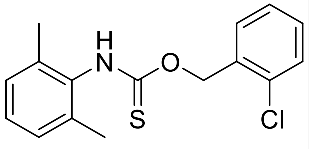
H
N
O
S
Cl

3r

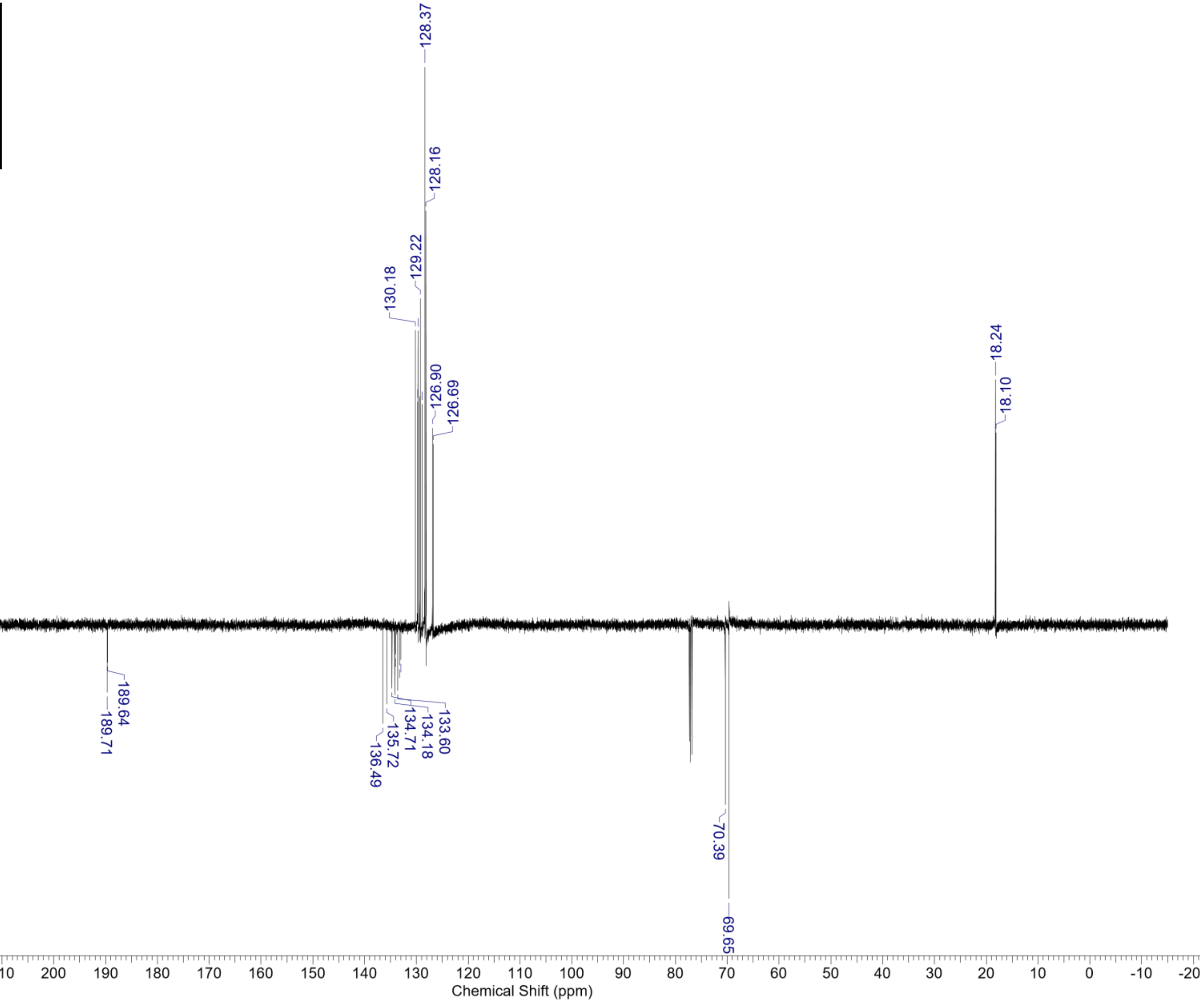
128.37
128.16
129.22
130.18
126.90
126.69
18.24
18.10
189.64
189.71
133.60
134.18
134.71
135.72
136.49
70.39
69.65
230 220 210 200 190 180 170 160 150 140 130 120 110 100 90 80 70 60 50 40 30 20 10 0 -10 -20
Chemical Shift (ppm)

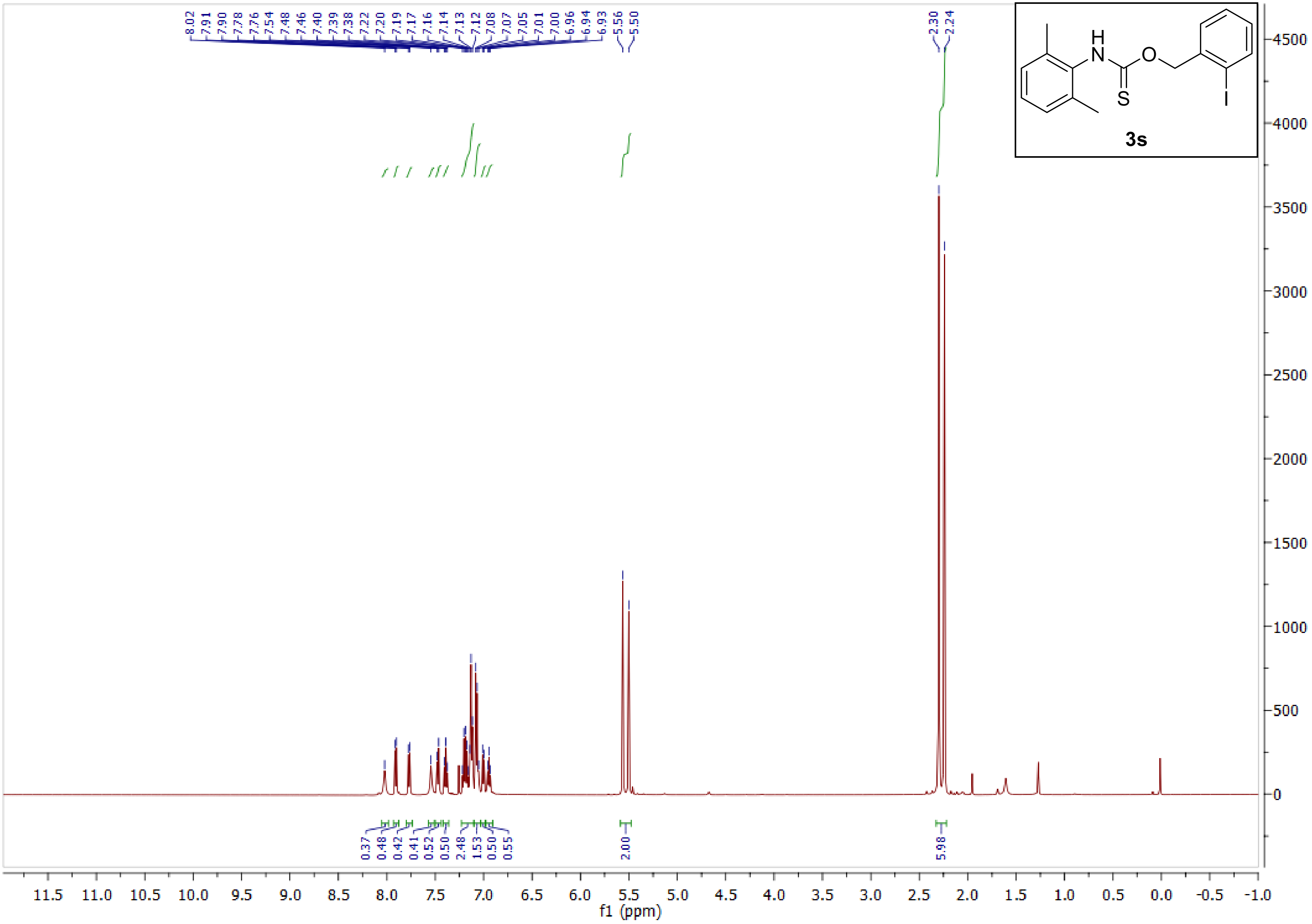

8.02
7.91
7.90
7.78
7.76
7.54
7.48
7.46
7.40
7.39
7.38
7.22
7.20
7.19
7.17
7.16
7.14
7.13
7.12
7.08
7.07
7.05
7.01
7.00
6.96
6.94
6.93
5.56
5.50
2.30
2.24
H
N
O
S
I
3s
0.37
0.48
0.42
0.41
0.52
0.50
2.48
1.53
0.50
0.55
2.00
5.98
f1 (ppm)

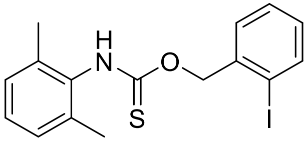
H
N
O
S
I

3s

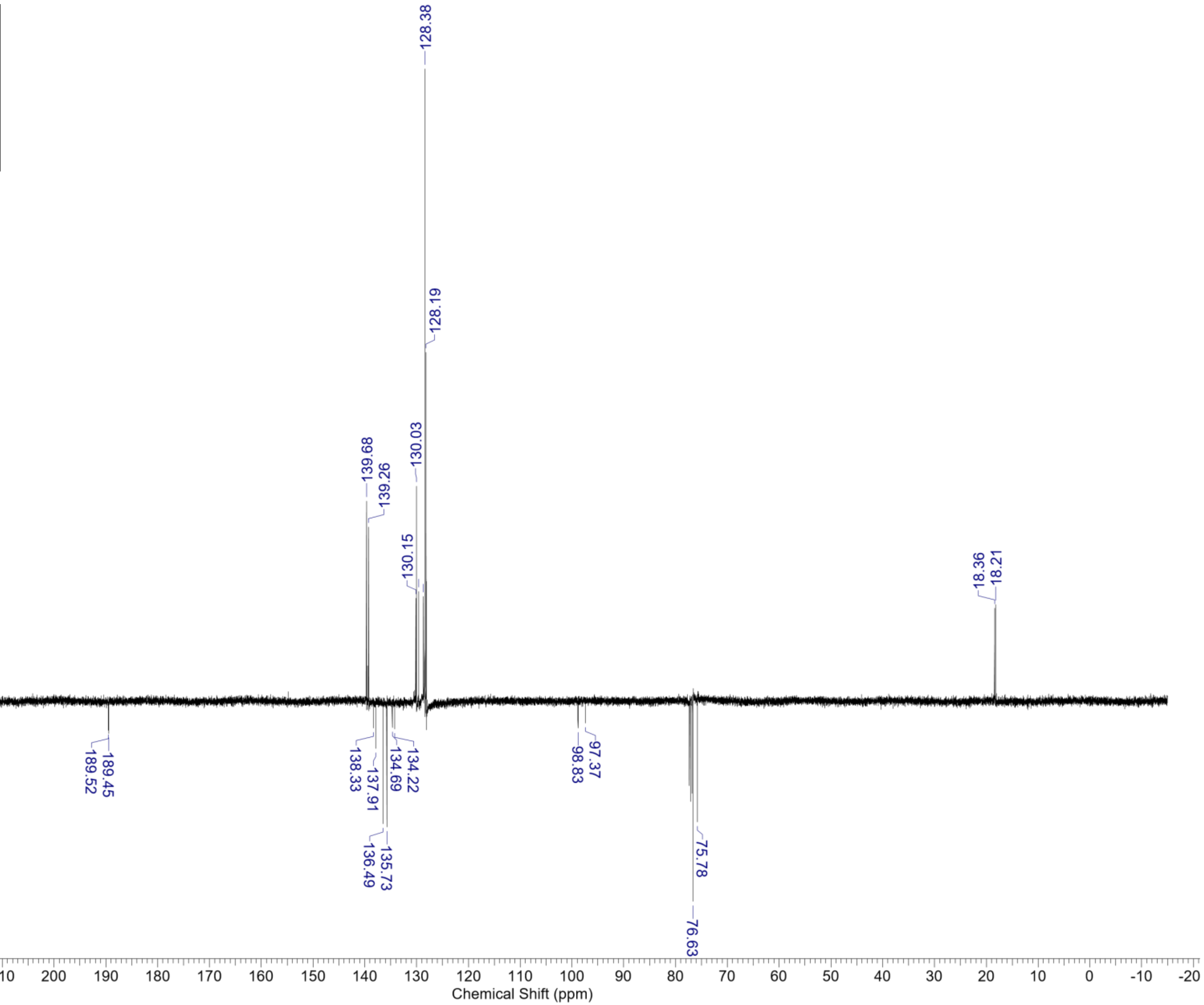
128.38
128.19
139.68
139.26
130.03
130.15
18.36
18.21
189.45
189.52
138.33
137.91
134.69
134.22
136.49
135.73
98.83
97.37
75.78
76.63
230 220 210 200 190 180 170 160 150 140 130 120 110 100 90 80 70 60 50 40 30 20 10 0 -10 -20
Chemical Shift (ppm)

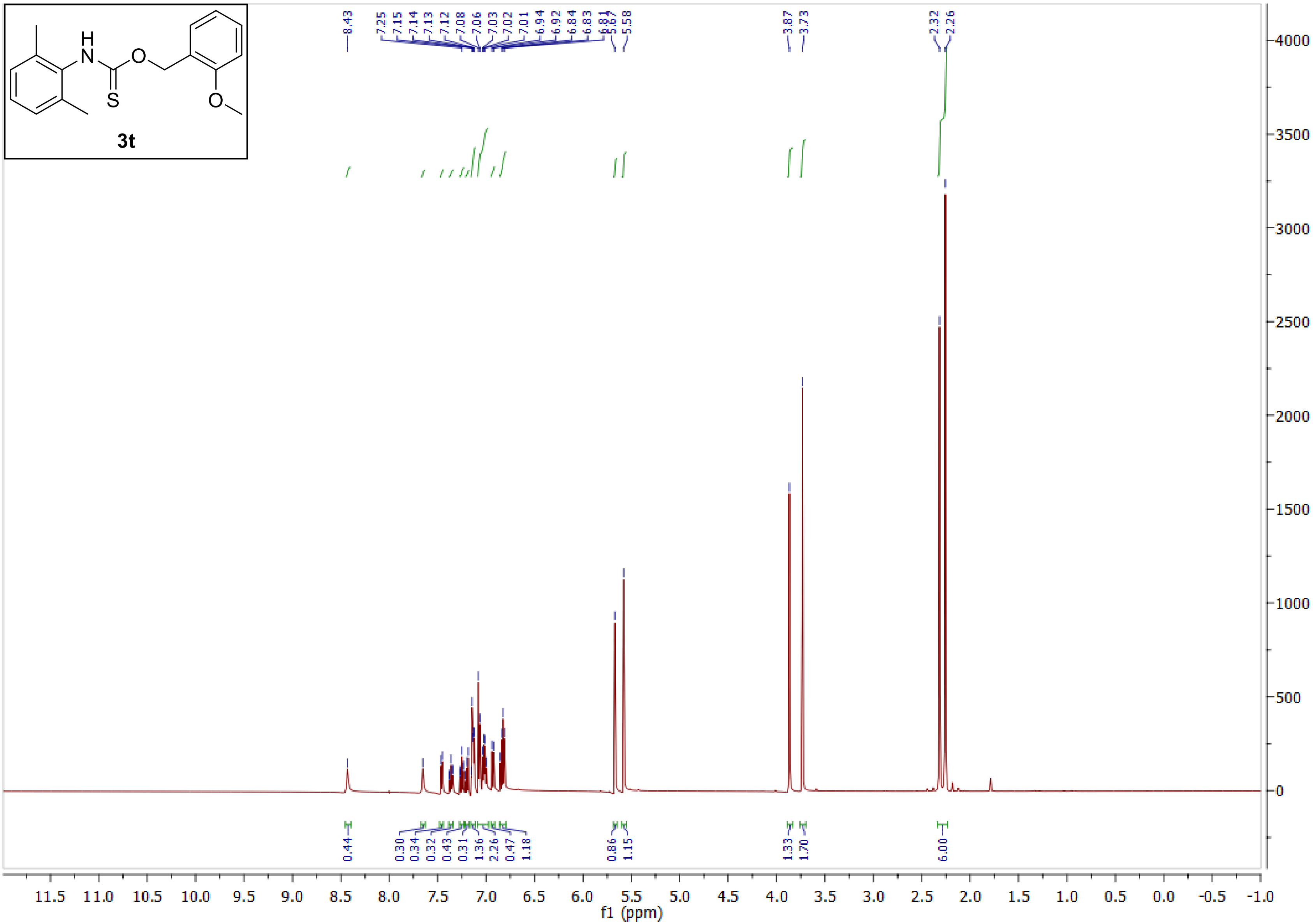

3t
8.43
7.25 7.15 7.14 7.13 7.12 7.08 7.06 7.03 7.02 7.01 6.94 6.92 6.84 6.83 6.81 5.67 5.58
3.87 3.73
2.32 2.26
0.44 0.30 0.34 0.32 0.43 0.31 1.36 2.26 0.47 1.18 0.86 1.15 1.33 1.70 6.00
f1 (ppm)

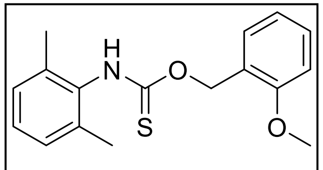

3t

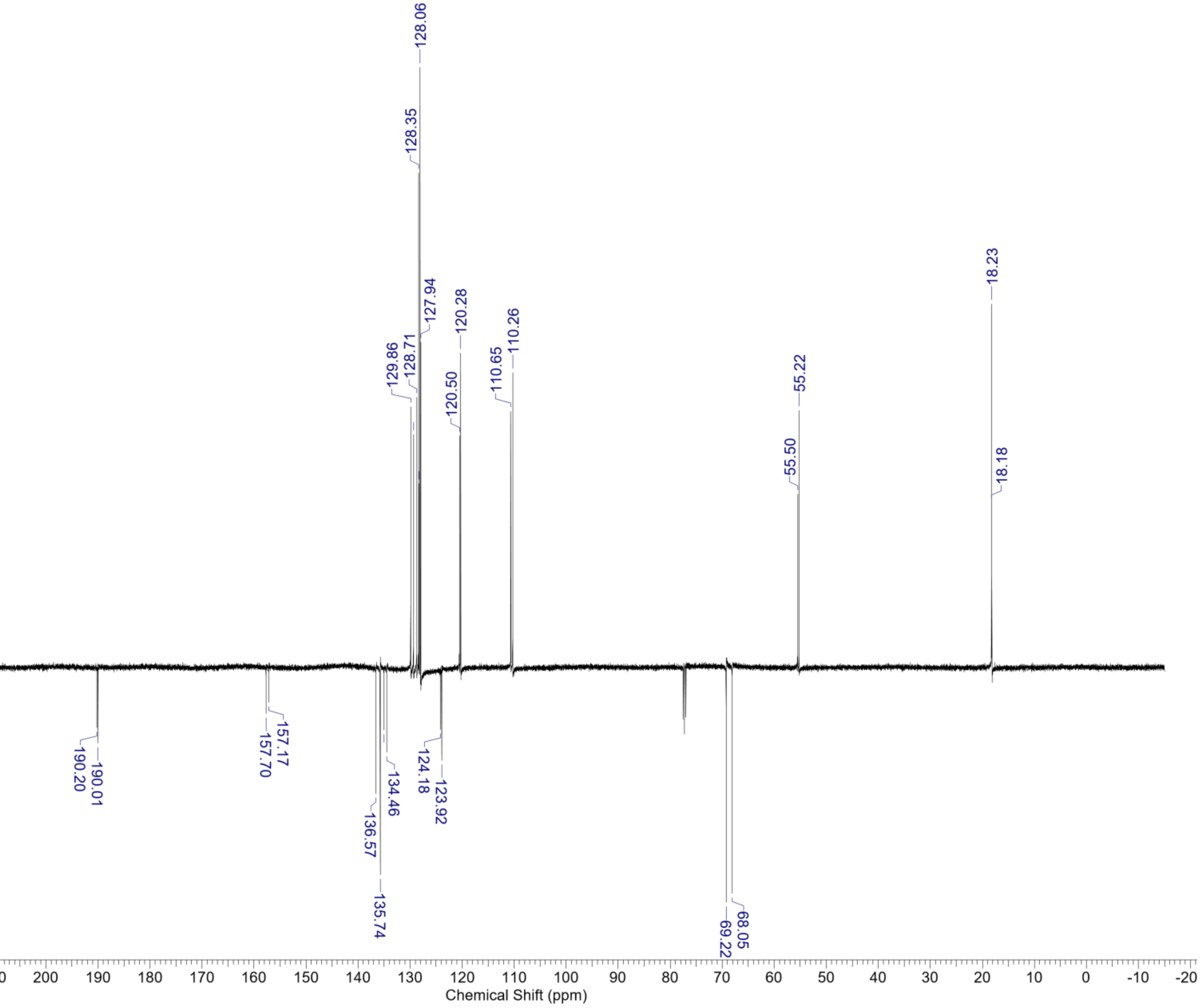

128.06
128.35
127.94
129.86
128.71
120.28
120.50
110.65
110.26
55.22
55.50
18.23
18.18
190.20
190.01
157.70
157.17
136.57
135.74
134.46
124.18
123.92
69.22
68.05
230 220 210 200 190 180 170 160 150 140 130 120 110 100 90 80 70 60 50 40 30 20 10 0 -10 -20
Chemical Shift (ppm)

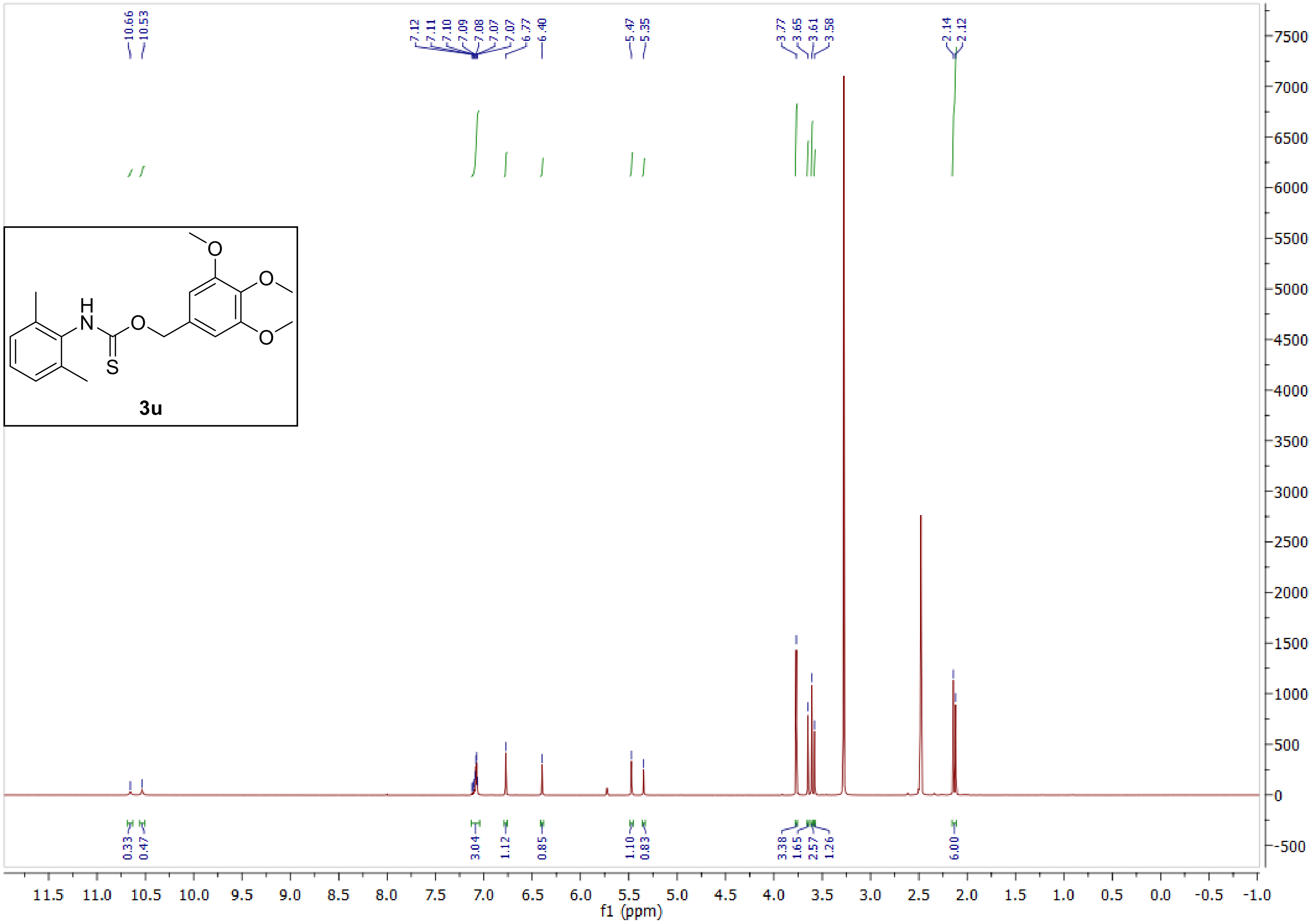
10.66
10.53
7.12
7.11
7.10
7.09
7.08
7.07
7.07
6.77
6.40
5.47
5.35
3.77
3.65
3.61
3.58
2.14
2.12
3u
0.33
0.47
3.04
1.12
0.85
1.10
0.83
3.38
1.65
2.57
1.26
6.00
f1 (ppm)

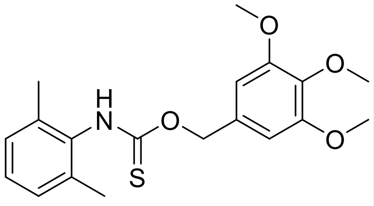
H
N
O
S
O
O
O

3u

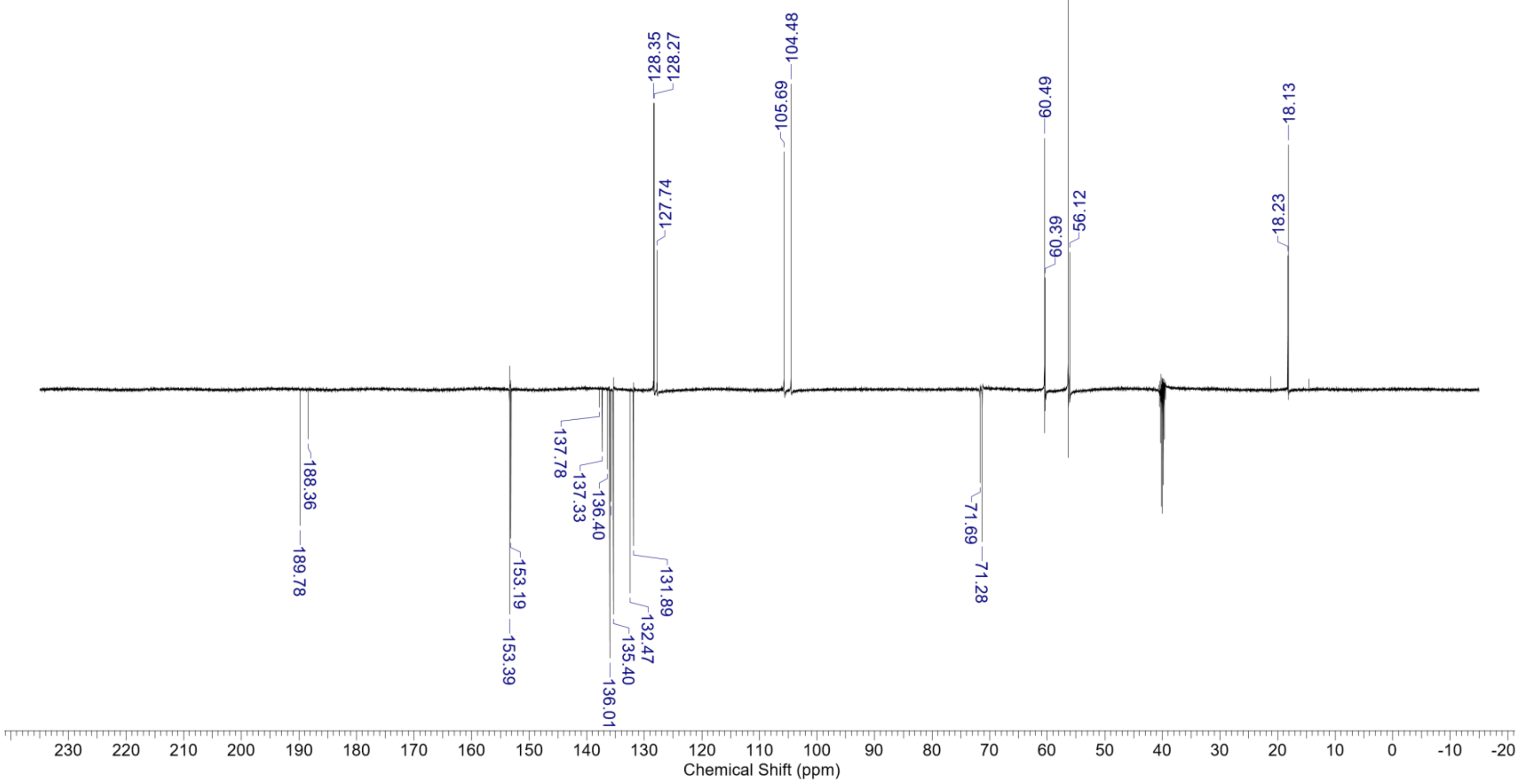
56.35
128.35
128.27
104.48
105.69
60.49
18.13
127.74
60.39
56.12
18.23
188.36
189.78
137.78
137.33
136.40
153.19
153.39
131.89
132.47
135.40
136.01
71.69
71.28
230
220
210
200
190
180
170
160
150
140
130
120
110
100
90
80
70
60
50
40
30
20
10
0
-10
-20
Chemical Shift (ppm)

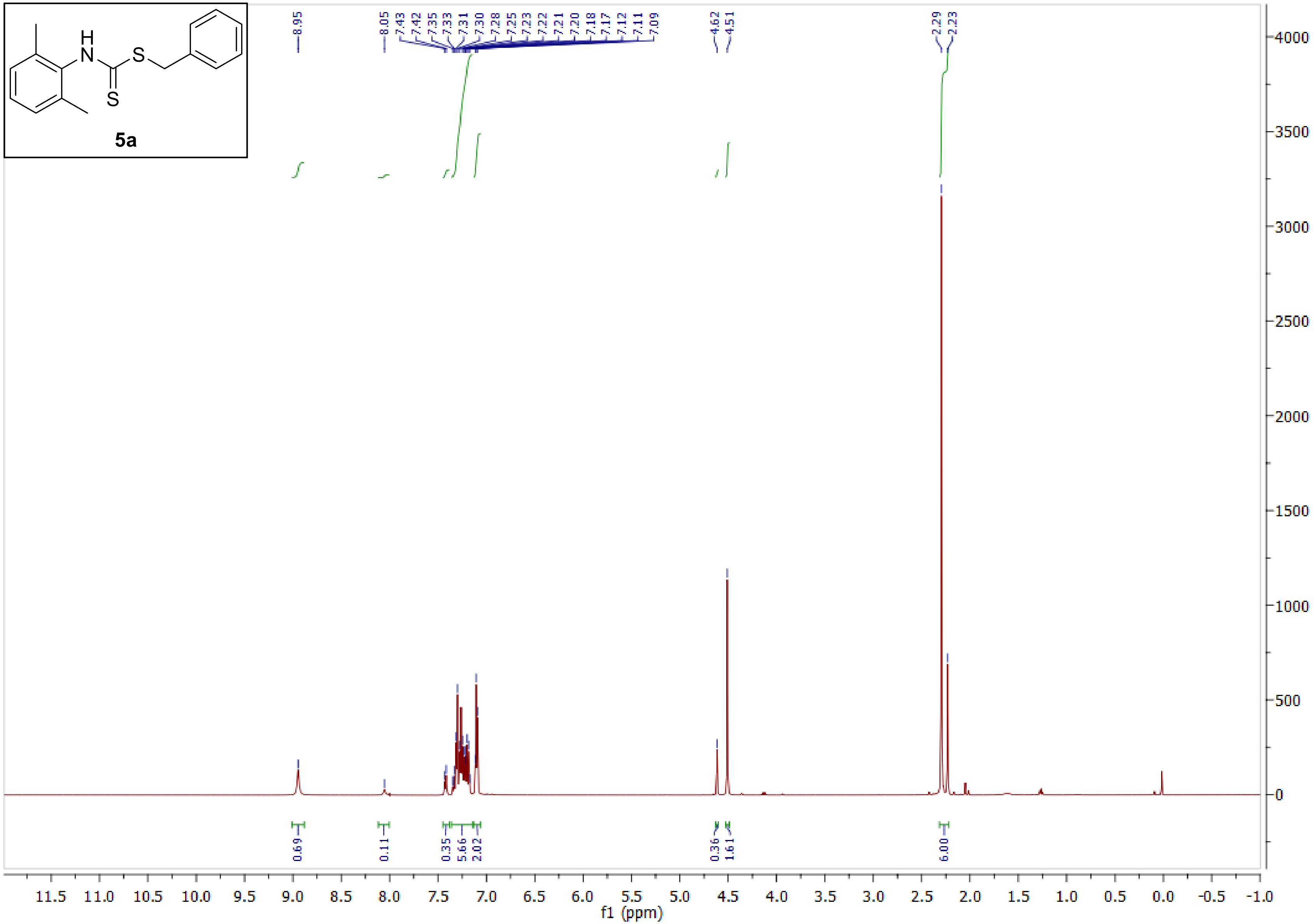
5a
8.95
8.05
7.43
7.42
7.35
7.33
7.31
7.30
7.28
7.25
7.23
7.22
7.21
7.20
7.18
7.17
7.12
7.11
7.09
4.62
4.51
2.29
2.23
0.69
0.11
0.35
5.66
2.02
0.36
1.61
6.00
f1 (ppm)

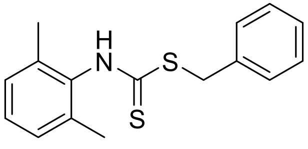
H
N
S
S

5a

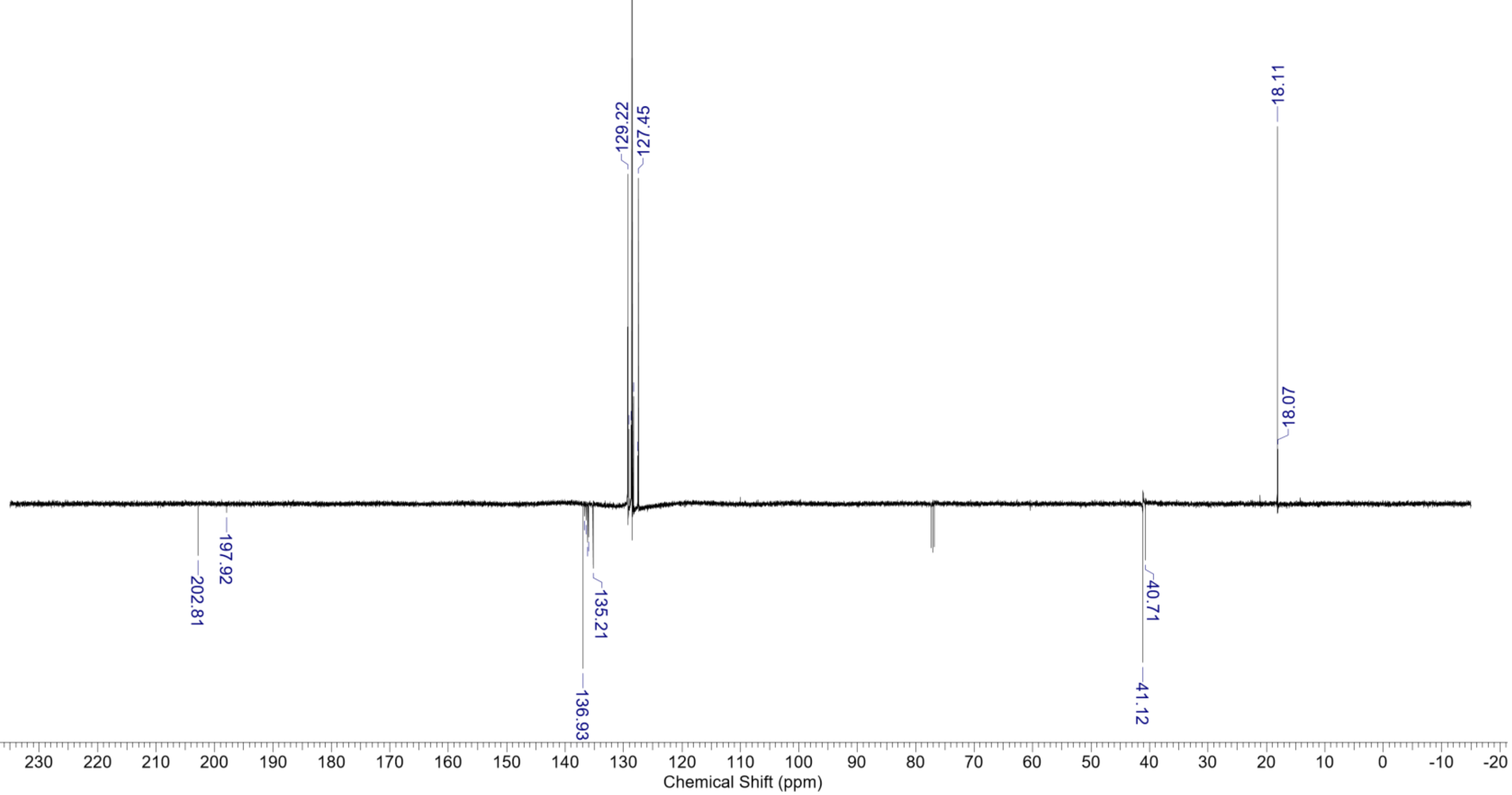
128.57
129.22
127.45
18.11
18.07
202.81
197.92
135.21
136.93
40.71
41.12
230 220 210 200 190 180 170 160 150 140 130 120 110 100 90 80 70 60 50 40 30 20 10 0 -10 -20
Chemical Shift (ppm)

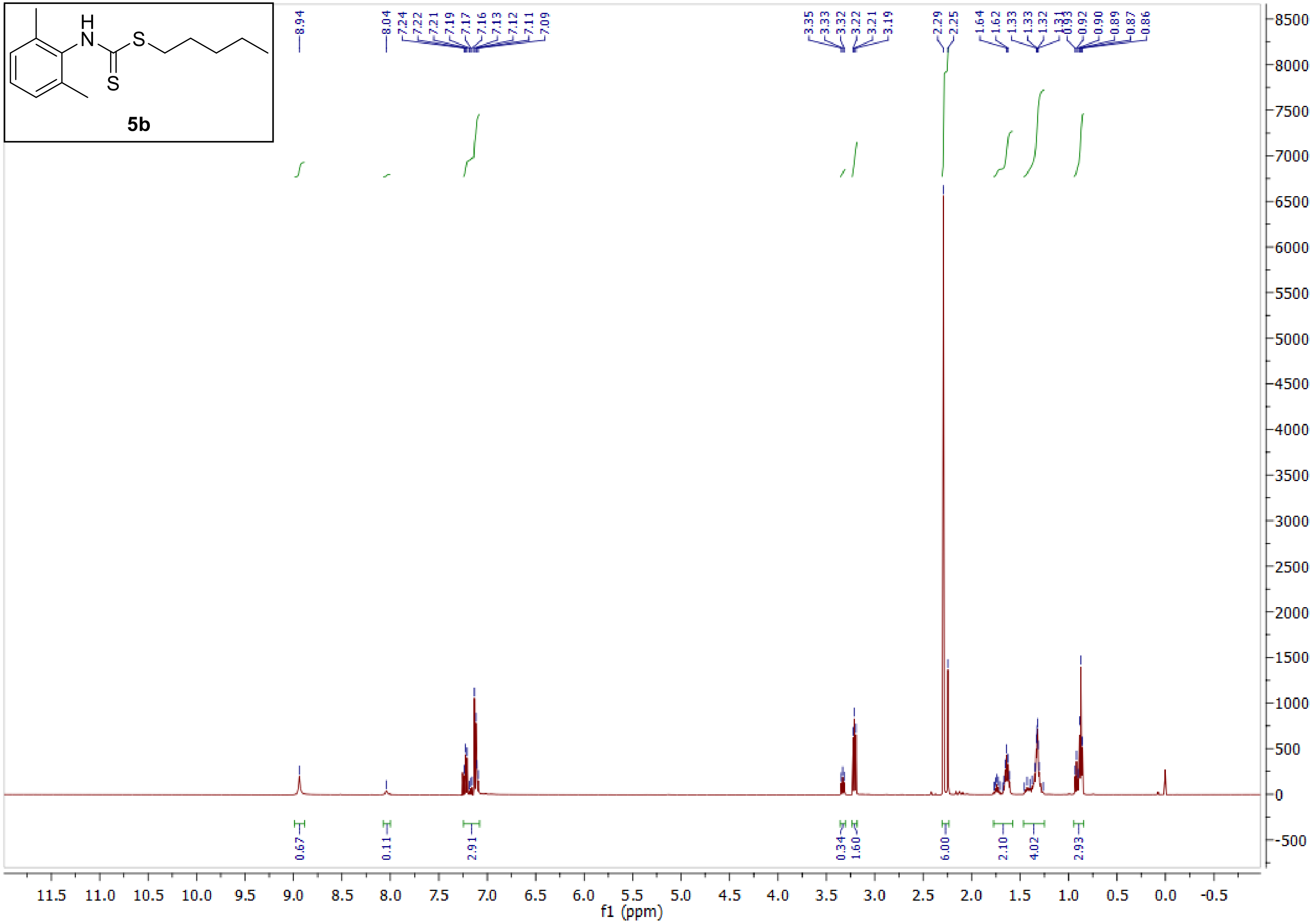

5b
8.94
8.04
7.24
7.22
7.21
7.19
7.17
7.16
7.13
7.12
7.11
7.09
3.35
3.33
3.32
3.22
3.21
3.19
2.29
2.25
1.64
1.62
1.33
1.33
1.32
1.31
0.93
0.92
0.90
0.89
0.87
0.86
0.67
0.11
2.91
0.34
1.60
6.00
2.10
4.02
2.93
f1 (ppm)

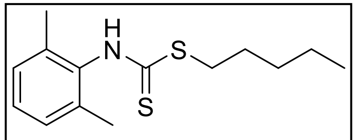
H
N
S
S

5b

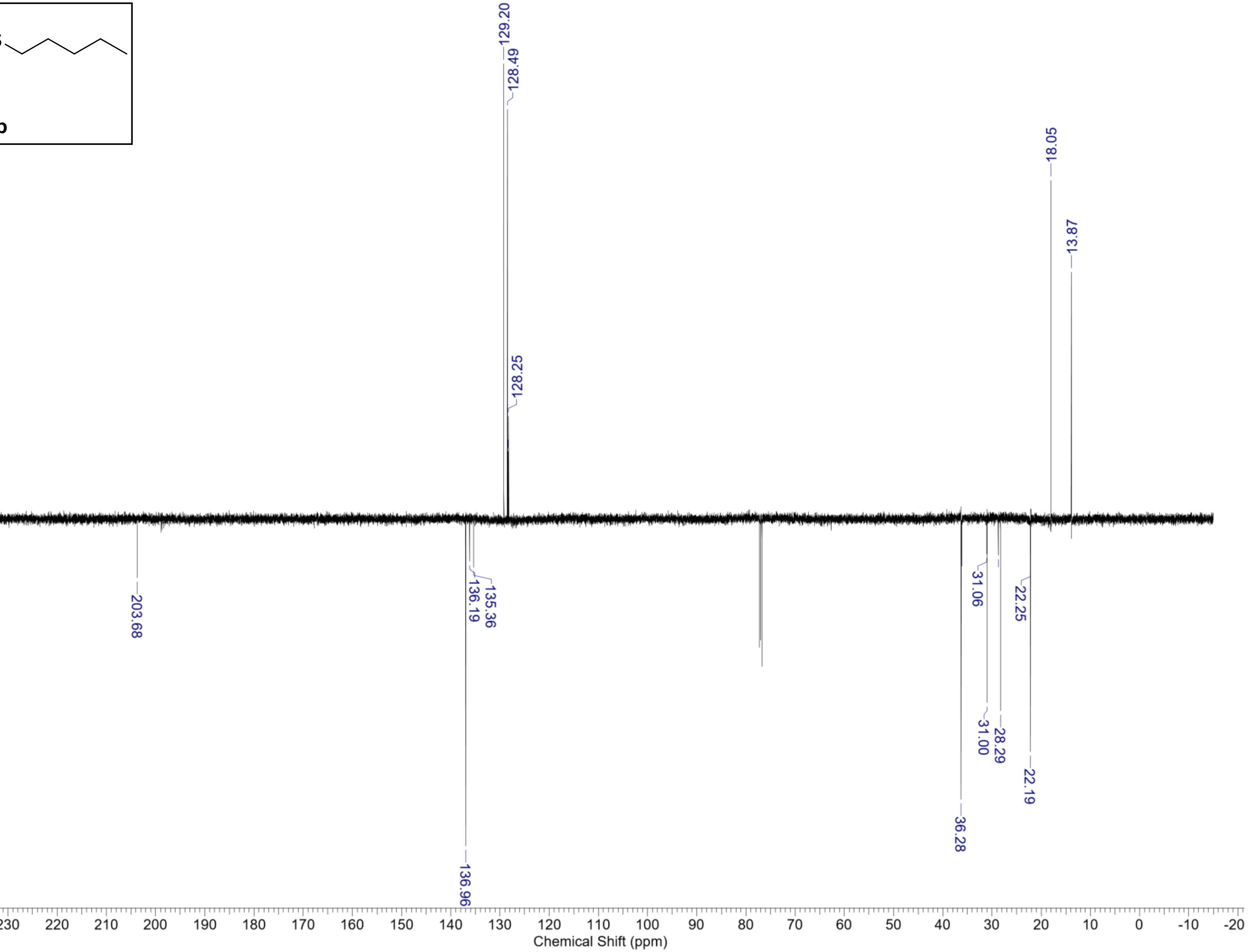
129.20
128.49
128.25
18.05
13.87
203.68
136.19
135.36
31.06
22.25
31.00
28.29
22.19
36.28
136.96
230 220 210 200 190 180 170 160 150 140 130 120 110 100 90 80 70 60 50 40 30 20 10 0 -10 -20
Chemical Shift (ppm)

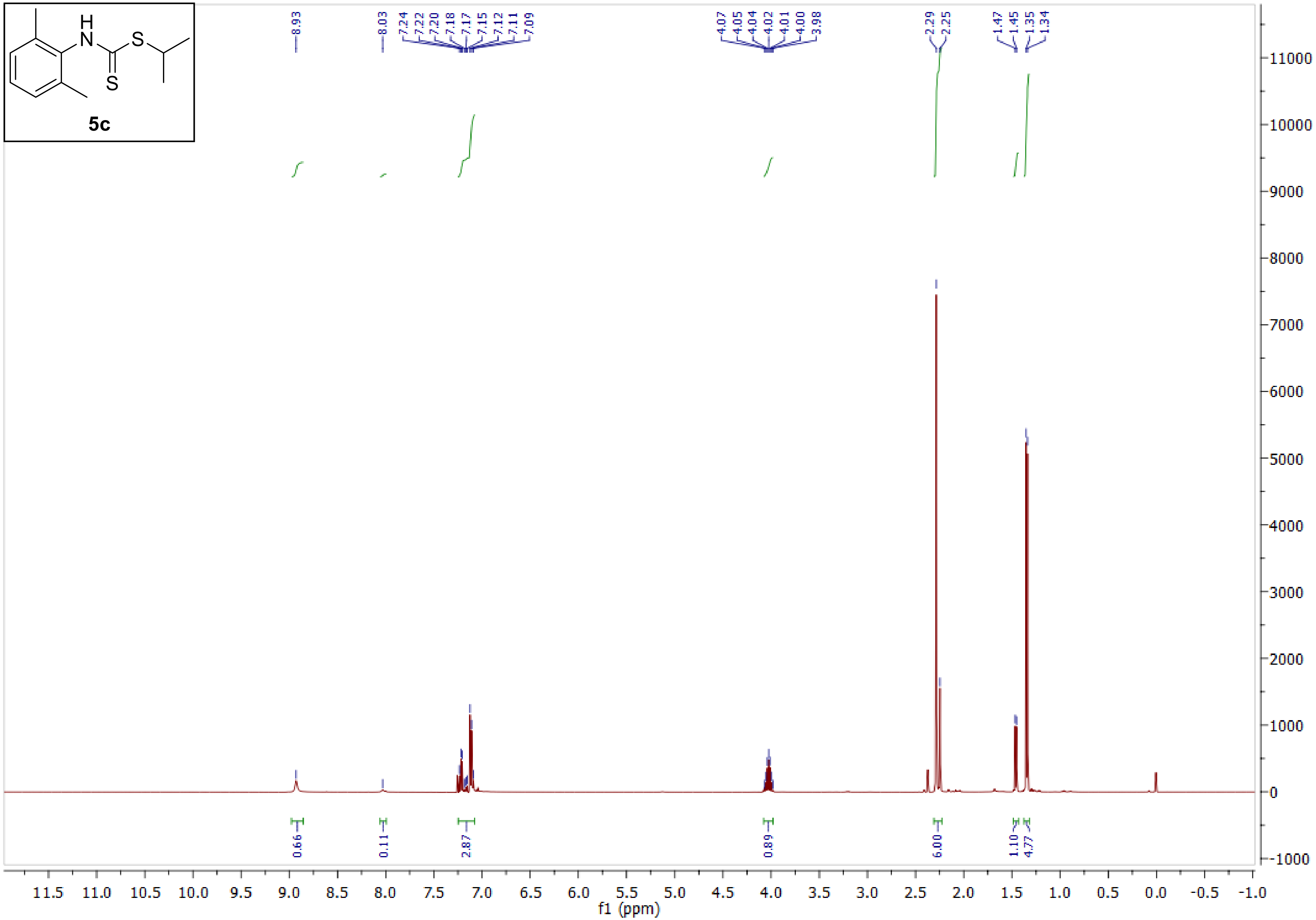
5c
8.93
8.03
7.24 7.22 7.20 7.18 7.17 7.15 7.12 7.11 7.09
4.07 4.05 4.04 4.02 4.01 4.00 3.98
2.29 2.25
1.47 1.45 1.35 1.34
0.66 0.11 2.87 0.89 6.00 1.10 4.77
f1 (ppm)

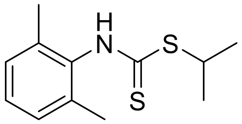
H
N
S
S

5c

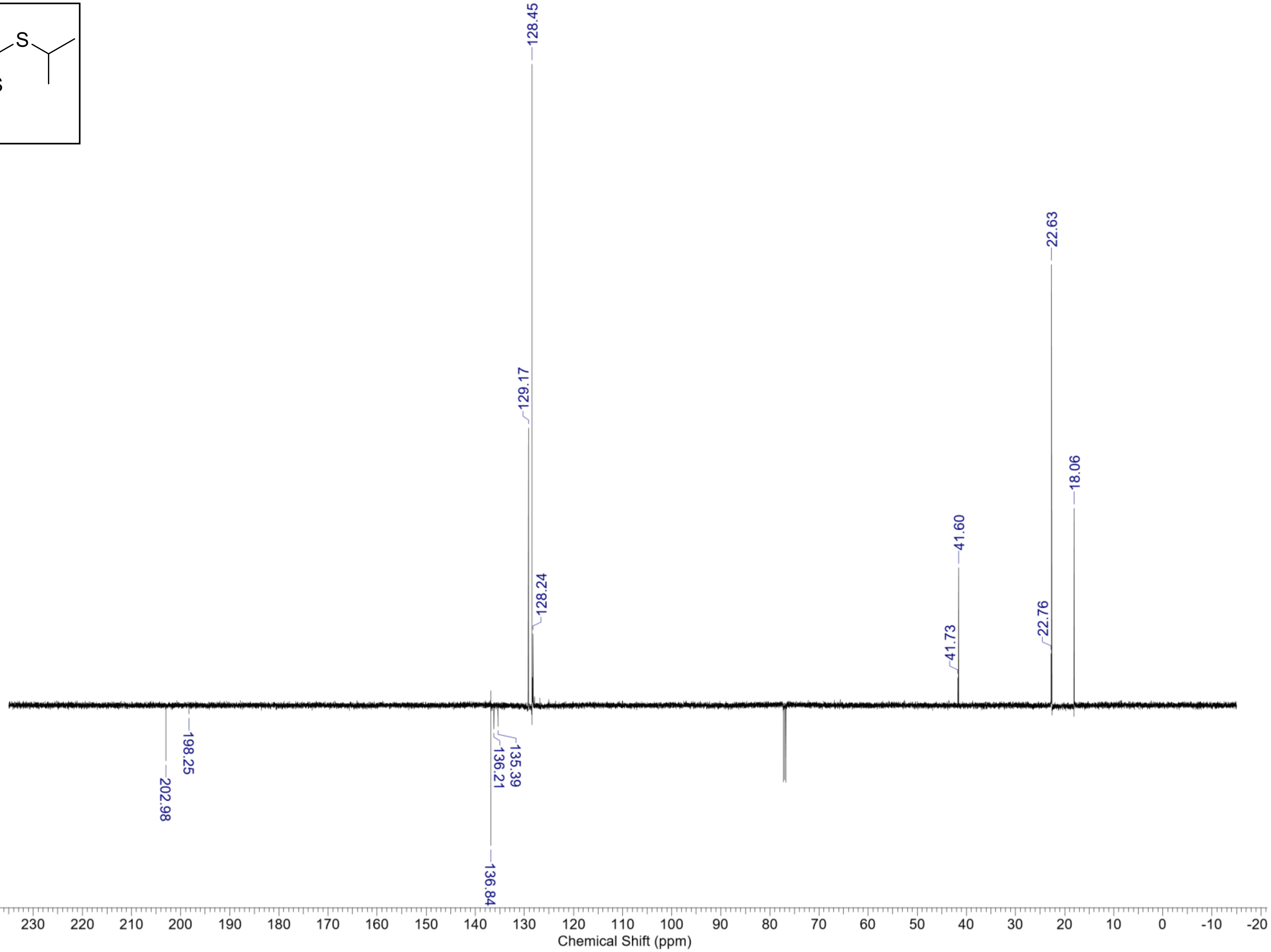
128.45
129.17
128.24
22.63
18.06
41.60
41.73
22.76
198.25
202.98
135.39
136.21
136.84
230 220 210 200 190 180 170 160 150 140 130 120 110 100 90 80 70 60 50 40 30 20 10 0 -10 -20
Chemical Shift (ppm)

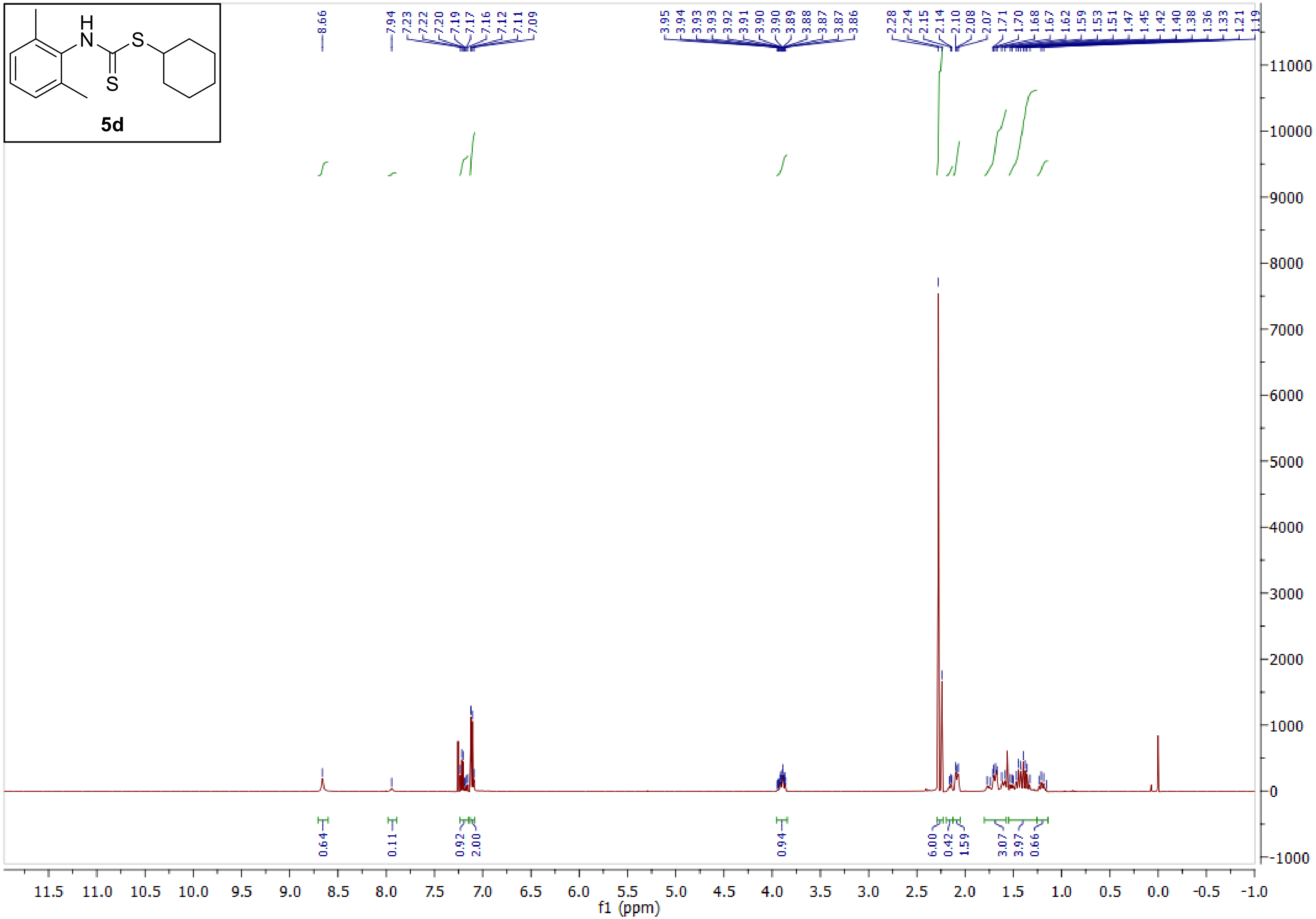
5d
f1 (ppm)

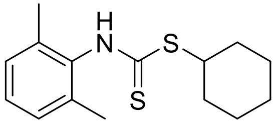

5d

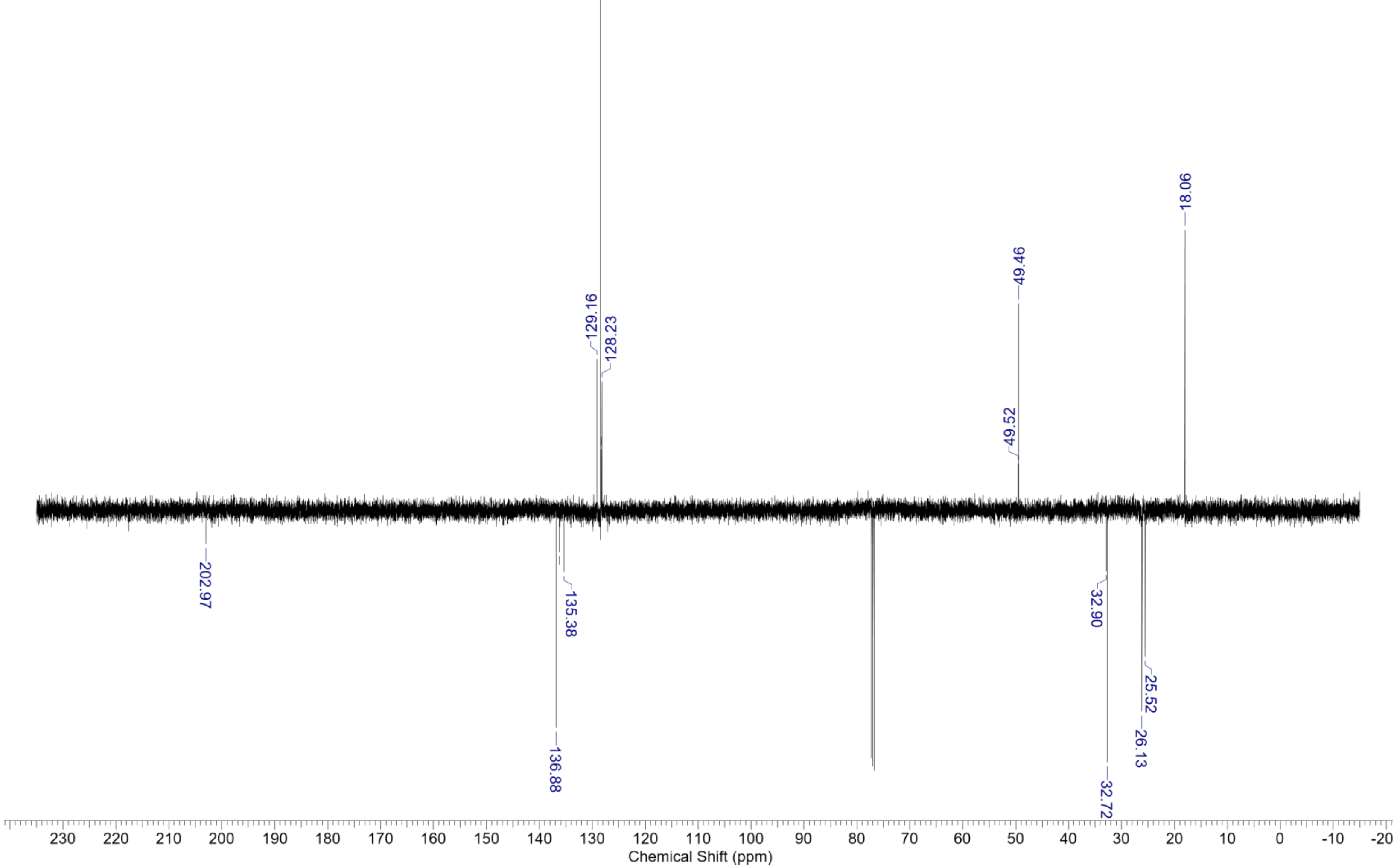
128.45
129.16
128.23
49.46
49.52
18.06
202.97
135.38
136.88
32.90
32.72
25.52
26.13
230 220 210 200 190 180 170 160 150 140 130 120 110 100 90 80 70 60 50 40 30 20 10 0 -10 -20
Chemical Shift (ppm)

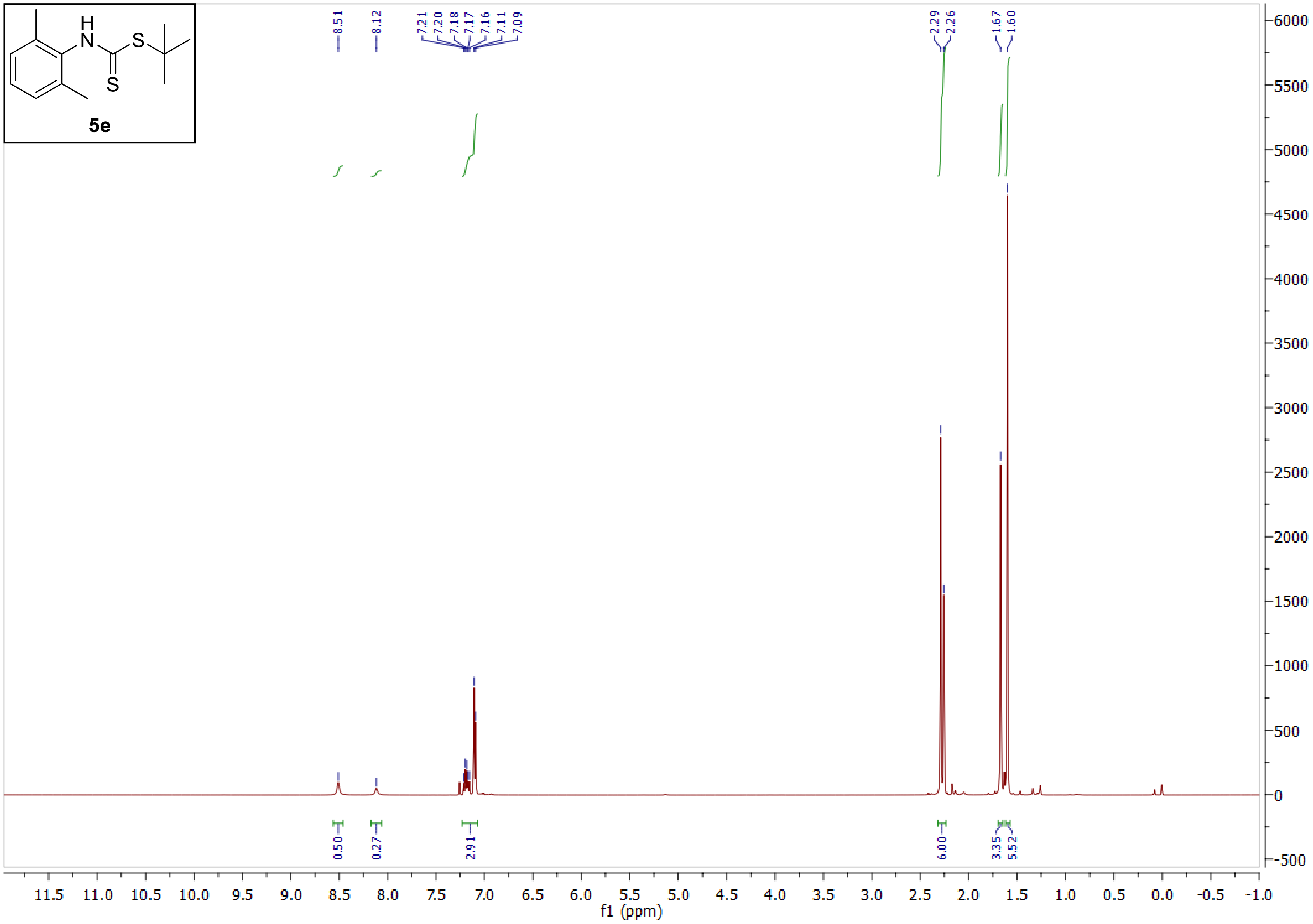

5e
8.51
8.12
7.21
7.20
7.18
7.17
7.16
7.11
7.09
2.29
2.26
1.67
1.60
0.50
0.27
2.91
6.00
3.35
5.52
f1 (ppm)

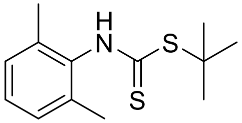
H
N
S
S

5e

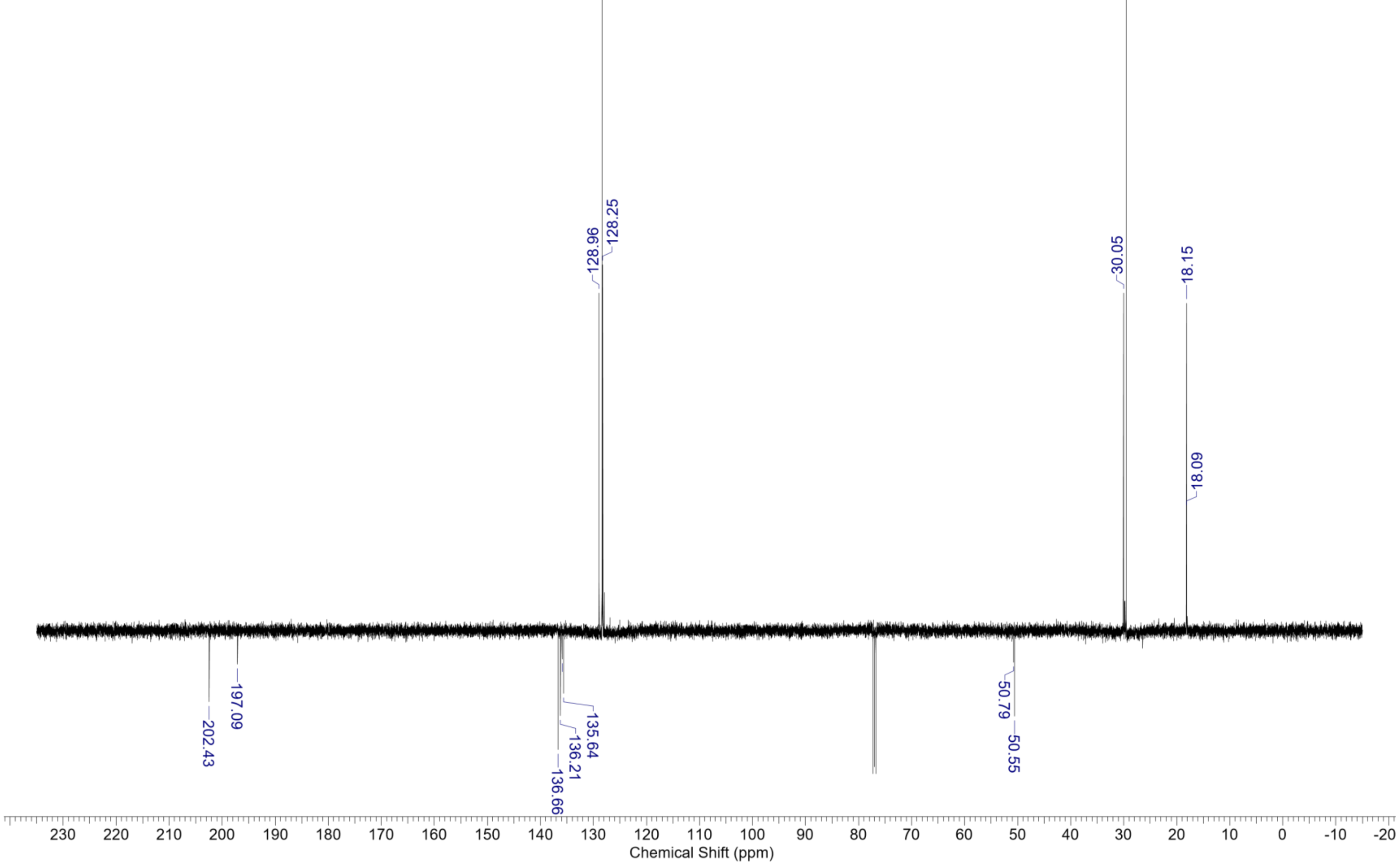
128.37
29.48
128.96
128.25
30.05
18.15
18.09
202.43
197.09
135.64
136.21
136.66
50.79
50.55
230 220 210 200 190 180 170 160 150 140 130 120 110 100 90 80 70 60 50 40 30 20 10 0 -10 -20
Chemical Shift (ppm)

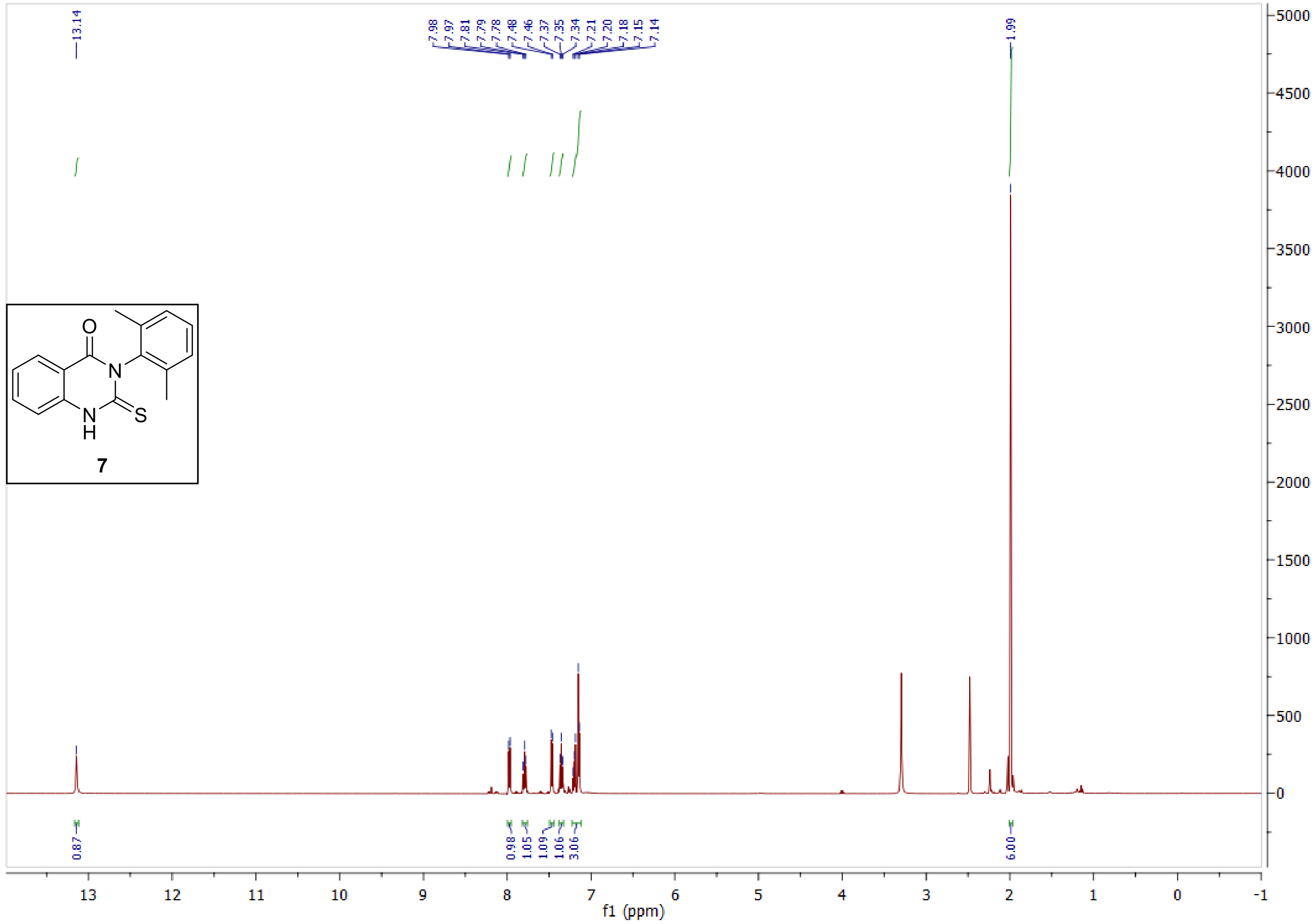

13.14
7.98
7.97
7.81
7.79
7.78
7.48
7.46
7.37
7.35
7.34
7.21
7.20
7.18
7.15
7.14
1.99
O
N
N
H
S
7
0.87
0.98
1.05
1.09
1.06
3.06
6.00
f1 (ppm)

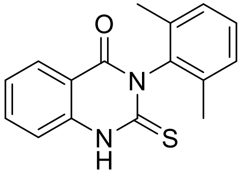

7

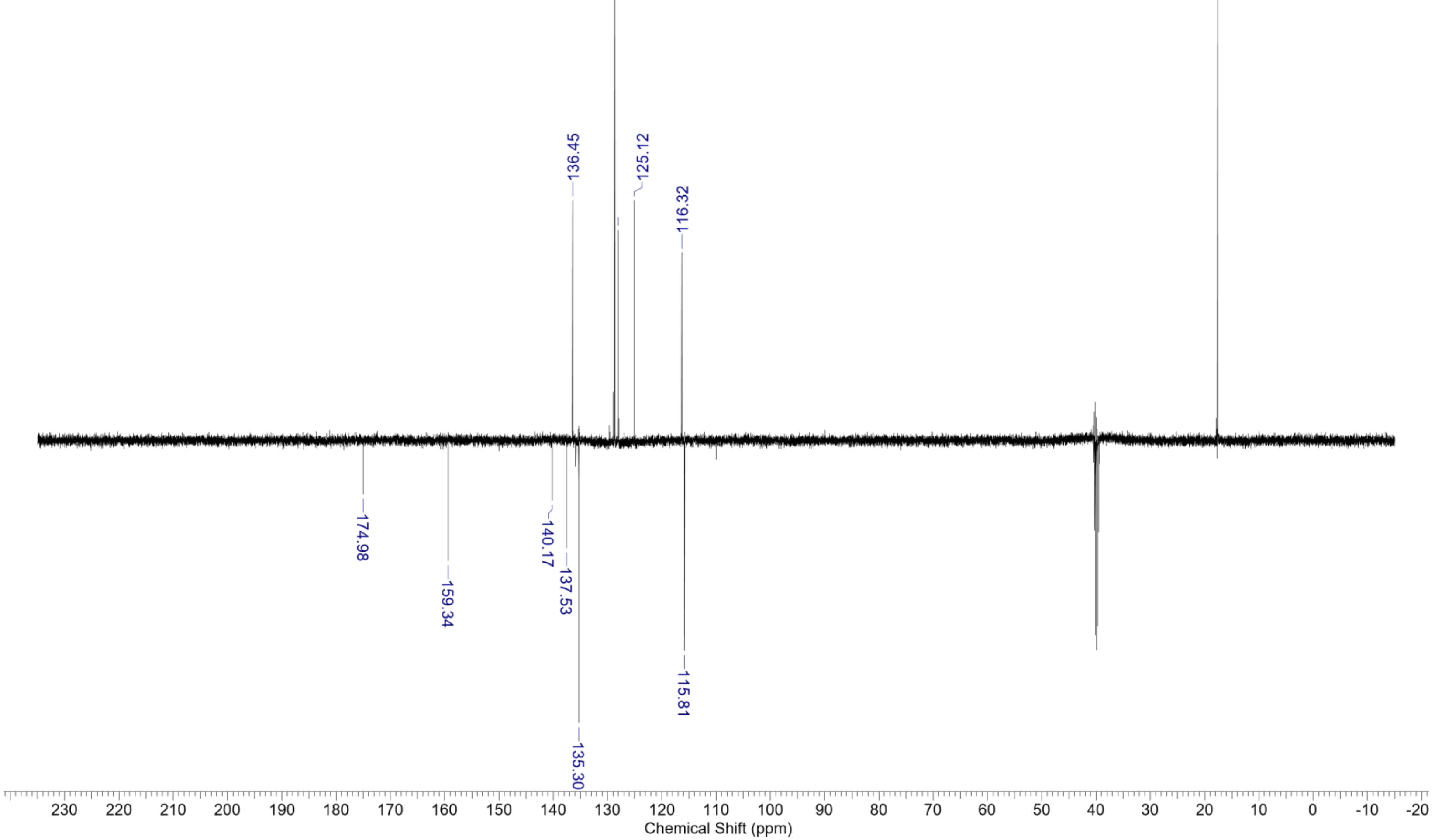
128.68
17.63
136.45
125.12
116.32
174.98
159.34
140.17
137.53
115.81
135.30
230 220 210 200 190 180 170 160 150 140 130 120 110 100 90 80 70 60 50 40 30 20 10 0 -10 -20
Chemical Shift (ppm)

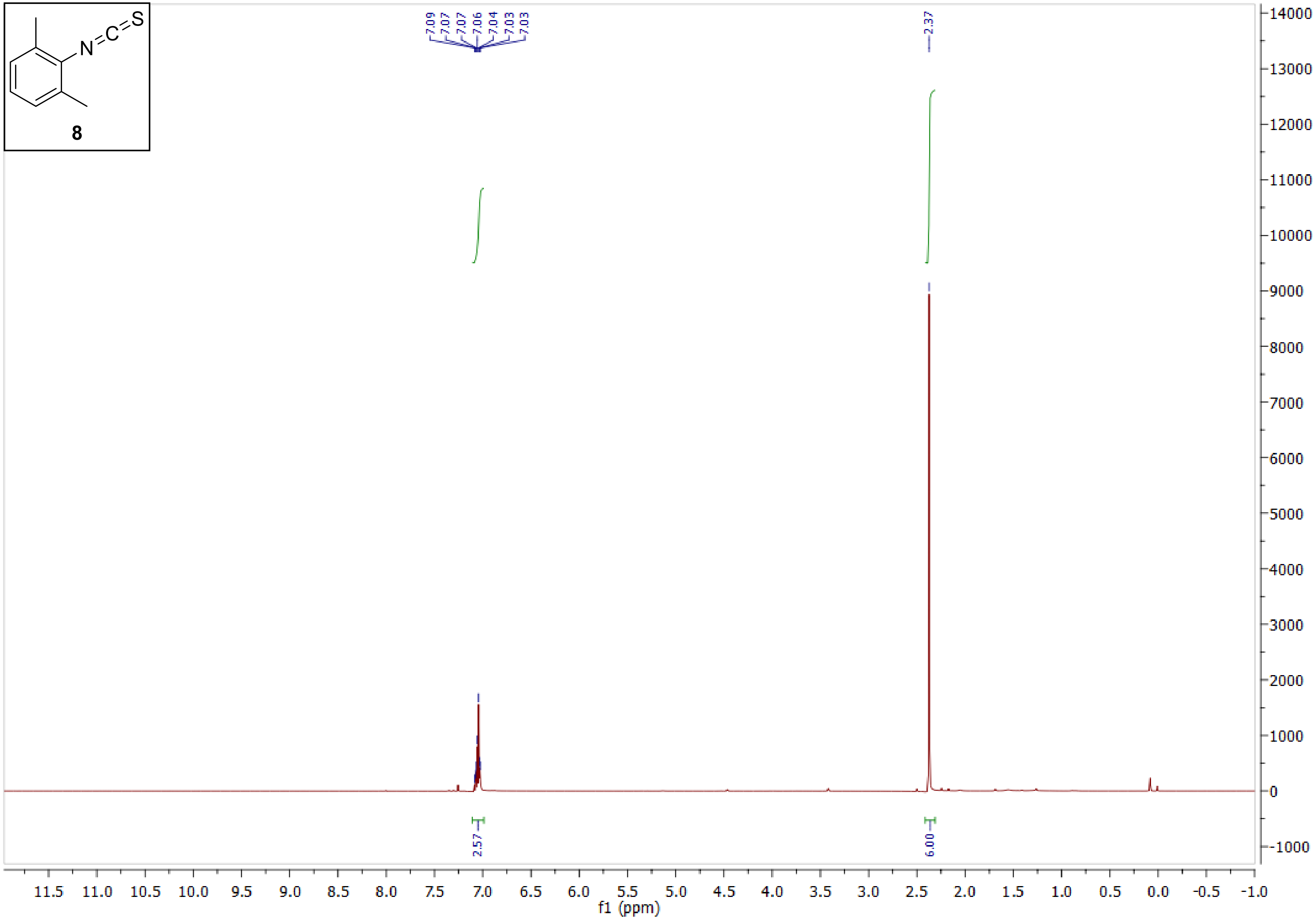
N=C=S
8
7.09
7.07
7.07
7.06
7.04
7.03
7.03
2.37
2.57
6.00
f1 (ppm)

References

1. Chniti, I.; Sanhoury, M. A. K.; Cehidi, I. *J. Fluorine Chem.* **2013,** *156,* 101–105.
2. Burrows, A. A.; Hunter, L. *J. Chem. Soc.*, **1952**, *0*, 4118–4122.
3. Degani, I.; Fochi, R.; Magistris, C. *Synthesis*, **2009**, 3807–3818.
4. L'abbé, G.; Sorgeloos, D.; Toppet, S.; King, G. S. D.; Van Meerwelt, L. *B. Soc. Chim. Belg.*, **1981**, *90*, 63–74.
5. Maiti, D.; Woertink, J. S.; Vance, M. A.; Milligan, A. E.; Narducci, S. A. A.; Solomon, E. I.; Karlin, K. D. *J. Am. Chem. Soc.*, **2007**, *129*, 8882–8892.
